# Synthesis, toxicological and in silico evaluation of novel spiro pyrimidines against *Culex pipiens* L. referring to chitinase enzyme

**DOI:** 10.1038/s41598-024-51771-8

**Published:** 2024-01-17

**Authors:** Eslam M. Abbass, Ali Khalil Ali, Ahmed F. El-Farargy, Doaa R. Abdel-Haleem, Safaa S. Shaban

**Affiliations:** 1 Chemistry Department, Faculty of Science, Ain Shams University, Abbassia, 11566 Cairo Egypt; 2 Chemistry Department, Faculty of Science, Zagazig University, Zagazig, 44519 Egypt; 3 Entomology Department, Faculty of Science, Ain Shams University, Abbassia, 11566 Cairo Egypt

**Keywords:** Biochemistry, Chemistry

## Abstract

The exponential development of resistance to conventional chemical insecticides adds another important motive for the creation of novel insecticidal active agents. One of the keys to meeting this challenge is the exploration of novel classes of insecticidal molecules with different modes of action. Herein, a novel series of spiro pyrimidine derivatives was prepared using some green synthetic methodologies such as microwave irradiation, and sonication under ultrasound waves. Spiro pyrimidine aminonitrile **1** is a key starting material for the synthesis of targets **2–9** by reaction with different carbon electrophiles and nitrogen nucleophiles. The structures of all the newly synthesized compounds were approved using spectral data. The toxicological efficiency and biological impacts of the synthesized spiro pyrimidine derivatives were assessed against *Culex pipiens* L. larvae. The toxicity of synthesized compounds showed remarkable variations against the *C. pipiens* larvae. Where, **3**, **4** and **2** were the most efficient compounds with LC_50_ values of 12.43, 16.29 and 21.73 µg/mL, respectively. While **1** was the least potent compound with an LC_50_ value of 95.18 µg/mL. As well, other compounds were arranged according to LC_50_ values as follows **5** > **7** > **6** > **9** > **8**. In addition, **3** and **4** exhibited significant prolongation of the developmental duration and greatly inhibited adult emergence. Moreover, many morphological deformities were observed in all developmental stages. Furthermore, cytotoxicity of the most effective compounds was assessed against the normal human cells (WI-38) as non-target organisms, where compounds **2**, **4** and **3** showed weak to non-toxic effects. The study of binding affinity and correlation between chemical structure and reactivity was carried out using molecular docking study and DFT calculations to investigate their mode of action. This study shed light on promising compounds with larvicidal activity and biological impacts on the *C. pipiens* life cycle.

## Introduction

Mosquitos are dreadful vectors for many grave diseases and are attributed to serious socioeconomic issues. In developing countries, more than 1 billion people are living at risk from these diseases^1^. *Culex pipiens* (Linnaeus) (Diptera: Culicidae) is the most common species that occur in urban, peri-urban, and rural habitats^2^. It is considered one of the most important public health insect vectors and plays a primary role in many disease transmissions as bancroftian filariasis, West Nile virus and Rift Valley fever virus^3,4^. Frequent and prolonged use of traditional insecticides imposes an enormous selection in favor of insecticide-resistance mosquito populations and will usually lead to reduced efficacy of the chemical insecticide classes and limit the available mosquito control options^5^. The most recommended mosquito-borne disease control strategy is combatting the intermediate vector host (mosquito) at either the immature or adult stages^1^.

Hence, larval control is critical and relies on different synthetic larvicides and IGRs such as pyriproxifen^6^. Pyriproxyfen is a hormone analogue that interferes with the metamorphosis of immature mosquitoes, obstructing their development into adults capable of transmitting diseases^7^. Consequently, there are ongoing efforts to develop and discover new chemicals with insecticidal potencies due to the loss of existing traditional chemicals through the buildup of insect resistance. Thus, screening of new compounds with a novel mode of action and the desire for chemicals with more favourable toxicological and environmental profiles are required.

The pyrimidine derivatives are attributed to the development of many agrochemicals due to their high insecticidal activity and many commercial insecticides containing pyrimidine moiety such as pirimiphos-methyl and diflubenzuron^1^. Pyrimidine and its derivatives occupy a unique role in medicinal chemistry due to their wide application as drug and drug-intermediates possessing diverse pharmacological activities including antitumor, analgesic, antioxidant, antiviral, and antiallergic activities^8–12^. Similarly, the related pyrimidine thione therapeutic derivatives have been known as potential antiviral, antioxidant, anticancer, and antimicrobial agents^13–16^.

In addition, spiro compounds are a class of organic compounds that are characterized by a unique chemical structure in which two or more rings share a single common atom^17^. These compounds possess biological activities and are attributed to a wide variety of natural products and drugs^18–20^, including insecticidal activity^21^. Spiro compounds showed insecticidal activity against mosquitoes and houseflies^22^. Some spiro derivatives showed excellent acaricidal efficiency against *Tetranychus cinnabarinus* and good insecticidal potency against different insect pests^23^.

Therefore, in our work, we target to design and synthesize a new series of spiro pyrimidine derivatives and evaluate their insecticidal toxicity against *C. pipiens* larvae and their biological effects on the *C. pipiens* life cycle. As well, as clarify their mode of action by molecular docking. DFT calculations and reactivity indices were calculated for the most potent compounds compared with pyriproxyfen to correlate the relation between chemical structure and reactivity.

## Results

### Chemistry

**Scheme 1:**
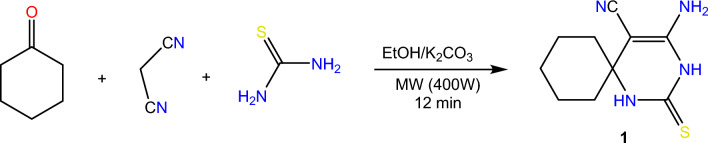
Synthesis of spiro pyrimidine enaminonitrile 1.

A class of organic compounds known as spiro compounds is distinguished by a distinctive chemical structure in which two or more rings share a single common atom. These compounds are important agents in insecticidal chemistry because they possess a wide range of biological activities. Also, pyrimidine derivatives have a wide range of biological activities in various fields. Therefore, this study describes a highly effective approach for the synthesis of a novel series of biologically active spiro pyrimidine derivatives along with an evaluation of their insecticidal potency which has been achieved. Initially, spiro pyrimidine **1** which was prepared in the literature by refluxing cyclohexanone, malononitrile, and thiourea in the presence of sodium methoxide^24^, was prepared in this study under more efficient conditions using microwave irradiation at 400 W led to the formation of compound **1** in high yield and shorter time interval than that reported in the literature. Scheme 1.

**Scheme 2:**
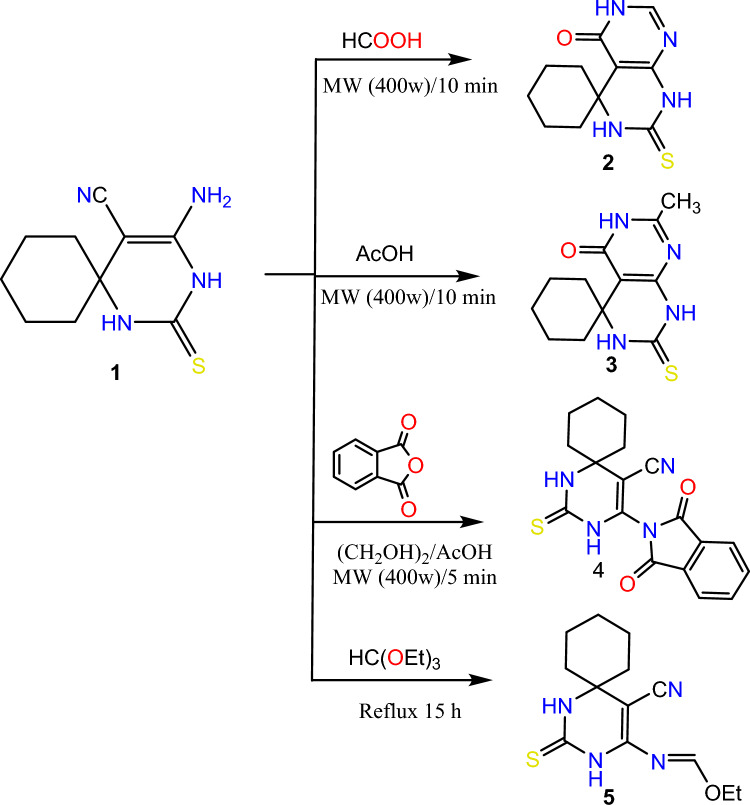
Synthesis of spiro pyrimidine derivatives 2-5.

Due to the numerous reactivity modes of bifunctional aminonitriles which come from the presence of highly nucleophilic center NH_2_, and highly electrophilic center CN, compound **1** was subjected to react with different aliphatic acids such as formic acid and acetic acid using microwave irradiation at 400 W afforded spiro pyrimido[4,5-*d*]pyrimidine derivatives **2**, and **3** by dimurth rearrangement. The chemical structures of compounds **2**, and **3** were approved by spectral data, IR spectra show the disappearance of NH_2_, and CN function groups and the appearance of CO at 1688, and 1685 cm^−1^ for compounds **2**, and **3**, respectively. ^1^H NMR spectra showed the appearance of olefinic CH of pyrimidone of compound **2** as a singlet signal at 7.88 ppm. CH_3_ peak of compound **3** was revealed at 2.61 ppm as a singlet signal. Furthermore, the nucleophilicity of NH_2_ of compound **1** was estimated by reaction with different electrophilic centers such as phthalic anhydride and triethyl orthoformate which led to the formation of spiro pyrimidine derivatives **4**, and **5** respectively. The IR spectra of both compounds showed the disappearance of NH_2_ and the appearance of two values at 1706, and 1793 cm^−1^ corresponding to two CO groups of compound **4**. ^1^H NMR spectrum of compound **4** showed the appearance of aromatic protons of phenyl ring as a multiplet at 7.54–8.08 ppm, and for compound **5**, it showed a triplet peak at 1.08 ppm and quarted at 4.20 ppm corresponding to CH_2_ and CH_3_ of ethoxy group. Scheme 2.

**Scheme 3:**
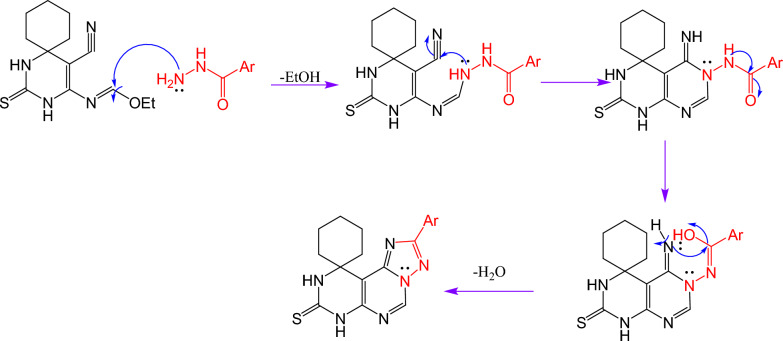
Proposed mechanism for synthesis of compounds 6 and 7.

Cyclocondensation of compound **5** using microwave irradiation at 400 w with different hydrazide derivatives such as benzoyl hydrazide and salicyloyl hydrazide afforded pyrimido[5,4-*e*][1,2,4]triazolo[1,5-*c*]pyrimidine derivatives **6**, **7** which was carried out through the suggested mechanism Scheme 3.

**Scheme 4:**
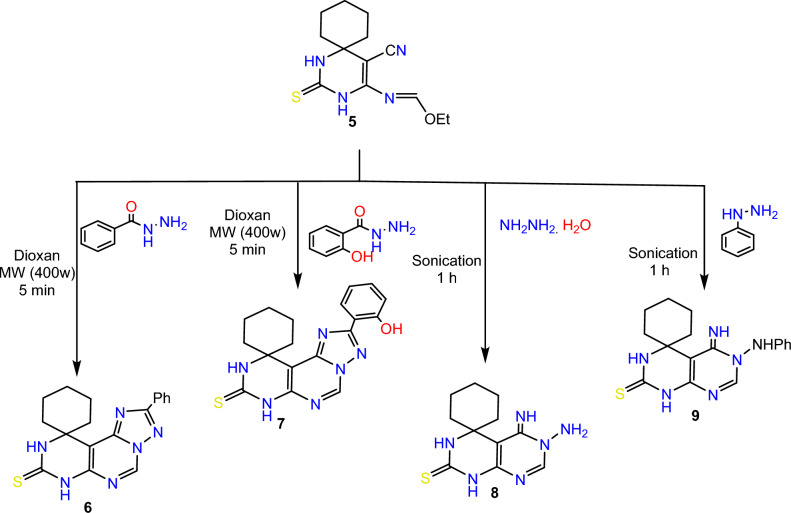
Synthesis of spiro pyrimidine derivatives 6-9.

On the other hand, Sonication of compound **5** with hydrazine hydrate and phenylhydrazine in ethanol led to the formation of pyrimido[4,5-*d*]pyrimidine derivatives **8**, and **9**, respectively. IR spectra of the previous compounds showed the disappearance of CN group, H NMR shows peaks in the region of 7.01–8.01 corresponding to aromatic protons of compounds **6**, **7**, and **9**, also CH olefinic of the pyrimidine ring of compounds 6–9 showed at range of 8.04–8.87 ppm. Scheme 4.

### Biological activity

#### Larvicidal activity

**Figure 1:**
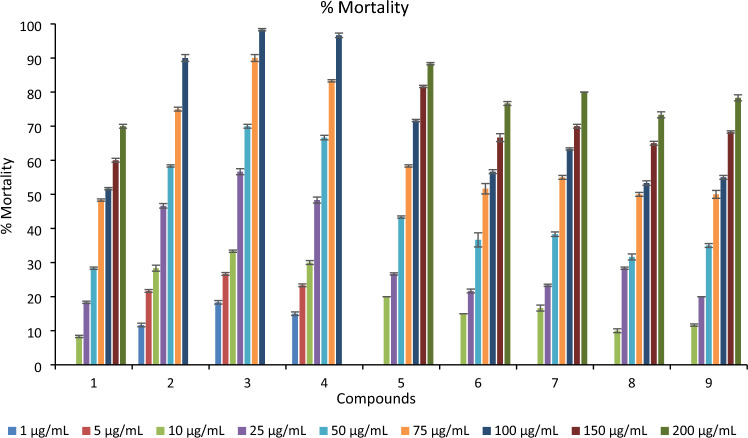
The % Mortality of 3rd larval instar of *Culex pipiens* observed 24 h post-treatment at different concentrations of spiro pyrimidine derivatives.

The toxic effect of the tested compounds was assessed against 3rd instar larvae of *C. pipiens* to record the mortality after 24 h of exposure. The percent mortalities were concentration-dependent and increased gradually with concentration increase as shown in Fig. 1. The probit analysis of the mortality data demonstrated the activity of the synthesized compounds as LC_50_ and slope values, and other statistical parameters Table 1. Data showed that the toxicity of synthesized compounds greatly varied against the *C. pipiens* larvae. The tested compounds were arranged according to their LC_50_ values as follows: **3** > **4** > **2** > **5** > **7** > **6** > **9** > **8** > **1** Table 1. Where, **3**, **4** and **2** were the most potent synthesized compounds with LC_50_ values of 12.43, 16.29 and 21.73 µg/mL, respectively. While, **5** and **7** showed moderate activities with LC_50_ values of 48.21 and 63.14 µg/mL, respectively, then 6 (73.20 µg/mL), 9 (75.73 µg/mL) and 8 (80.50 µg/mL) were less potent, respectively, and **1** was the least active compound with LC_50_ value 95.18 µg/mL. The chi-square values of all tested compounds were significant at *P* < 0.05 and the population showed a homogenous response to treatment with all compounds.

**Table 1:** Toxicity of spiro pyrimidine derivatives against 3rd larval instar of *Culex pipiens* 24 h post-treatment.

Compd. (µg/mL)	LC_25_ (*F.l. at 95%)	LC_50_ (*F.l. at 95%)	LC_90_ (*F.l. at 95%)	**Slope ± SE	****X^*2*^
**1**	33.92 (26.76–40.82)	95.18 (81.52–113.26)	675.90 (465.69–1143.69)	1.50 ± 0.13	2.63
**2**	5.64 (2.16–8.45)	21.73 (11.98–39.29)	282.15 (200.07–1122.11)	1.15 ± 0.09	5.20
**3**	3.57 (0.76—4.94)	12.43 (4.65—25.05)	132.91 (105.66–750.36)	1.24 ± 0.09	7.27
**4**	4.64 (1.16–6.64)	16.29 (6.98–33.23)	177.15 (144.18–990.38)	1.23 ± 0.09	6.23
**5**	18.65 (14.19–23.04)	48.21 (41.26–55.63)	292.76 (292.76–409.51)	1.63 ± 0.13	6.07
**6**	24.31 (18.23–30.22)	73.20 (62.26–86.60)	594.40 (410.76–1001.09)	1.40 ± 0.13	3.72
**7**	21.78 (16.30–27.13)	63.14 (53.82–73.95)	477.12 (342.40–755.47)	1.45 ± 0.13	5.10
**8**	26.57 (20.09–32.84)	80.50 (68.43–95.83)	661.67 (449.60–1145.88)	1.40 ± 0.13	3.68
**9**	28.12 (22.01–34.03)	75.73 (65/36–88.25)	497.36 (361.14–772.37)	1.56 ± 0.13	3.52
Pyriproxyfen	0.06 (0.04 -0.08)	0.18 (0.14 -0.21)	1.31 (0.94—2.07)	1.48 ± 0.13	1.42

* (F.l.) Fiducially limits.

**Slope of the concentration-inhibition regression line ± standard error.

***X^*2*^ chi-square significant at *P* < 0.05.

#### Biological effects

Treatment of early 3rd instar larvae of *C. pipiens* with different concentrations of **2**, **3**, **4** and pyriproxyfen exhibited remarkable effects on larval and pupal durations, developmental rates and growth rates as shown in Table 2. The results demonstrated a significant prolongation of the larval and pupal durations, in a concentration-dependent manner. Pyriproxyfen greatly increased the larval and pupal durations at concentrations (0.05, 0.1, 0.25, 0.5 and 1 µg/mL). While, compounds **3** and **2** were highly prolonged larval duration relative to **4** at the concentrations (1, 5, 10, 25, 50, 75 and 100 µg/mL). At 75 and 100 µg/mL, compound **3** increased the larval durations to 7.33 and 8.00 days in comparison to 4.33 days in control and prolonged the pupal duration to 4.00 and 4.67 days relative to 1.33 days in control. While, pyriproxyfen increased the larval and pupal durations to 8.67 and 5.00 days, respectively at 1 µg/mL in comparison to the control. It was noticed that for all tested compounds the percent pupation and adult emergency had an inverse relationship with tested concentrations and gradually decreased with concentration elevation. At 100 µg/mL, the pupation decreased to 1.67, 3.34 and 10.00% after treatment with compounds **3**, **4** and **2,** respectively. Whereas, pyriproxyfen decreased the pupation to 11.67% at 1 µg/mL. Also, the adult emergency and growth index were completely inhibited by treatment with **3** and **4** at 100 µg/mL, while **2** decreased the adult emergency to 33.33% and growth index to 4.54. Pyriproxyfen decreased the adult emergency to 46.66 and 28.57% at 0.5 and 1 µg/mL, respectively. Regarding adult longevity, treatment with pyriproxyfen resulted in a remarkable prolonged to 8.33 and 9.33 days at 0.5 and 1 µg/mL relative to 5.34 days in control. After treatment with **3**, the adult longevity durations were significantly prolonged to 8.00 and 8.33 days at 50 and 100 µg/mL. While, compound **2** showed less effect on adult longevity durations than **4** and **3**, respectively.

**Table 2:** Effect of **2**, **3**, **4**, and pyriproxyfen on some biological aspects after treatment of early third instar larvae of *Culex pipiens.*

Conc. (µg/mL)	Larval mortality %	Mean larval duration (days) ± SD	Pupation %	Mean pupal duration (days) ± SD	Adult emergence % (a)	Adult longevity (days) (b) ± SD	Growth index (a/b)
Untreated	1.66	4.33 ± 0.57^a^	96.61	1.33 ± 0.58^a^	98.24	5.34 ± 0.57^a^	18.39
2							
1	11.67 ± 0.57	4.67 ± 1.73^b^	88.33	1.33 ± 1.53^a^	90.56	5.67 ± 1.15^a^	15.97
5	21.66 ± 0.33	4.67 ± 1.53^b^	78.33	1.66 ± 1.73^b^	85.10	6.00 ± 1.73^b^	14.18
10	28.33 ± 0.88	5.00 ± 1.73^c^	71.67	2.00 ± 1.73^b^	79.07	6. 33 ± 1.15^c^	12.49
25	46.66 ± 0.66	5.33 ± 2.31^d^	53.34	2.33 ± 1.53^c^	62.50	6.33 ± 0.58^c^	9.87
50	58.33 ± 0.33	5.66 ± 2.08^e^	41.33	2.33 ± 1.15^c^	60.00	6.66 ± 1.53^d^	9.00
75	75.00 ± 0.57	6.00 ± 1.73^f^	25.00	2.67 ± 0.58^d^	53.33	7.33 ± 1.15^e^	7.27
100	90.00 ± 1.00	6.67 ± 1.15^ g^	10.00	3.33 ± 1.53^e^	33.33	7.33 ± 0.58^e^	4.54
3							
1	18.33 ± 0.33	5.33 ± 1.53^b^	81.67	2.00 ± 1.53^b^	83.67	6.33 ± 0.57^b^	13.21
5	26.66 ± 0.88	5.67 ± 2.31^c^	73.33	2.33 ± 1.00^c^	70.45	6.67 ± 1.15^c^	10.56
10	33.33 ± 0.33	5.67 ± 1.15^c^	66.67	2.67 ± 0.57^d^	62.50	6.67 ± 1.52^c^	9.37
25	56.66 ± 0.88	6.00 ± 1.00^d^	43.34	3.33 ± 1.53^e^	57.69	7.00 ± 1.73^d^	8.24
50	70.00 ± 0.57	6.33 ± 0.58^d^	30.00	3.33 ± 0.58^e^	44.44	7.33 ± 1.52^d^	6.06
75	90.00 ± 1.00	7.33 ± 0.58^e^	10.00	4.00 ± 1.73^f^	16.66	8.00 ± 1.00^e^	2.00
100	98.33 ± 0.33	8.00 ± 1.73^f^	1.67	4.67 ± 0.58^ g^	00.0	8.33 ± 0.58^f^	00
4							
1	15.00 ± 1.00	4.67 ± 1.73^b^	85.00	1.67 ± 0.58^b^	86.27	6.00 ± 1.00^b^	14.37
5	23.33 ± 0.66	5.33 ± 1.73^c^	76.66	2.00 ± 1.00^c^	78.26	6.33 ± 1.73^c^	12.36
10	30.00 ± 0.57	5.33 ± 0.58^c^	70.00	2.67 ± 1.53^d^	73.81	6.33 ± 0.58^c^	11.66
25	48.33 ± 0.88	5.67 ± 1.15^d^	51.67	3.00 ± 1.73^e^	58.06	6.67 ± 1.15^d^	8.70
50	66.66 ± 0.66	6.00 ± 1.00^e^	33.34	3.33 ± 1.53^e^	45.00	6.67 ± 0.58^d^	6.75
75	83.33 ± 0.33	6.33 ± 0.58^f^	16.67	3.67 ± 1.52^f^	20.00	7.33 ± 0.58^e^	2.72
100	96.66 ± 0.66	7.67 ± 1.15^ g^	3.34	4.33 ± 0.58^g^	00.00	8.00 ± 0.00^f^	00
Pyriproxyfen							
0.05	21.66 ± 1.00	6.34 ± 0.58^b^	78.66	2.67 ± 0.58^b^	82.97	6.66 ± 1.15^b^	12.48
0.1	36.66 ± 0.57	6.67 ± 1.15^c^	63.34	3.00 ± 1.00^c^	78.94	7.00 ± 0.00^c^	11.28
0.25	53.55 ± 0.33	7.33 ± 0.58^d^	46.45	3.67 ± 1.15^d^	60.71	7.67 ± 1.53^d^	7.91
0.5	75.00 ± 1.15	8.00 ± 1.00^e^	25.00	4.67 ± 1.53^e^	46.66	8.33 ± 0.58^e^	5.60
1	88.33 ± 0.66	8.67 ± 2.08^f^	11.67	5.00 ± 1.73^f^	28.57	9.33 ± 2.08^f^	3.06

Means with the same letters are not significantly different.

Each value represents the mean of three replicates ± SD Standard deviation.

#### Morphological malformation

**Figure 2:**
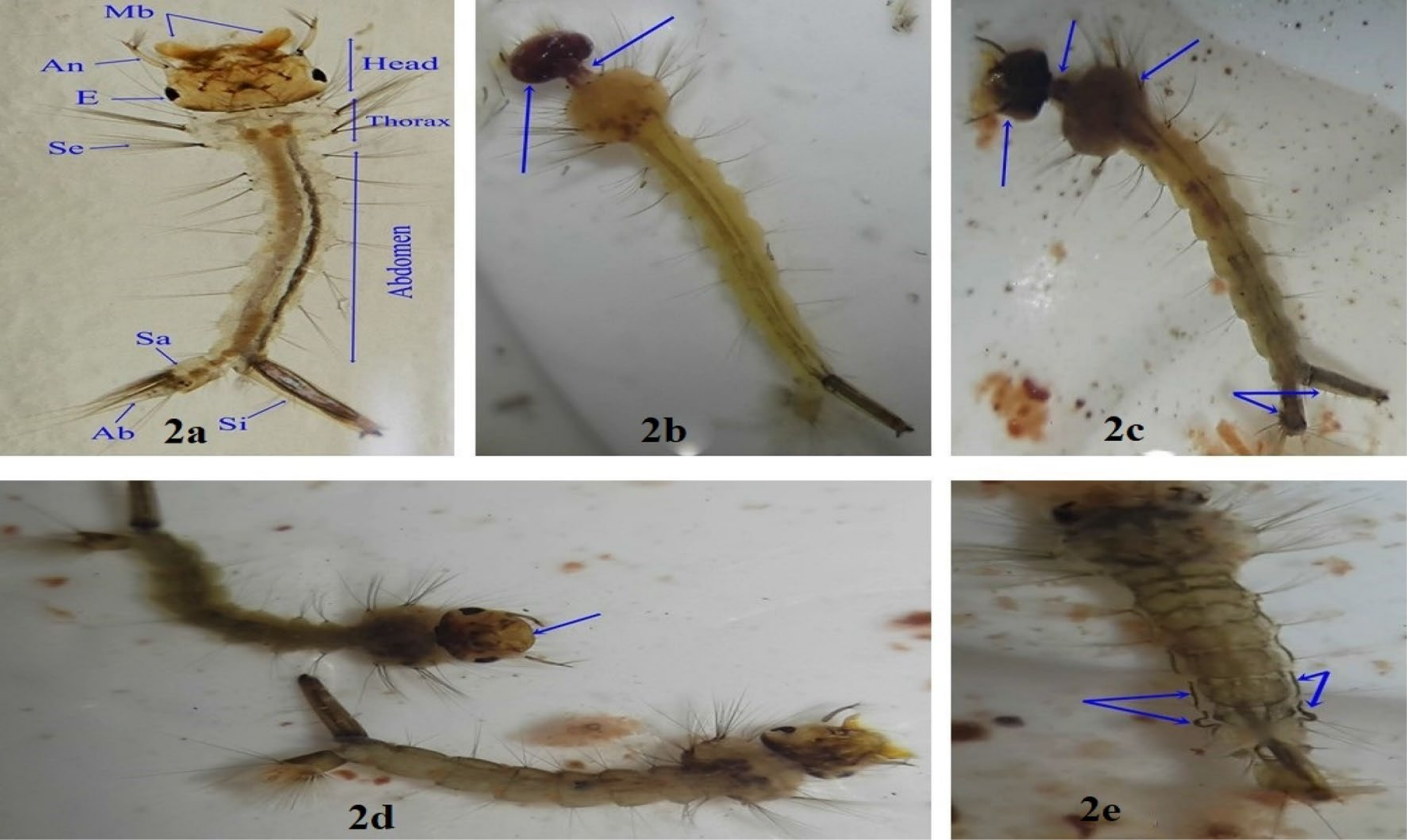
(**a**) Normal larva of *Culex pipiens* is pale brown in colour with well-developed body parts such as a head with mouth brushes (Mb), Antennae (An) and eye (E) also, thorax with setae (Se), abdomen with a siphon (Si), saddle (Sa) and anal brushes (Ab). Abnormalities were observed in treated larvae with spiro pyrimidine derivatives. (**b**) Treated larvae with elongated neck and darkened head. (**c**) Larvae with melanized head and thorax. (**d**) Highly pigmented larvae with lost mouth brushes. (**e**) Dorsal view of larval abdomen with attached old cuticle.

**Figure 3:**
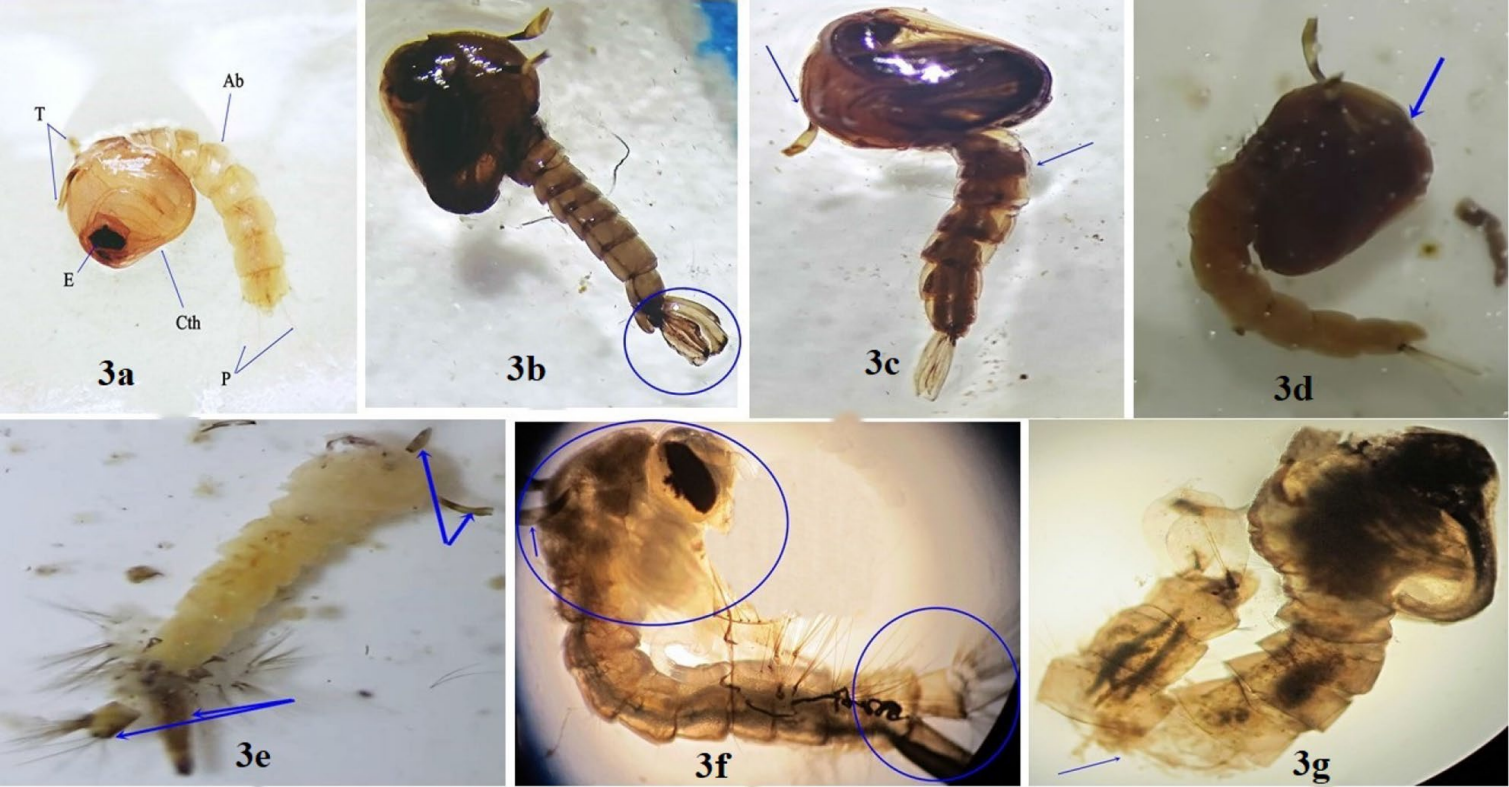
(**a**) Normal faint brown comma-shaped pupa of *Culex pipiens* showing well-defined cephalothorax (Cth) with pair of respiratory trumpets (Rt) and eye (E), and segmented abdomen (Ab) ended with a pair of paddles (P). Pupal morphological and developmental malformations detected after spiro pyrimidine derivatives treatment (**b**) darkened pupa with malformed paddles, (**c**) highly cutinized, loss of a trumpet and inverted abdomen, (**d**) highly darkened cephalothorax. Developmental malformations (**e**) pale yellow larval-pupal intermediate with pair of trumpets and ended with larval organs, (**f**) larval-pupal intermediate with destructed cephalothorax with the appearance of adult head and presence of larval abdomen (**g**) adult- pupal intermediate with the destructed pupal abdomen.

**Figure 4:**
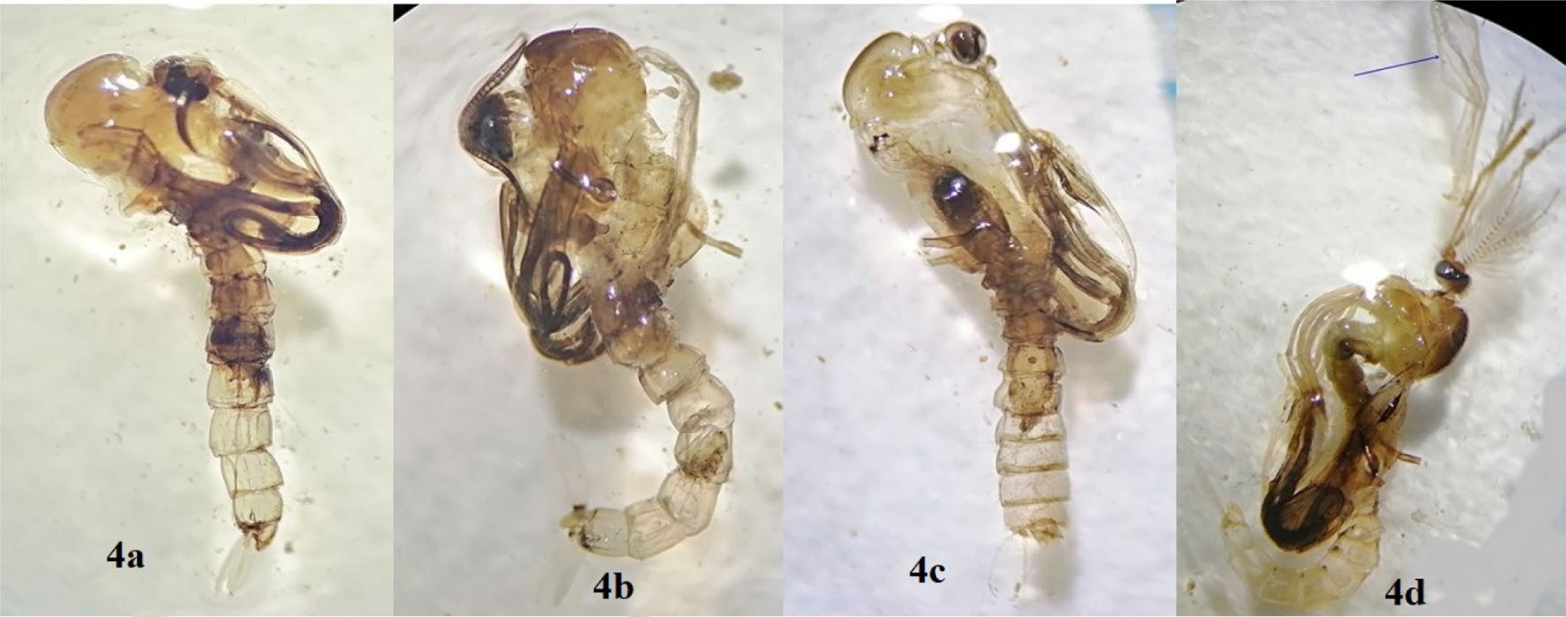
Abnormalities in adult and adult emergency due to treatment with spiro pyrimidine derivatives, (**a**) adult-pupal intermediate with an attached proboscis, antennae, wings, legs and end of abdomen to the pupal exuvia. (**b**,**c**) Incomplete ecdysis adult and the adult appendages cuffed in the pupal exuviae. (**d**) Failure in adult emergency and malformation in the emerged wing.

In addition to disruption of developmental durations and rates, many morphological malformations in the developmental stages and adult emergency were observed after treatment with spiro pyrimidine derivatives Figs. 2, 3, and 4. The normal larva of *C. pipiens* is pale brown with well-defined body parts as shown in Fig. 2a. The treatments with spiro pyrimidine derivatives showed elongated neck and darkened head Fig. 2b. Also, a larva with a melanized head and thorax was detected in Fig. 2c. The pigmentation extended to the whole body of larvae (dark brown to grey colour) with loss of mouth brushes Fig. 2d. A dorsal view of the larval abdomen showed old cuticle remained attached and highly melanized Fig. 2e. *C. pipiens* pupae have a faint brown comma-like shape showing a well-developed cephalothorax with pair of respiratory trumpets and the abdomen is curled beneath with a pair of paddles Fig. 3a. The treatment with spiro pyrimidine derivatives showed severe pupal morphological and developmental malformations as the darkened pupa with malformed paddles Fig. 3b. Highly cutinized, loss of a trumpet and inverted abdomen were detected in Fig. 3c, also highly darkened cephalothorax was observed in Fig. 3d. Many developmental malformations were investigated as pale yellow larval-pupal intermediate with a pair of trumpets and ended with larval abdomen ending bearing siphon and saddle Fig. 3e. Also, larval-pupal intermediate with destructed cephalothorax with the appearance of adult head and presence of larval abdomen Fig. 3f, in addition, adult-pupal intermediate with the appearance of adult head and destructed pupal abdomen Fig. 3g. The treatment with spiro pyrimidine derivatives also showed abnormalities in adults and failure in a normal adult emergency. Adult-pupal intermediate with an attached proboscis, antennae, wings, legs and end of abdomen to the pupal exuviae only adult head and humped thorax emerged Fig. 4a. Another feature of the developmental disruption was incomplete ecdysis adult and the adult appendages cuffed in the old pupal exuvia Fig. 4b,c. Failure in an adult emergency due to the legs, wings and end of abdomen still attached to pupal exuvia beside the emerged wing was deformed Fig. 4d.

#### Cytotoxicity against normal human cell line

Cytotoxicity assay was performed to evaluate the safety of the most effective compounds 2, 3 and 4 against normal human lung fibroblast (WI-38). The normal cell line was treated with (100, 50, 25, 12.5, 6.25, 3.12 and 1.56 µM) of the tested compounds depending upon the concentration used in *C. pipiens* larvicidal activity. Compounds 2 and 4 exhibited weak cytotoxicity of IC_50_ 81.20 ± 4.1 and 58.74 ± 3.0 µM**,** respectively Table 3. On the other hand, compound 3 was noncytotoxic with IC_50_ 105.20 ± 2.7 µM and considered safe against normal human cell lines. These results confirmed the safety of the synthesized compounds in humans as non-target organisms. In addition, this safety approved the target of research to use selective chitinase inhibitors against insects without any adverse effects on non-targets**.**

**Table 3:** Cytotoxic activity of 2, 3 and 4 against normal human lung fibroblast (WI-38).

Compound	In vitro cytotoxicity IC_50_ (µM)*
	WI38
**2**	81.20 ± 4.1
**3**	105.20 ± 2.7
**4**	58.74 ± 3.0

*IC50 (µM) : 1–10 (very strong). 11–20 (strong). 21–50 (moderate). 51–100 (weak) and above 100 (non-cytotoxic).

### In silico studies

#### Molecular docking study

**Figure 5:**
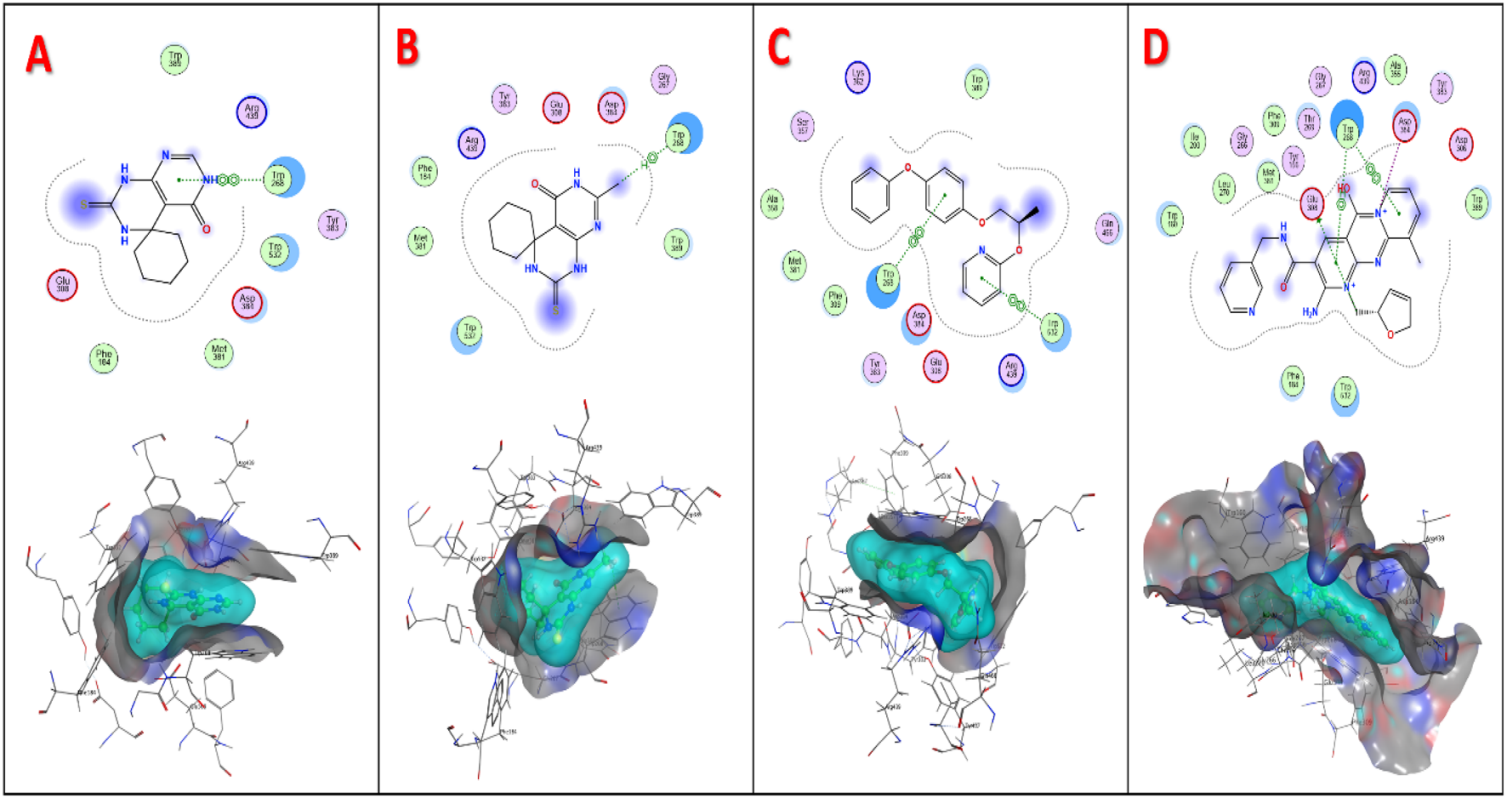
(**A**–**D**) 2D and 3D interaction visualization of compounds (**A**) **2**, (**B**)** 3, (C)** pyriproxyfen, and **(D)** Co crystalized ligand (2-8-S2), respectively with the active site of (*Ostrinia furnacalis* chitinase h) (PDB ID: 6 JMN).

The molecular docking was carried out to study the binding affinity of designed compounds, pyriproxyfen and co-crystalized ligand (2-8-S2). The binding modes of the tested compounds against the prospective target enzyme (*Ostrinia furnacalis* chitinase h) (PDB ID: 6 JMN) were investigated to compare their potencies. The interaction binding energies of the docked compounds are illustrated in Table 4. The docked ligand showed binding with Glu308, Asp384 and Trp268 of *Of* Chi- h binding site through different binding interactions, with interaction energy of − 9.33 kcal/mol, and its binding mode was demonstrated in Table 4. Pyriproxyfen exhibited 2 pi-pi interactions with Trp268 and Trp532 binding energy − 6.73 kcal/mol. The results of molecular docking revealed that some of the docked spiro pyrimidine derivatives had similar binding modes as **2**, **3**, **4**, **7**, **8,** and **9**, which formed pi-pi or pi-h interactions with Trp268 of binding site as shown in Table 5. While, compounds **1** and **5** formed hydrogen bonding with Met381, and **6** showed different binding interactions with Trp532 (H-pi) and Phe 309(pi-H). The interaction energies of the spiro pyrimidine derivatives ranged from − 5.55 kcal/mol which was achieved by compound **1** to − 7.27 kcal/mol for **7** as demonstrated in Table 4. 2d and 3d visualization for ligand interaction of compounds **2**, **3**, pyriproxyfen, and co-crystallized ligand with the active pocket of (*Of* chitinase h) were illustrated in Fig. 5. All figures for all compounds were added to the supporting information file.

**Table 4:** Docking score and RMSD for compounds (**1**–**9**), pyriproxyfen and Co crystalized ligand.

Compound	Docking score	
	(Kcal/mol)	RMSD
**1**	− 5.55	1.34
**2**	− 5.63	1.55
**3**	− 6.11	1.49
**4**	− 7.02	1.45
**5**	− 6.54	1.85
**6**	− 7.27	0.82
**7**	− 7.16	1.3
**8**	− 6.12	1.10
**9**	− 6.89	1.41
**Pyriproxyfen**	− 6.73	1.54
**Co crystalized (2–8-S2)**	− 9.33	1.74

**Table 5:** Ligand interaction of compounds (**1**–**9**) and pyriproxyfen with (*Ostrinia furnacalis* chitinase h) enzyme.

Compound	Ligand	Receptor	Type of interaction	Interaction distance °A	E(Kcal/mol)
**1**	N 12	SD MET 381	H-donor	3.76	− 1.8
**2**	6-ring	6-ring TRP 268	pi-pi	3.91	− 0.0
**3**	C 8	6-ring TRP 268	H-pi	3.86	− 0.7
**4**	S 1	NE1 TRP 532	H-acceptor	3.79	− 1.7
	C 27	6-ring TRP 532	H-pi	4.26	− 0.8
	C 39	5-ring TRP 532	H-pi	4.05	− 0.8
	C 39	6-ring TRP 532	H-pi	4.46	− 0.6
	5-ring	5-ring TRP 268	pi-pi	3.63	− 0.0
	6-ring	5-ring TRP 268	pi-pi	3.97	− 0.0
**5**	N 20	SD MET 381	H-donor	3.72	− 1.9
**6**	C 38	5-ring TRP 532	H-pi	3.78	− 0.5
	6-ring	CZ PHE 309	pi-H	3.65	− 0.7
**7**	C 30	5-ring TRP 532	H-pi	3.90	− 1.0
	6-ring	CZ PHE 309	pi-H	3.82	− 0.9
	6-ring	CB ASP 384	pi-H	3.51	− 0.5
	5-ring	6-ring TRP 268	pi-pi	3.89	− 0.0
	6-ring	5-ring TRP 268	pi-pi	3.87	− 0.0
**8**	S 1	NE1 TRP 532	H-acceptor	3.39	− 0.8
	N 15	N TRP 268	H-acceptor	3.09	− 0.5
	6-ring	N TRP 268	pi-H	4.82	− 0.6
**9**	6-ring	6-ring TRP 268	pi-pi	3.98	− 0.0
**Pyriproxyfen**	6-ring	6-ring TRP 268	pi-pi	3.71	− 0.0
	6-ring	5-ring TRP 532	pi-pi	3.57	− 0.0
**Co Crystalized (2–8-S2)**	C19 45	OE1 GLU 308	H-donor	3.31	− 1.8
	N 30	OD2 ASP 384	Ionic	3.54	− 1.7
	6-ring	CB TRP 268	pi-H	3.54	− 0.8
	6-ring	6-ring TRP 268	pi-pi	3.96	− 0.0

#### DFT calculations

**Figure 6:**
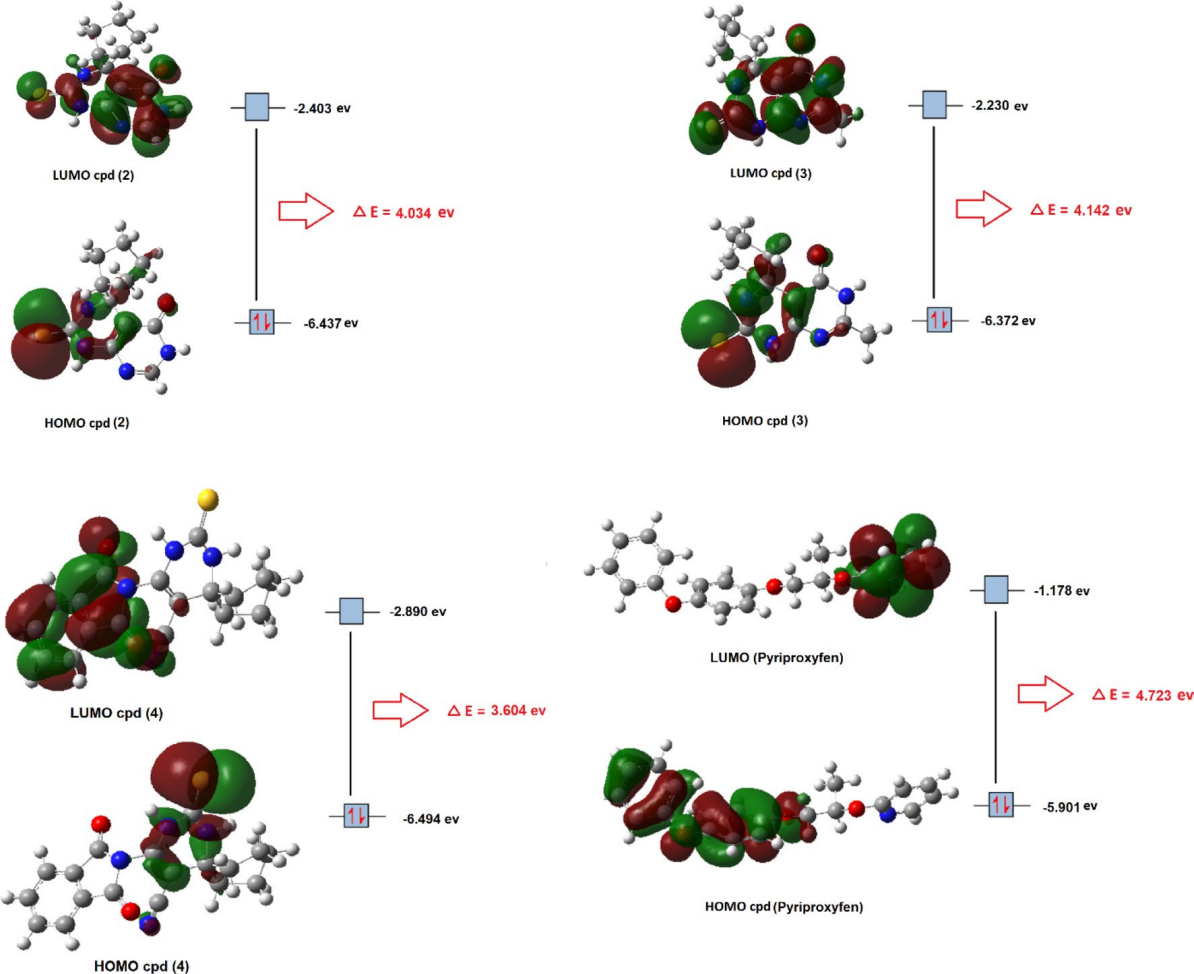
Frontier molecular orbitals (HOMO and LUMO) of compounds (**2**–**4**) and pyriproxyfen.

Densty functional theory (DFT) was carried out to correlate the relation between reactivity and chemical structure using gaussian 09 software^25^, target compounds were computed at the B3LYP/6–31 + G (d, P) level of theory to calculate the frontier molecular orbitals HOMO and LUMO energy Fig. 6^26^. Reactivity indices of the most potent compounds (**2**–**4**) and pyriproxyfen were calculated from Frontier molecular orbitals energies (E_HOMO_ and E_LUMO_) by applying DFT calculations according to the following equations Table 6

1 $${\text{Eenergy}}\;{\text{Gap}}\;\left( {{\text{E}}_{{{\text{Gap}}}} } \right) = {\text{E}}_{{{\text{HOMO}}}} - {\text{E}}_{{{\text{LUMO}}}}$$

2 $${\text{Ionization}}\;{\text{potential }}\left( {\text{I}} \right) = - {\text{E}}_{{{\text{HOMO}}}}$$

3 $${\text{Electron}}\;{\text{Affinity}}\;\left( {\text{A}} \right) = - {\text{E}}_{{{\text{LUMO}}}}$$

4 $${\text{Electronegativity}}\;(\chi ) = \left( {{\text{I}} + {\text{A}}} \right)/{2}$$

5 $${\text{Chemical}}\;{\text{potential}}\;\left( {\text{P}} \right) = - \, \chi$$

6 $${\text{Chemical}}\;{\text{Hardness}}\;\left( \eta \right) = \left( {{\text{I}} - {\text{A}}} \right)/{2}$$

7 $${\text{Chemical}}\;{\text{Softness}}\;\left( {\text{S}} \right) = {1}/{2}\eta$$

**Table 6:** Reactivity indices based on frontier orbitals of compounds (**2**–**4**) and pyriproxyfen.

Compound	E_HOMO_ (eV)	E_LUMO_ (eV)	E_Gap_	I	A	χ	p	η	S
**2**	− 6.437	− 2.403	4.034	6.437	2.403	4.420	− 4.420	2.017	0.248
**3**	− 6.372	− 2.230	4.142	6.372	2.230	4.301	− 4.301	2.071	0.241
**4**	− 6.494	− 2.890	3.604	6.494	2.890	4.692	− 4.692	1.802	0.277
**Pyriproxyfen**	− 5.901	− 1.178	4.723	5.901	1.178	3.539	− 3.539	2.362	0.212

The chemical stability of a compound is determined by the energy difference between HOMO and LUMO; the compound with the smaller (E_Gap_) value is more stable than the other compounds. According to (E_Gap_) values, compound **4** has greater chemical stability than compounds **2**, **3**, and pyriproxyfen, respectively. Additionally, the difference in energy between HOMO and LUMO reflects the chemical hardness and softness of the molecule; the higher energy gap suggests a molecule that is less polarized (hard), while the smaller energy gap indicates a molecule that is more polarized (soft). From (η) values it was shown that Pyriproxyfen > **3** > **2** > **4** in chemical hardness. On the other hand, From (S) values it was shown that **4** > **2** > **3** > pyriproxyfen in chemical softness. Ionization potential (I) reflects the donating properties of the molecule, a compound that has the lower value of (I) easily donates its electron (easily oxidized). From ionization potential values it was shown that pyriproxyfen is the most probable one to donate its electrons and act as a reducing agent. Chemical potential (P) and electronegativity (χ) are related to each other, as chemical potential increase and electronegativity decrease the ability of a compound to donate electrons increases. From (P) and (χ) it was shown that pyriproxyfen is the less electronegative compound and more donating one than **3**, **2** and **4**, respectively.

## Discussion

Although, the drawbacks of the continuous application of synthetic insecticides, there is a continuing demand for novel chemicals that can meet the need for the development of innovative solutions for managing pest insects^27^. Pyrimidine ring has drawn strong attention because it has been widely used in insecticides^1^**.** Pyrimidine derivatives exhibit insecticidal activity against rice planthopper nymphs at the moulting time by using rice seedling assay (0.03–30 mg/L). There were attempts to modify the substituents on the pyrimidine ring to enhance the insecticidal activity and discover new compounds. These compounds are known as insect growth regulators (IGR) and showed high residual insecticidal efficacy at 100 mg/L, with low adverse effects on honeybees and natural enemies^28^. The trifluoromethyl pyrimidine derivatives indicated moderate insecticidal activities against *Mythimna separata* and *Spdoptera frugiperda* exposed by leaf-dip method at 500 μg/ml^29^. Tetrahydropyrimidine exhibited moderate to high stomach toxicity against the second larval instar of *Spodoptera litura* depending on the exposure time and using (31.4- 500 ppm) concentrations^30^. Ismail et al.^31^ interpreted that the thiadiazolopyrimidine possesses high insecticidal activity toward *Spodoptera litura* after exposure by leaf dipping technique to (250- 1250 ppm), the presence of this moiety stabilizes the strong aromaticity of the five-membered ring system, which greatly improves in vivo stability and diminishes toxicity for higher vertebrates. The 4-dimethylaminopyridinium showed weak to moderate residual potency against *C. pipiens* at concentrations (0.0156 and 0.0312 g/L), for 5–10 days^32^. Overall, the synthesized spiro pyrimidine derivatives were highly effective against *C. pipiens* relative to previously mentioned by Alahmadi et al.^32^, where 2, 3 and 4 exhibited LC_50_ values of 12.43, 16.29 and 21.73 µg/mL, respectively, 24 h post-treatment.

In addition, the spiro compounds are crucial steps in the development and design of new pesticides. These compounds with insecticidal, antiviral, fungicidal, bactericidal, herbicidal and plant growth regulators^20^. Therefore, we became interested in pyrimidine rings and started studying to create novel chemicals containing these rings.

Pyrimidine derivatives didn’t show acute toxicity as most of the traditional chemical insecticides because they didn’t kill the insect directly but they acted as growth inhibitors. The development of larvae was affected and retarded also, the morphology and emergency of adults were affected^40^. Therefore, the biological impacts of these pyrimidine derivatives were conducted to characterize their latent effects. Pyriproxyfen is a pyridine-based insecticide and is considered a juvenile hormone analoge that affects insect moulting^41^. Pyriproxyfen has been reported to be safe for the ecosystem and less toxic to bees, natural enemies and mammals^42^. Pyriproxyfen showed strong efficacy against susceptible strains of *Ae. aegypti* larvae. Unlike conventional larvicides, pyriproxyfen greatly inhibits adult emergence although, it exhibits weak larval mortality^43,44^. Similar results were obtained from the application of **3**, **2** and **4** to *C. pipiens* larvae, where the tested compounds showed some remarkable insecticidal effects and great biological impacts on larval development. Besides the high activity of **3** and **4** against mosquito larvae, they caused complete adult emergence inhibition at 100 µg/mL. Compounds **2**, **3** and **4** not only disturbed the development and growth of *C. pipiens* larvae but also, several morphological aberrations were detected. So, the latent mortality occurs by inhibition of their development or by their failure to complete their normal ecdysis^45^.

Mosquito larvae grow through different instars and stages in the whole life cycle associated with the shedding of the old outer exoskeleton and the formation of a new larger one. This process is known as ecdysis or moulting^46^. Pyrimidine derivatives (BVDU) block this process so, the insects cannot moult properly, and this is in harmony with our results. The larval and pupal durations were significantly prolonged, retardation of growth and moulting disturbances and/or deformities^40^. Hamaidia and Soltani^45^ reported that severe morphological aberrations were detected in 4th instar larvae of *C. pipiens* treated by IGRs, thus they were unable to dive up to the surface to breathe. Moreover, some pupae at the larval-pupal intermediate were unable to escape from the larval exuvia and other pupae had only one trumpet. Also, the adults could not emerge because their legs and wings were attached to old exuvium resulting in adult death. The treatment of *Spodoptera frugiperda* larvae with pyrimidine nucleoside analogue influenced several developmental aberrations as larval–pupal intermediates with a partial pupal cuticle, incomplete sclerotized pupae and the moths were not able to emerge and remove their pupal skin^40^. Moreover, the ultrastructural examination of the cuticle of the 4th larval instar of *C. pipiens* treated with pyriproxyfen revealed a bulk cuticle with procuticle lamellae and multilayered cuticle overlays epidermal cells^47^ which, interpreted the darkening of treated larvae and pupae. Overall, the pyrimidine derivatives unlike conventional insecticides, caused cumulative mortalities through the development of larval, pupae and adults. So, these compounds act by dual effect as a larvicidal agent and JHA, therefore decreasing the rate of resistance in insects and considered a building block for many other derivatives with high toxicity.

### Structure–activity relationship study (SAR)

Nine compounds were synthesized by insertion of different moieties. It was noted that compounds **3**, **4** and **2** were the most potent respectively, while **1**, **8** and **9** were the least active compounds. The structure–activity relationship study showed that the insertion of a fused aromatic ring improved the activity of synthesized compounds activity with different ratios. Cyclocondensation of spiro pyrimidine **1** to fused pyrimidopyrimidine which contains amide C=O that enhances the activity of **2** and **3**. On the other hand, pyridopyrimidine which contains C=NH instead of C=O was less potent as observed in compounds **8** and **9**. In addition, the pyrimidinone ring in **2** and **3** are considered aromatic rings while the iminopyrimidine ring in **8** and **9** are not aromatic. The function group C=O is a stronger attracting group than C=NH which causes electronic resonance with the attached NH group and facilitates the aromatization of pyrimidine by conversion to enol form, the hetero atom (N) in aromatic pyrimidine acts as attracting group which leads to increased the potency, this is similar to that detected by Xu et al.^33^ who reported that electron-withdrawing groups inserted at 2- and 4-positions of the aromatic ring could improve insecticidal activity. It was suggested that the benzene plane forms a special face-to-edge aromatic interaction with the amino acids of a target site. These aromatic moieties increase the hydrophobicity of compounds, which enhances their permeability through the insect integument, thereby increasing insecticidal activity^34^. The substitution of aromatic carboxamide moieties resulted in higher inhibition of *Ostrinia furnacalis* chitinase h (*Of* Chi-h) in contrast to the insertion of non-aromatic moieties. These aromatic substituents are conserved in *Of* Chi-h, hydrophobic (π–π) stacking interactions which seem to be critical for activity, while compounds lacking these aromatic groups were inactive^35^. Moreover, when pyrimidine fused with triazole ring as in **6** and **7** led to moderate potency and activities depending on the substituents inserted in triazole ring. The phenyl ring substituted with the donating group as in **7** led to more activity than when triazolo pyrimidine was substituted with phenyl ring as in **6**. The insertion of triazole moieties into the pyrimidine interacts with their target protein and improves their inhibition activity and potency^36^. Finally when the NH_2_ group in compound **1** was substituted with a strong electron-withdrawing group like C=O as in compound **4** also fused with aromatic ring (phenyl) led to potency improvement^37–39^.

## Experimental

### Chemistry

The uncorrected melting points of the substances were determined using Gallen Kamp melting point apparatus manufactured by Sanyo Gallen Kamp in the UK. Microwave reactions were carried out with a Microsynth MA143 instrument, specifically designed for microwave reactions. Ultrasound-assisted reactions were performed using a Digital Ultrasonic Cleaner CD-4830 operating at a frequency of 35 kHz and power of 310 W. Infrared (IR) spectra were recorded on a Pye-Unicam SP-3–300 infrared spectrophotometer using KBr disks, and the results were expressed in wave numbers (cm^−1^). 1H NMR spectra were acquired at different frequencies: 300 MHz on a Varian Mercury VX-300 spectrometer and 400 MHz on a Bruker Avance III NMR spectrometer and ^13^C NMR spectra at 100 MHz. Deuterated dimethylsulphoxide (DMSO-d6) was used as the solvent with tetramethylsilane (TMS) serving as the internal standard. Chemical shifts (δ) were reported in parts per million (ppm). The abbreviations used for signal types were s (singlet), d (doublet), and m (multiplet). All coupling constant (J) values were provided in hertz. Elemental analyses were conducted using a CHN analyzer, and the measured values for all compounds were within ± 0.4 of the theoretical values. The progress of the reactions was monitored using thin-layer chromatography (TLC) on plates coated with UV fluorescent silica gel Merck 60 F254. Visualization of the TLC plates was achieved by exposing them to a UV lamp under different solvent systems as mobile phases. All reagents and solvents were purified and dried using standard methods.

#### 4-amino-2-thioxo-1,3-diazaspiro[5.5]undec-4-ene-5-carbonitrile (1)

A mixture of cyclohexanone (0.98 gm; 0.01 mol), thiourea (0.76 gm; 0.01 mol), malononitrile (0.66 gm; 0.01 mol) and potassium carbonate (1.38 gm; 0.01 mol) in absolute ethanol was irradiated under microwave irradiation at 400W for 12 min. The reaction mixture was poured onto crushed ice and acidified using diluted HCl. The formed solid was filtered off, dried, and recrystallized to afford compound **1*.***

Yield 95%; pale yellow crystal; mp 245 °C (EtOH); IR (cm^−1^) ν: 3333 (NH2), 3219(NH), 2175 (CN). ^1^H-NMR: 1.28–1.71(m, 10H, Cyclohexane), 6.03 (s, 2H. NH2), 9.00 (s, 1H, NH), 9.90 (s, 1H, NH); ^13^C-NMR (100 MHz, DMSO-*d6*) δ (ppm): 20.53 (2), 24.26, 37.62 (2), 54.31, 59.73, 119.81, 149.78, 174.37;. Anal. Calcd for C_10_H_14_N_4_S: C, 54.03; H, 6.35; N, 25.20; Found: C, 54.12; H, 6.33; N, 25.30.

#### 2'-thioxo-2',3'-dihydro-1'H-spiro[cyclohexane-1,4'-pyrimido[4,5-d]pyrimidin]-5'(6'H)-one (2)

A mixture of compound **1** (2.22 gm; 0.01 mol) and 15 ml formic acid was irradiated under microwave irradiation at 400W for 10 min. The solid obtained was filtered off, dried, and recrystallized to afford compound **2*.***

Yield 87%; yellow crystal; mp 290 °C (MeOH); IR (cm^−1^) ν: 3318 (NH), 1688 (CO). ^1^H-NMR: 1.23–1.82(m, 10H, Cyclohexane), 4.72 (s, 1H. NH), 7.88 (s, 1H, CH-Pyrimidinone) 9.86 (s, 1H, NH), 11.76 (s, 1H, NH); ^13^C-NMR (100 MHz, DMSO-*d6*) δ (ppm): 20.52 (2), 24.46, 37.61 (2), 54.11, 110.90, 131.27, 161.23, 167.29, 174.74; Anal. Calcd for C_11_H_14_N_4_OS: C, 52.78; H, 5.64; N, 22.38; Found: C, 52.49; H, 5.33; N, 22.21.

#### 7'-methyl-2'-thioxo-2',3'-dihydro-1'H-spiro[cyclohexane-1,4'-pyrimido[4,5-d]pyrimidin]-5'(6'H)-one (3)

A mixture of compound **1** (2.22 gm; 0.01 mol) and 15 ml acetic acid was irradiated under microwave irradiation at 400W for 10 min. The solid obtained was filtered off, dried, and recrystallized to afford compound **3*.***

Yield 80%; orange crystal; mp 277 °C (MeOH); IR (cm^−1^) ν: 3327 (NH), 1685 (CO). ^1^H-NMR: 1.25–1.82(m, 10H, Cyclohexane), 2.61 (s, 3H. CH3), 4.75(s, 1H, NH), 9.89 (s, 1H, NH), 11.78 (s, 1H, NH); MS (*m*/*z*) (%): 264 (M + , 14), 113 (100); Anal. Calcd for C_12_H_16_N_4_OS: C, 54.52; H, 6.10; N 21.19; Found: C, 54.49; H, 5.85; N, 20.98.

#### 4-(1,3-dioxoisoindolin-2-yl)-2-thioxo-1,3-diazaspiro[5.5]undec-4-ene-5-carbonitrile (4)

A mixture of compound **1** (2.22 gm; 0.01 mol) and phthalic anhydride (1.48 gm; 0.01 mol) in ethylene glycol 10 ml and a catalytic amount of AcOH was reacted under microwave irradiation at 400W for 5 min. The reaction mixture was cooled and the solid obtained was filtered off, dried, and recrystallized to afford compound **4*.***

Yield 84%; white crystal; mp 232 °C (MeOH); IR (cm^−1^) ν: 3318 (NH), 2181 (CN), 1706 and 1793 (2CO). ^1^H-NMR: 1.23–1.82(m, 10H, Cyclohexane), 7.54–8.08 (m, 4H, Ar–H), 9.88 (s, 1H, NH), 11.77 (s, 1H, NH); ^13^C-NMR (100 MHz, DMSO-*d6*) δ (ppm): 20.06 (2), 24.77, 37.62 (2), 54.59, 59.43, 120.16, 124.28 (2), 131.95 (2), 133.85 (2), 150.20, 166.71 (2), 174.60; MS (*m*/*z*) (%): 252 (M + , 15), 58 (100); Anal. Calcd for C_18_H_16_N_4_O_2_S: C, 61.35; H, 4.58; N, 15.90; Found: C, 61.18; H, 4.70; N, 15.77.

#### ethyl (E)-N-(5-cyano-2-thioxo-1,3-diazaspiro[5.5]undec-4-en-4-yl)formimidate (5)

A mixture of compound **1** (2.22 gm; 0.01 mol) and 15 ml triethyl orthoformate was heated under reflux for 15 h. The excess of triethyl orthoformate was evaporated and the solid obtained was filtered off, dried, and recrystallized to afford compound **5*.***

Yield 77%; brown crystal; mp 186 °C (MeOH); IR (cm^−1^) ν: 3318 (NH), 2196 (CN). ^1^H-NMR: 1.08 (t, 3H, CH3), 1.27–1.71(m, 10H, Cyclohexane), 4.20 (q, 2H, CH2), 8.19 (s, 1H, CH-oliphinic), 9.05 (s, 1H, NH), 10.70 (s, 1H, NH); ^13^C-NMR (100 MHz, DMSO-*d6*) δ (ppm): 15.05, 20.06 (2), 24.77, 37.62 (2), 54.59, 62.82, 83.33, 117.27, 155.82, 164.52, 174.60; MS (*m*/*z*) (%): 278 (M +, 27), 77 (100); Anal. Calcd for C_13_H_18_N_4_OS: C, 56.09; H, 6.52; N, 20.13; Found: C, 55.89; H, 6.44; N, 19.89.

#### 2'-phenyl-7'H-spiro[cyclohexane-1,10'-pyrimido[5,4-e][1,2,4]triazolo[1,5-c]pyrimidine-8'(9'H)-thione (6)

A mixture of compound **5** (2.78 gm; 0.01 mol) and benzoyl hydrazide (1.36 gm; 0.01 mol) in dry dioxane 20 ml was irradiated under microwave irradiation at 400W for 5 min, and the solid obtained was filtered off, dried, and recrystallized to afford compound **6*.***

Yield 68%; yellow crystal; mp > 300 °C (DMF); IR (cm^−1^) ν: 3189 (NH), 1623 (C = N). ^1^H-NMR: 1.40–1.99 (m, 10H, Cyclohexane), 7.56–7.59 (m, 3H, Ar–H), 8.20 (d, 2H, Ar–H), 8.74 (s, 1H, CH-Pyrimidine) 9.56 (s, 1H, NH), 11.39 (s, 1H, NH); ^13^C-NMR (100 MHz, DMSO-*d6*) δ (ppm): 20.52 (2), 21.75, 34.43 (2), 61.92, 120.16, 127.15 (2), 130.22 (2), 133.05, 135.41, 140.06, 146.54, 160.04, 163.68, 180.64; MS (*m*/*z*) (%): 350 (M + , 45), 211 (100); Anal. Calcd for C_18_H_18_N_6_S: C, 61.69; H, 5.18; N, 23.98; Found: C, 61.39; H, 5.01; N, 23.76.

#### 2'-(2-hydroxyphenyl)-7'H-spiro[cyclohexane-1,10'-pyrimido[5,4-e][1,2,4]triazolo[1,5-c]pyrimidine]-8'(9'H)-thione (7)

A mixture of compound **5** (2.78 gm; 0.01 mol) and salicyloyl hydrazide (1.52 gm; 0.01 mol) in dry dioxane 20 ml was irradiated under microwave irradiation at 400W for 5 min, and the solid obtained was filtered off, dried, and recrystallized to afford compound **7*.***

Yield 72%; buff crystal; mp > 300 °C (DMF); IR (cm^−1^) ν: 3351 (OH), 3176 (NH), 1616 (C = N). ^1^H-NMR: 1.23–1.96(m, 10H, Cyclohexane), 7.01–7.05 (m, 2H, Ar–H), 7.44 (d, 1H, Ar–H), 8.10 (d, 1H, Ar–H), 8.87 (s, 1H, CH-Pyrimidine), 9.62 (s, 1H, NH), 11.09 (s, 1H, NH), 11.51 (s, 1H, OH); MS (*m*/*z*) (%): 366 (M + , 15), 60 (100); Anal. Calcd for C_18_H_18_N_6_OS: C, 59.00; H, 4.95; N, 22.93; Found: C, 58.76; H, 4.79; N, 22.69.

#### 6'-amino-5'-imino-5',6'-dihydro-1'H-spiro[cyclohexane-1,4'-pyrimido[4,5-d]pyrimidine]-2'(3'H)-thione (8)

A mixture of compound **5** (2.78 gm; 0.01 mol) and hydrazine hydrate (0.5 gm; 0.01 mol) in absolute ethanol 20 ml was sonicated at room temperature for 1 h, and the solid obtained was filtered off, dried, and recrystallized to afford compound **8.**

Yield 76%; yellow crystal; mp > 279 °C (EtOH); IR (cm^−1^) ν: 3384 (NH), 3285, 3264 (NH2), 1648 (C = N). ^1^H-NMR: 1.19–1.80 (m, 10H, Cyclohexane), 5.60 (s, 2H, NH2), 7.12 (s, 1H, NH), 8.04 (s, 1H, CH-Pyrimidine), 8.09 (s, 1H, NH), 10.31 (s, 1H, NH); ^13^C-NMR (100 MHz, DMSO-*d6*) δ (ppm): 22.62 (2), 27.95, 37.77 (2), 50.72, 100.98, 142.82, 158.32, 161.98, 174.37; MS (*m*/*z*) (%): 264 (M + , 29), 69 (100); Anal. Calcd for C_11_H_16_N_6_S: C, 49.98; H, 6.10; N, 31.79; Found: C, 49.85; H, 5.98; N, 31.67.

#### 5'-imino-6'-(phenylamino)-5',6'-dihydro-1'H-spiro[cyclohexane-1,4'-pyrimido[4,5-d]pyrimidine]-2'(3'H)-thione (9)

A mixture of compound **5** (2.78 gm; 0.01 mol) and phenyl hydrazine (1.08 gm; 0.01 mol) in absolute ethanol 20 ml was sonicated at room temperature for 1 h, and the solid obtained was filtered off, dried, and recrystallized to afford compound **9.**

Yield 70%; orange crystal; mp > 264 °C (EtOH); IR (cm^−1^) ν: 3274 (NH), 1651 (C = N). ^1^H-NMR: 1.11–1.81 (m, 10H, Cyclohexane), 6.64 (d, 2H, Ar–H), 6.73 (s, 1H, NH), 6.90 (d,d, 1H, Ar–H), 7.26 (d,d, 2H, Ar–H), 8.05 (s, 1H, CH-Pyrimidine), 8.18 (s, 1H, NH), 9.10 (s, 1H, NH), 10.51 (s, 1H, NH); MS (*m*/*z*) (%): 340 (M + , 30), 100 (100); Anal. Calcd for C_17_H_20_N_6_S: C, 59.98; H, 5.92; N, 24.69; Found: C, 59.91; H, 5.88; N, 24.60.

### Biological evaluation

#### Mosquito rearing

The colony of *Culex pipiens* was obtained from the Research Institute for Medical Entomology, Dokki, Giza, Egypt. Then reared in the insectary of Entomology Department, Faculty of Science, Ain Shams University according to^48^ with slight modifications, under controlled rearing conditions of light and dark cycle (14 h: 10 h), 27 ± 2 °C and relative humidity 75 ± 5% in wooden cage (50 cm × 50 cm × 50 cm) equipped with a distillate water-filled jars for adult emergence and egg laying. The 1st larval instars were fed on the larval diet (a mixture of dried yeasts and biscuits in ratios (1: 3))^49^, and the distilled water in the rearing jars was replaced every four days. Adults were provided with 10% glucose solution and only females were allowed to feed on alive pigeon for blood meal and egg production for stock-rearing.

#### Larvicidal activity

The toxicity of nine synthesized compounds and reference insecticide was evaluated against the third instar larvae of *C. pipiens* according to^50^ with slight modifications. The synthesized spiro-pyrimidine compounds were dissolved in DMSO to prepare the stock solution, while the commercial formulation of IGRs: pyriproxyfen (Sumilarv® 0.5G, Sumitomo Chemical Co., Chuo-ku, Osaka, Japan) was dissolved in distilled water. Then seven dilutions of synthesized compounds were prepared as follows (1, 5, 10, 25, 50, 75 and 100 µg/mL) for 2, 3 and 4 and (10, 25, 50, 75 100, 150 and 200 µg/mL) for 1, 5, 6, 7, 8 and 9 in DMSO and Triton X-100, while the pyriproxyfen (0.05, 0.1, 0.25, 0.5 and 1 µg/mL) in distilled water. Only solvents without the tested compounds were tested as a control. Three replicates for each concentration of all tested compounds and control were performed. For 80 mL of each concentration, a group of 20 experimental larvae was placed in 100 mL cups^39^. Tested larvae were considered dead when they were unable to swim or reach the water's surface. The cups were examined after 24 h and the mortality percentages were estimated.

#### Biological effects

The early third larval instar of *C. pipiens* was treated with seven previously mentioned concentrations of the most potent compounds **2, 3, 4** and five concentrations for pyriproxyfen and given a larval diet during the experimental time. Each concentration of tested compounds was trireplicated to study the biological effects of each replicate. The cumulative mortalities of mosquito larvae and pupae were reported daily. Then, the survived pupae were transferred to fresh distilled water for further observations. Both incompletely emerged and completely emerged adults but unable to fly away from the water surface were recorded as dead. The biological alterations caused by the tested compounds were expressed by the larval and pupal mortalities, which didn’t successfully develop into normal emerged adults. Also, the larval and pupal durations, pupation rates, adult emergence and longevity, and growth index were estimated^51^. The Growth index was estimated according to the equation of El-Sheikh^52^.

$${\text{Growth}}\;{\text{index}} = {\text{a}}/{\text{b}}$$

where: a: % of adult emergence. b: Adult longevity(days) (days).

In addition, the morphological abnormalities were demonstrated by stereomicroscope and binocular (BEL®) photonic microscope and photographed by the digital camera (Sony Dec-W610).

#### Cytotoxicity assay

The human lung fibroblast cell line (WI-38) was obtained from ATCC via biological products and vaccines (VACSERA) company, Cairo, Egypt. Using the MTT assay, the normal cell line was employed to investigate the synthesized spiropyrimidin's inhibitory effects on cell growth. In this experiment, mitochondrial succinate dehydrogenase in the living cells causes the yellow tetrazolium bromide (MTT) to change into a purple formazan derivative. These cells were incubated in RPMI-1640 medium with 10% of fetal bovine serum^53^**.** After incubation, the normal cells were treated with (100, 50, 25, 12.5, 6.25, 3.12 and 1.56 µM) of the most effective compounds 2, 3 and 4 and incubated for 24 h, then 20 µL of MTT solution at (5 mg/mL) was added and incubated again for 4 h. Dimethyl sulfoxide (DMSO) (in the volume of 100 µL) is mixed with each well to dissolve the formed purple formazan. The colorimetric assay is measured at an absorbance of (570 nm) using a plate reader (EXL 800, USA). The relative cell viability was calculated as ((A570) of treated samples/(A570) of untreated sample) X 100^54^.

### Statistical analysis

The mortality rates were subjected to the LdPLine© package software (Ehabsoft, Egypt) for log-probit analysis following^55^. The lethal concentrations with their 95% fiducially limits (F.l. at 95%), slope with standard errors (SE) and Chi-square (*x*^*2*^) were estimated. The means of the biological data were compared according to the One-way analysis of variance (ANOVA) by using IBM SPSS statistics (Version 26 for Windows). The data was significantly different at (P < 0.05).

### In silico study

#### Molecular docking

Through the ligand-receptor interactions that aid in the prediction of their potency, molecular docking studies have been started to explain the mode of action of the tested drugs. For drawing ligands and tested compounds, Chemdraw 20.0 (CambridgeSoft) (Perkin Inc.) was employed. For improved fitting and docking outcomes, the Wave Function Spartan v 14.0 program was utilized to optimize the shape and globally minimize the energies of the ligand and tested compounds^56^. The examined compounds underwent 3D protonation and the addition of partial charges. In the docking study, the crystal structure of the binding protein for chitinase (*Ostrinia furnacalis* chitinase-h) was employed. The Protein Data Bank (PDB ID: 6 JMN)^35^ was used to download the X-ray crystal structure of (*Of* Chi-h). Using AutoDock Vina, a molecular docking study was conducted for the pyriproxyfen, co-crystallized ligand (2–8-S2), and tested compounds into (*Of* Chi-h)^57^. Correction, 3D hydrogenation, in addition to energy minimization, was used to create the protein receptor^58,59^. The PyMOL program was utilized for the visualization procedure^60^.

#### DFT calculations

The Gaussian 09W programme^61^ was used to do calculations based on the density functional theory (DFT). DFT computations were performed at the B3LYP level, which combines the Lee–Yang–Parr correlation functional with Becke's three-parameter hybrid exchange functional (local, non-local, and Hartree–Fock).

Using 6–31 + G(d,p) as a basis set, full geometry optimization was carried out to produce the optimized ground state characteristics and structures of the investigated compounds. 'd' polarisation functions on heavy atoms and 'p' polarisation functions on hydrogen atoms, along with diffuse functions for both hydrogen and heavy atoms, were added to the basis set 6–31 + G(d, p).

Some conceptual DFT-related theoretical descriptors for the most potent compounds and reference insecticides have been established to anticipate the chemical reactivity. The electronegativity (E), the highest occupied molecular orbital (E_HOMO_), the lowest unoccupied (empty) molecular orbital (E_LUMO_), the global softness (S), and the hardness (H) are all included. The optimized molecules serve as the basis for all of these descriptions. It should be highlighted that the Koopmans approximation was used to determine the descriptors for frontier molecular orbitals (FMO) in a relatively simple approach^62^.

### Ethical approval

All experiments were ethically approved by the Institutional Ethical Committee of the Faculty of Science, Ain Shams University CODE: (ASU-SCI/CHEM/2023/5/8).

## Conclusion

A new series of nine spiro pyrimidine derivatives were synthesized in good yield and short time using different synthetic methodologies like microwave irradiation and sonication. The activity of synthesized compounds was evaluated against 3rd larval instar of *C. pipiens.* Where, **3**, **4** and **2** showed significant toxicity with high biological impacts against *C. pipiens* larvae and various morphological malformations in all developmental stages were observed. On the other hand, compound **1** exhibited low potency. While, **5**, **7**, **6**, **9** and **8** show moderate activity. In addition, **3** and **4** exhibited significant prolongation of the developmental duration and greatly inhibited adult emergence. Furthermore, compounds **2** and **4** showed weak cytotoxicity in normal human cells (WI-38) as non-target organisms, while compound **3** was non-toxic. The molecular docking study interpreted the mode of action of tested compounds relative to pyriproxyfen against *Ostrinia furnacalis* chitinase h (Of Chi-h). The cocrystalized ligand and new candidates showed similar binding interactions with *Of* Chi- h binding site. Finally, DFT calculations were carried out to correlate the relation between reactivity and chemical structure based on global descriptors.

### Supplementary Information

Supplementary Information.

## Data Availability

All data generated or analyzed during this study are available and included in supporting information file as supplementary data.
