# Supplementary material for: Synthesis, toxicological and in silico evaluation of novel spiro pyrimidines against Culex pipiens L. referring to chitinase enzyme
Source: Sci Rep. 2024 Jan 17;14:1516. doi: 10.1038/s41598-024-51771-8 (PMC10794250; doi:10.1038/s41598-024-51771-8)
Supplement: Supplementary file 1 — Supplementary Information. [file 41598_2024_51771_MOESM1_ESM.docx]

Supporting Information

**Synthesis, Toxicological and *in Silico* Evaluation of Novel Spiro Pyrimidines against *Culex pipiens* L. referring to Chitinase Enzyme**

Eslam M. Abbass^a^*, Ali Khalil Ali^a^, Ahmed F. El-Farargy^b^, Doaa R. Abdel-Haleem^c^, Safaa S. Shaban^a^

a Chemistry Department, Faculty of Science, Ain shams University, Abbassia, 11566 Cairo, Egypt.

b Chemistry Department, Faculty of Science, Zagazig University, Zagazig, 44519, Egypt.

c Entomology Department, Faculty of Science, Ain shams University, Abbassia, 11566 Cairo, Egypt.

* Corresponding authors:

**Eslam M. Abbass: Email:** eslammorad@sci.asu.edu.eg

**ORCID:** 0000-0002-3245-1653

**Table of Contents**

| **Title** | **Page** |
| --- | --- |
| **IR spectral data of the target compounds (1-9)** | **S3** |
| **Figure S1.** **IR** spectrum of compound **1** | **S3** |
| **Figure S2.** **IR** spectrum of compound **2** | **S4** |
| **Figure S3.** **IR** spectrum of compound **3** | **S4** |
| **Figure S4.** **IR** spectrum of compound **4** | **S5** |
| **Figure S5.** **IR** spectrum of compound **5** | **S5** |
| **Figure S6.** **IR** spectrum of compound **6** | **S6** |
| **Figure S7.** **IR** spectrum of compound **7** | **S6** |
| **Figure S8.** **IR** spectrum of compound **8** | **S7** |
| **Figure S9.** **IR** spectrum of compound **9** | **S7** |
| **^1^H NMR spectral data of the target compounds (1-9)** | **S8** |
| **Figure S10.** ^1^H NMR (300 MHz, DMSO-*d_6_*) spectrum of compound **1** | **S8** |
| **Figure S11.** ^1^H NMR (400 MHz, DMSO-*d_6_*) spectrum of compound **2** | **S8** |
| **Figure S12.** ^1^H NMR (400 MHz, DMSO-*d_6_*) spectrum of compound **3** | **S9** |
| **Figure S13.** ^1^H NMR (400 MHz, DMSO-*d_6_*) spectrum of compound **4** | **S9** |
| **Figure S14.** ^1^H NMR (400 MHz, DMSO-*d_6_*) spectrum of compound **5** | **S10** |
| **Figure S15.** ^1^H NMR (400 MHz, DMSO-*d_6_*) spectrum of compound **6** | **S10** |
| **Figure S16.** ^1^H NMR (400 MHz, DMSO-*d_6_*) spectrum of compound **7** | **S11** |
| **Figure S17.** ^1^H NMR (400 MHz, DMSO-*d_6_*) spectrum of compound **8** | **S11** |
| **Figure S18.** ^1^H NMR (400 MHz, DMSO-*d_6_*) spectrum of compound **9** | **S12** |
| **^13^C NMR spectral data of the target compounds** | **S12** |
| **Figure S19.** ^13^C NMR (100 MHz, DMSO-*d_6_*) spectrum of compound **1** | **S12** |
| **Figure S20.** ^13^C NMR (100 MHz, DMSO-*d_6_*) spectrum of compound **2** | **S13** |
| **Figure S21.** ^13^C NMR (100 MHz, DMSO-*d_6_*) spectrum of compound **4** | **S13** |
| **Figure S22.** ^13^C NMR (100 MHz, DMSO-*d_6_*) spectrum of compound **5** | **S14** |
| **Figure S23.** ^13^C NMR (100 MHz, DMSO-*d_6_*) spectrum of compound **6** | **S14** |
| **Figure S24.** ^13^C NMR (100 MHz, DMSO-*d_6_*) spectrum of compound **8** | **S15** |
| **Mass fragmentation of the target compounds** | **S15** |
| **Figure S25.** Mass fragmentation of compound **3** | **S15** |
| **Figure S26.** Mass fragmentation of compound **4** | **S16** |
| **Figure S27.** Mass fragmentation of compound **5** | **S16** |
| **Figure S28.** Mass fragmentation of compound **6** | **S17** |
| **Figure S29.** Mass fragmentation of compound **7** | **S17** |
| **Figure S30.** Mass fragmentation of compound **8** | **S18** |
| **Figure S31.** Mass fragmentation of compound **9** | **S18** |
| **2D and 3D interactions with target enzyme (*Ostrinia furnacalis* chitinase h)** | **S19** |

**IR spectral data of the target compounds (1-9)**


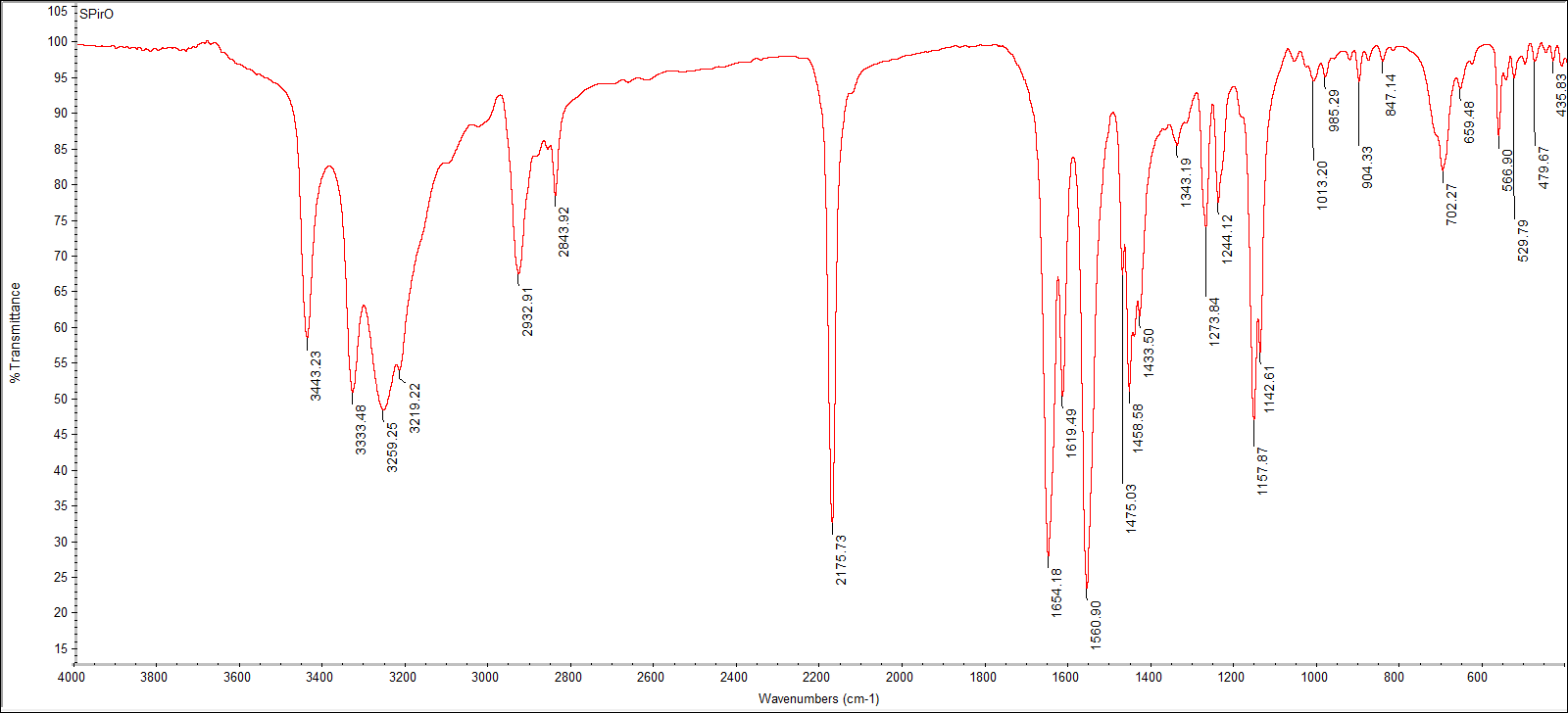


**Figure S1.** IR spectrum of compound **1**


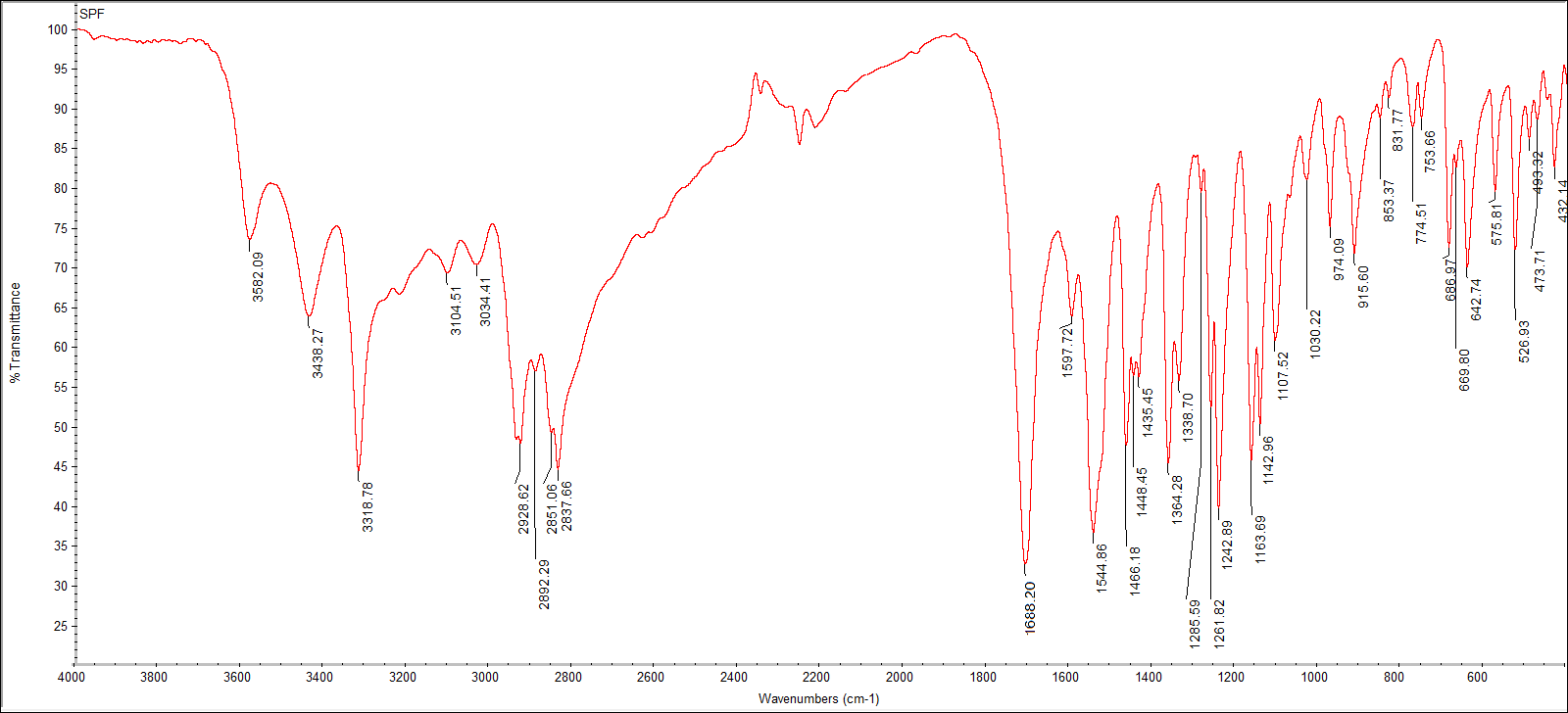


**Figure S2.** IR spectrum of compound **2**


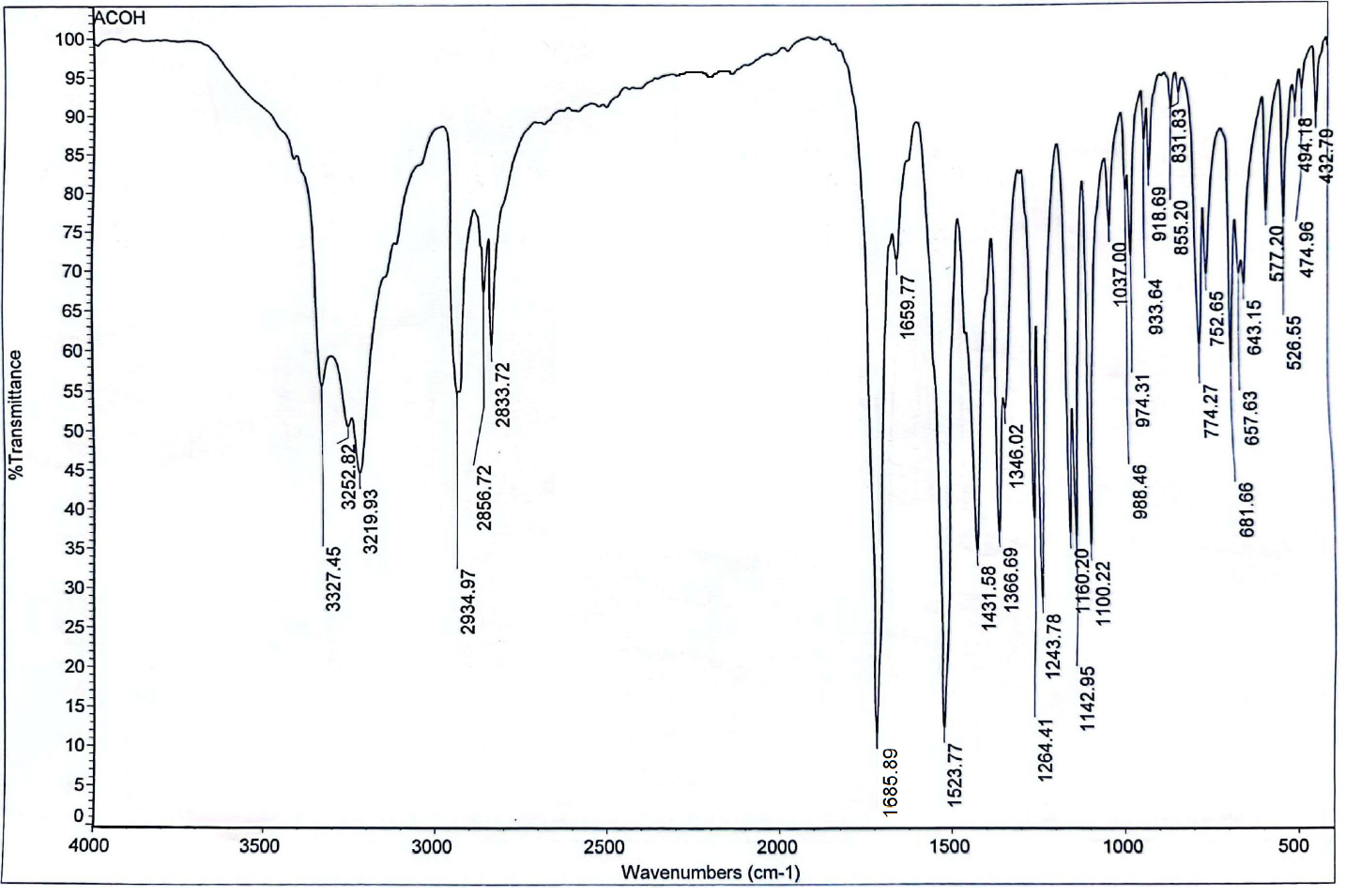


**Figure S3.** IR spectrum of compound **3**


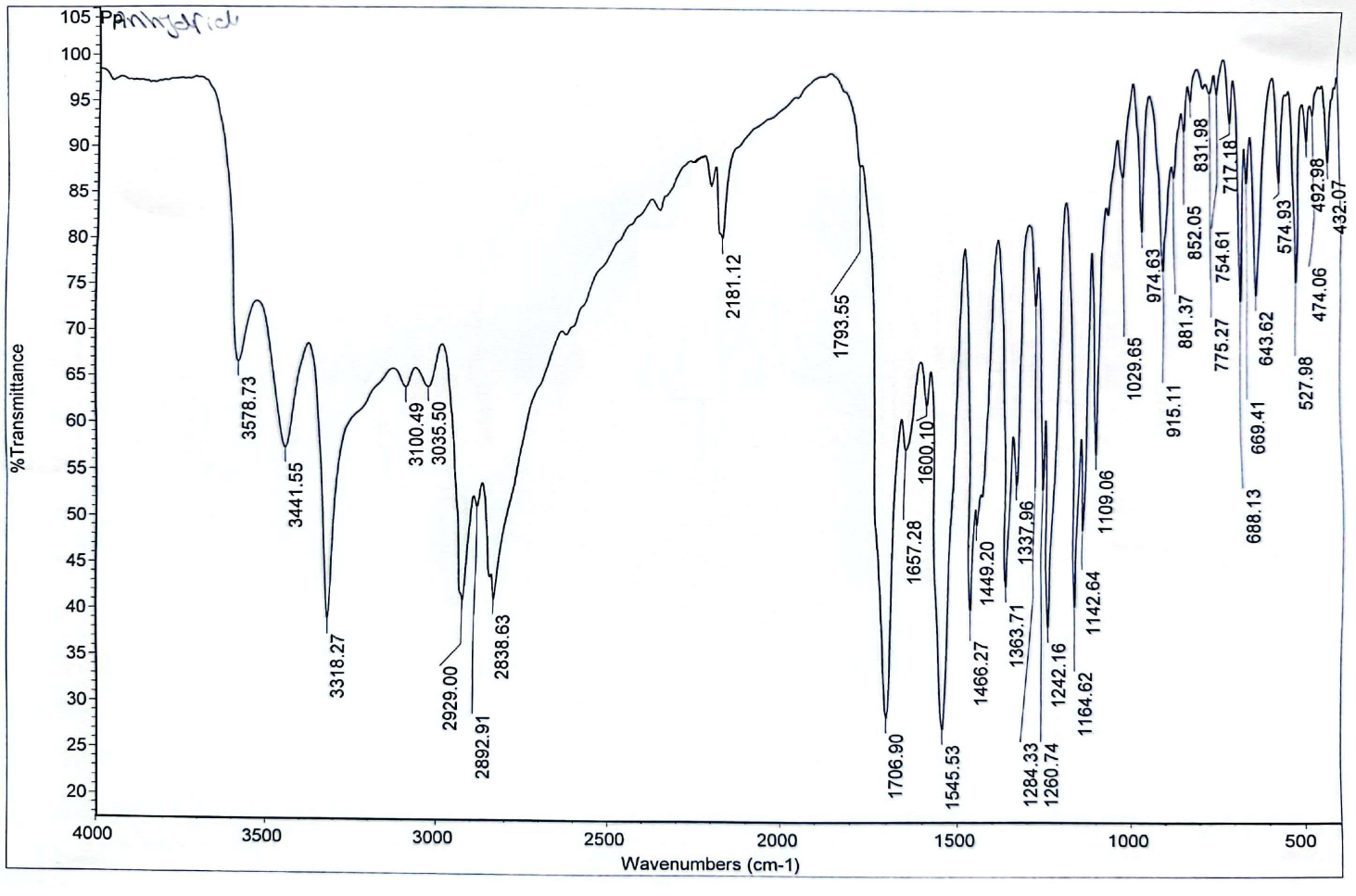


**Figure S4.** IR spectrum of compound **4**


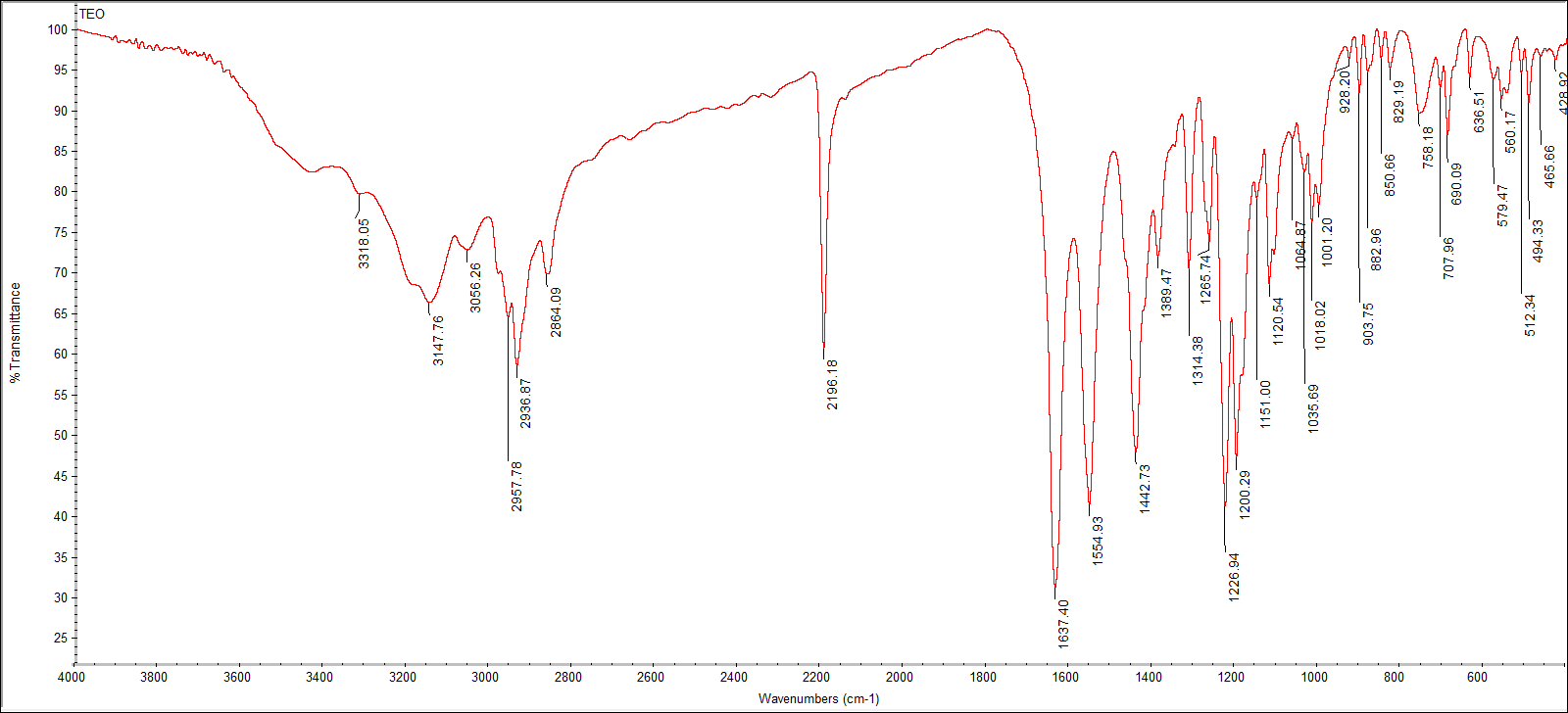


**Figure S5.** IR spectrum of compound **5**

**
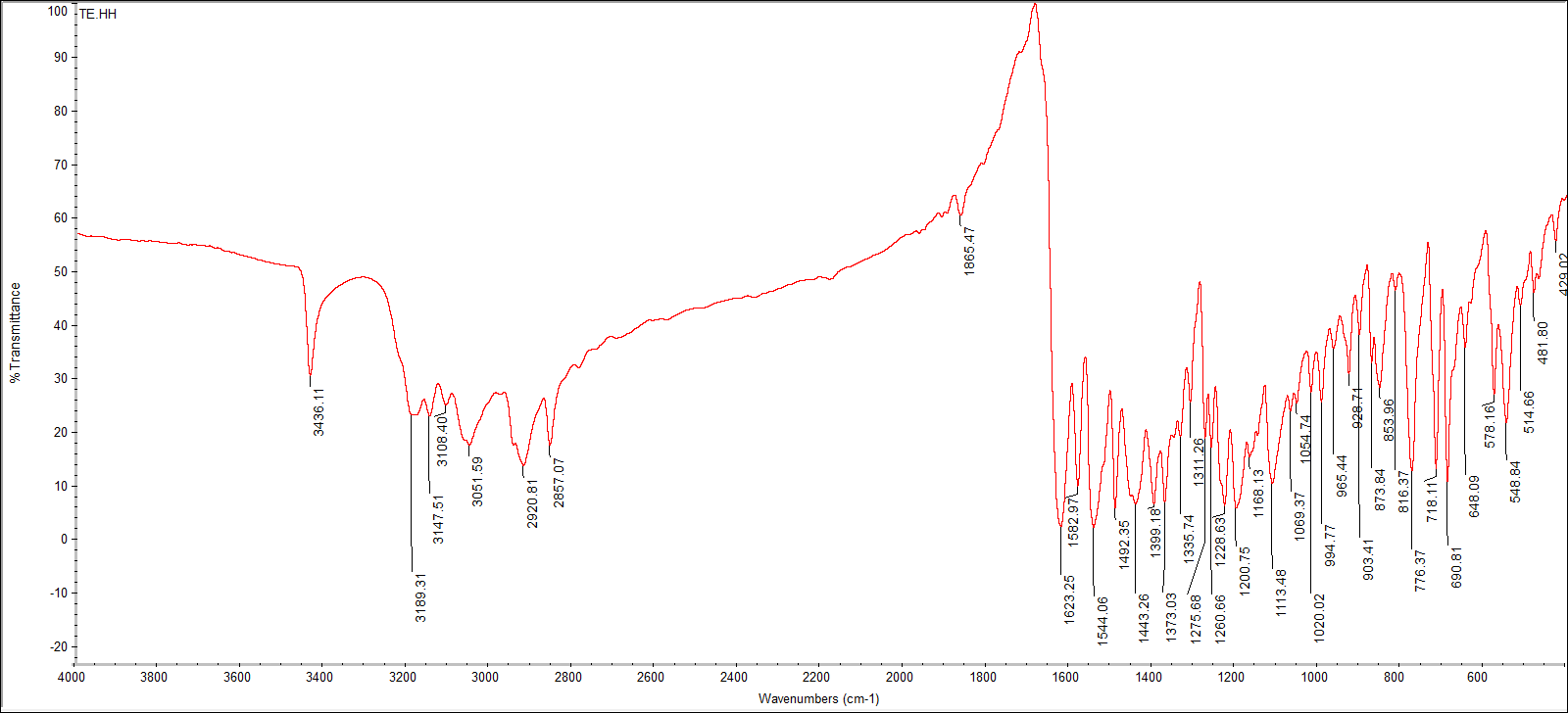
**

**Figure S6.** IR spectrum of compound **6**

**
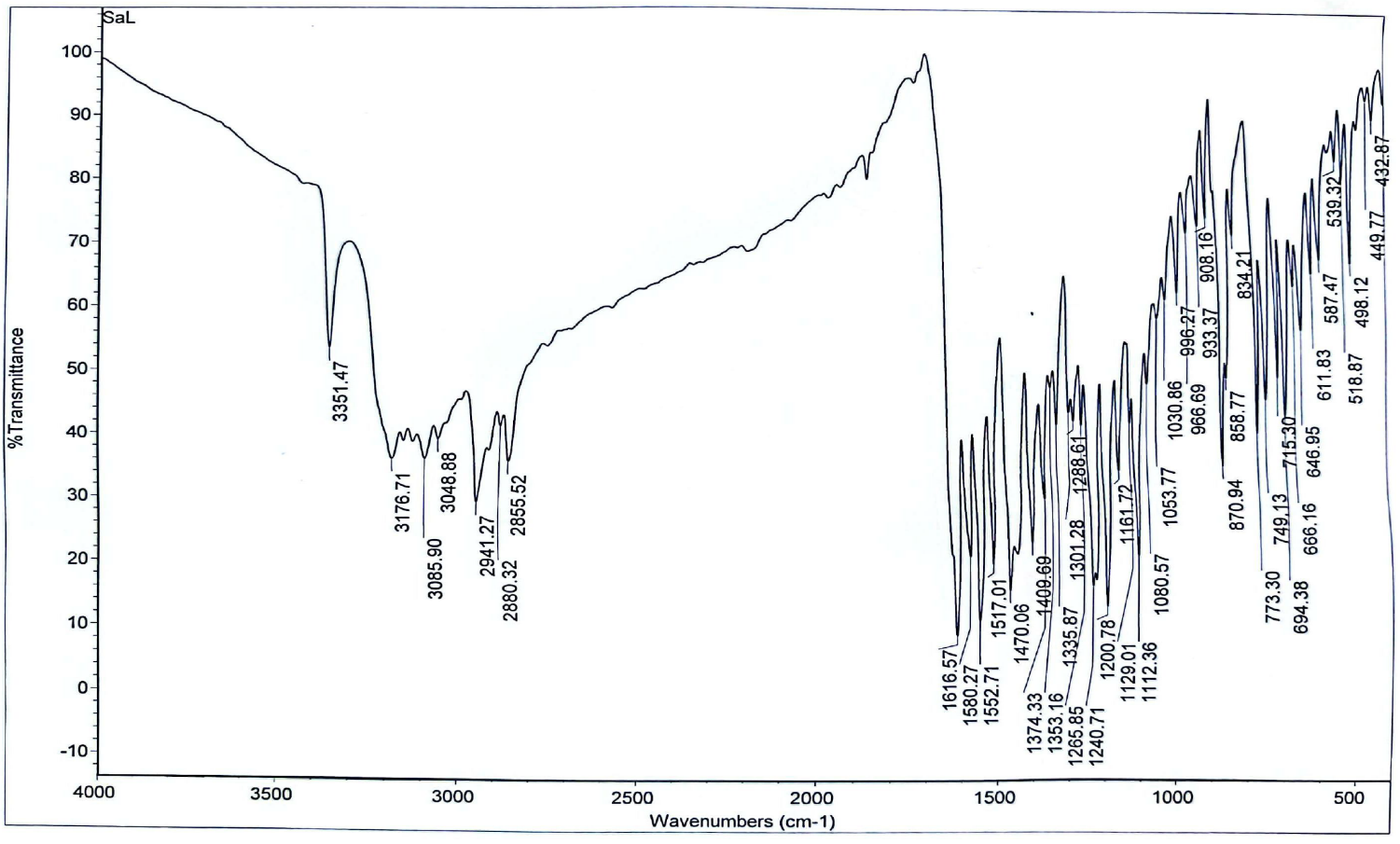
Figure S7.** IR spectrum of compound **7**

**
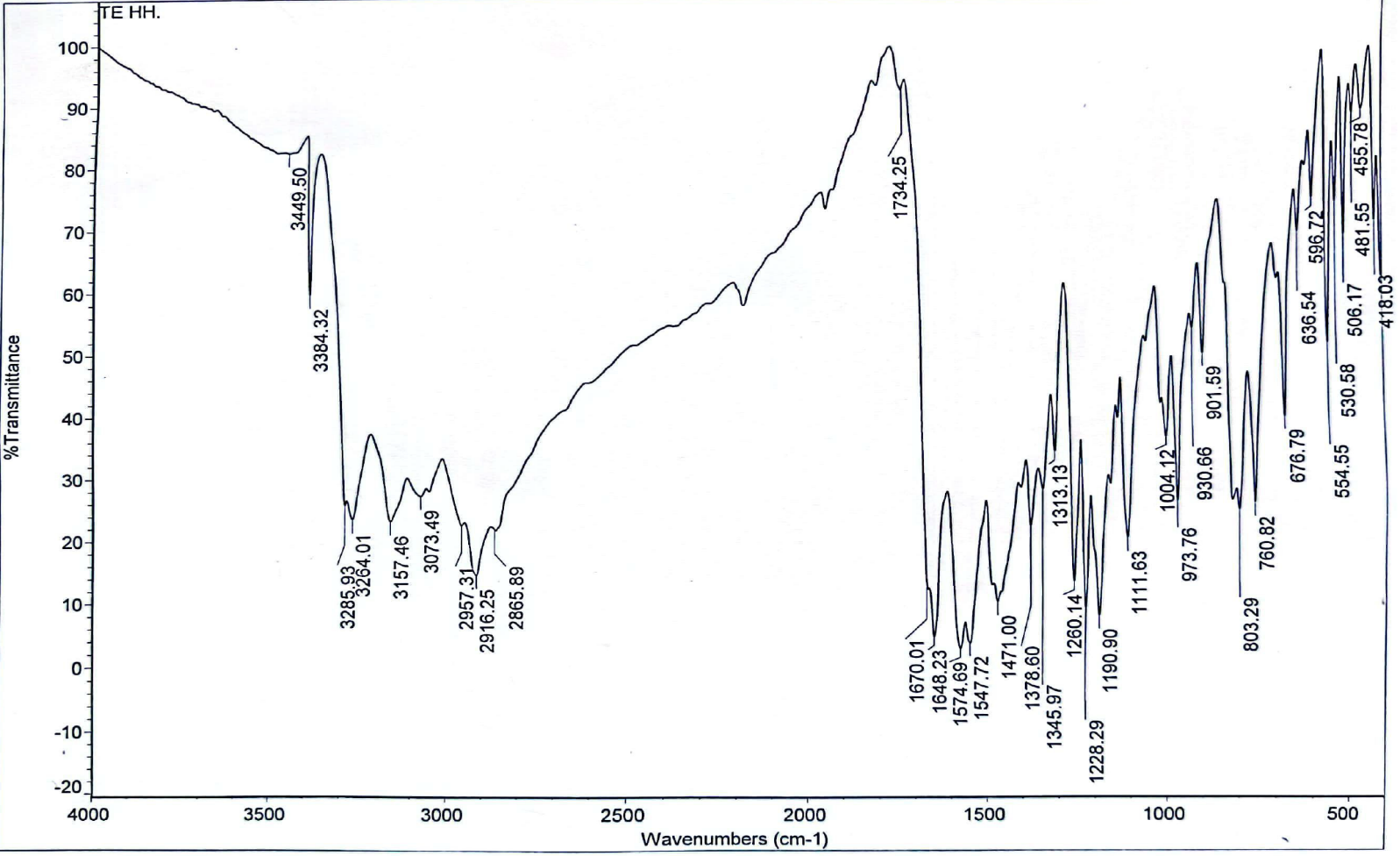
**

**Figure S8.** IR spectrum of compound **8**

**
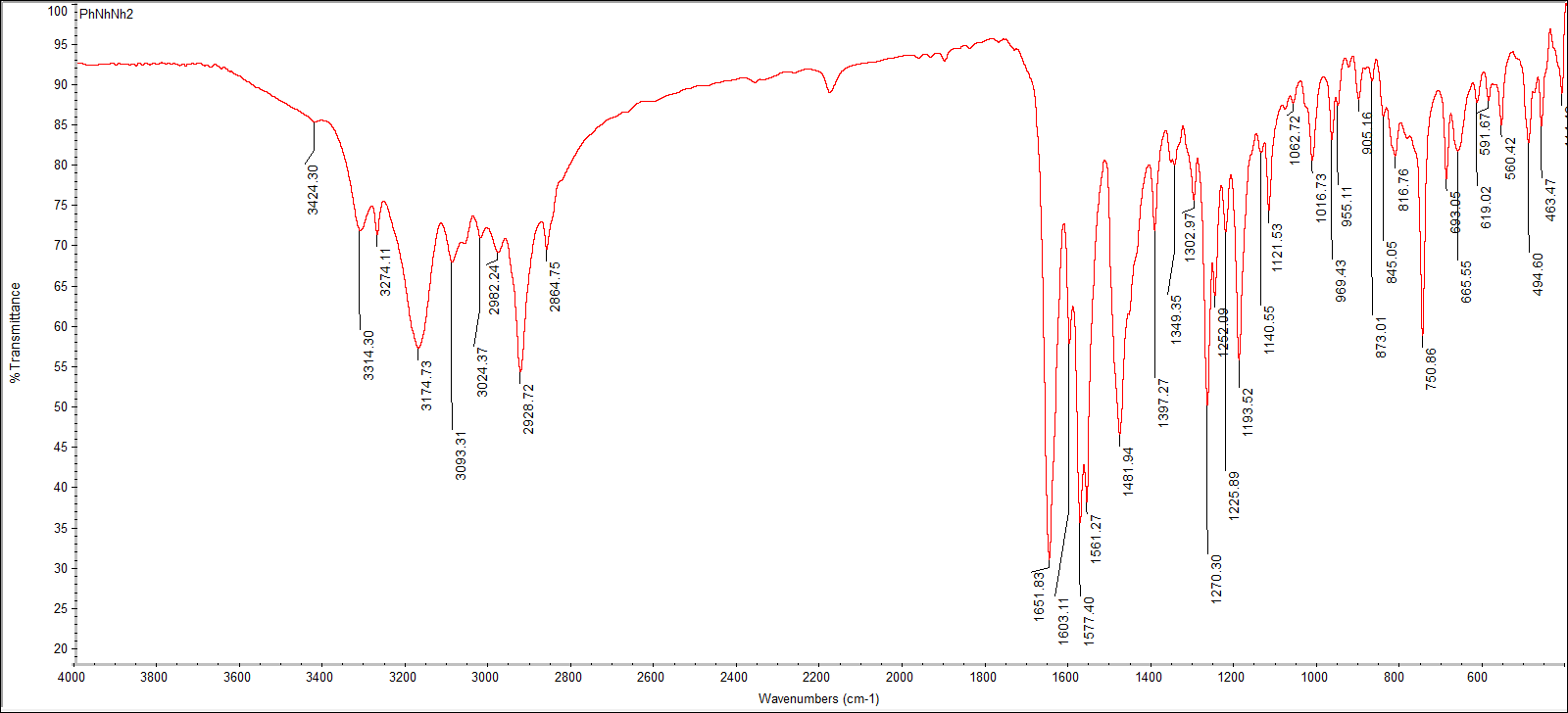
**

**Figure S9.** IR spectrum of compound **9**

**^1^H NMR spectral data of the target compounds (1-9)**


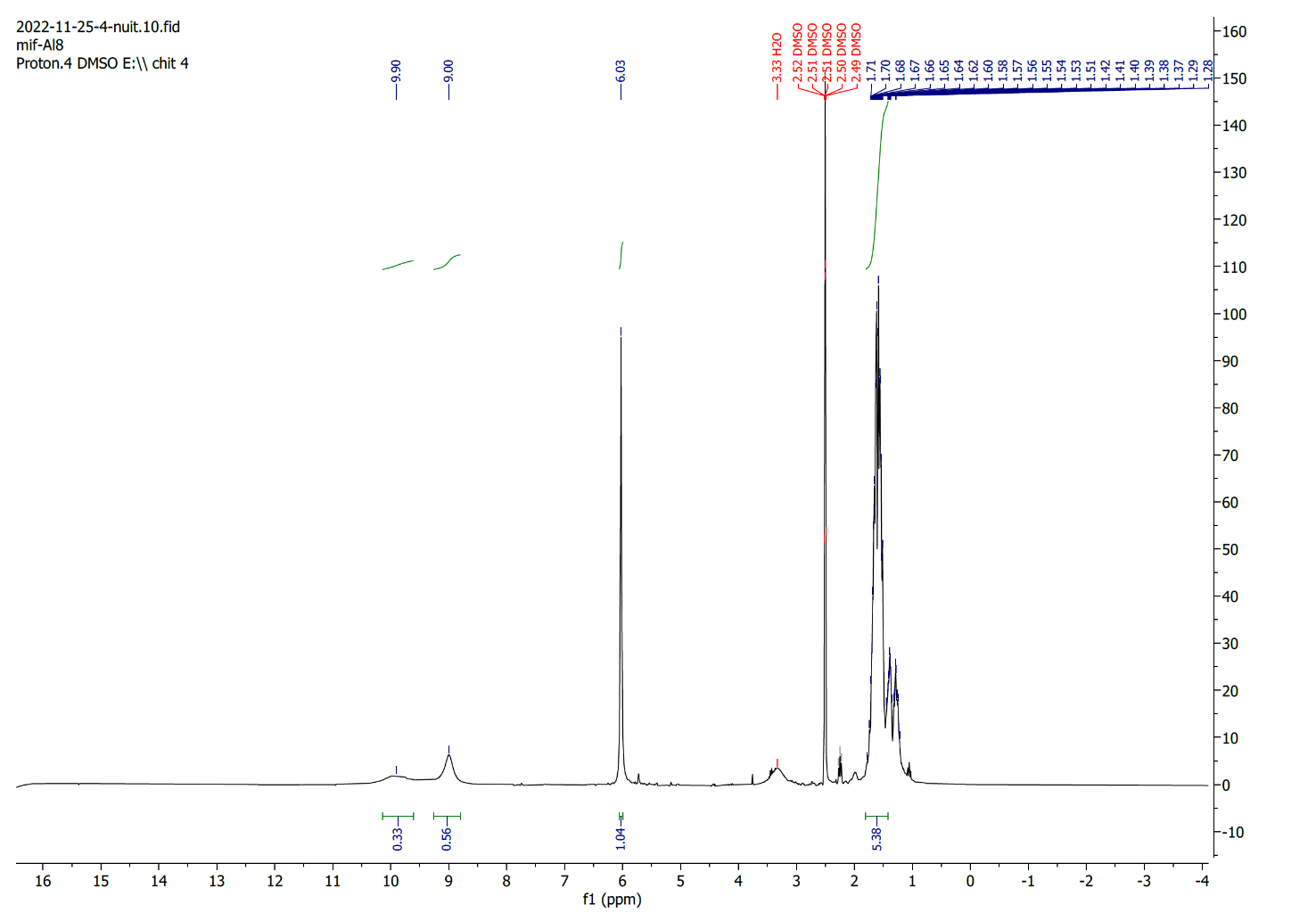


**Figure S10.** ^1^H NMR (300 MHz, DMSO-*d_6_*) spectrum of compound **1**


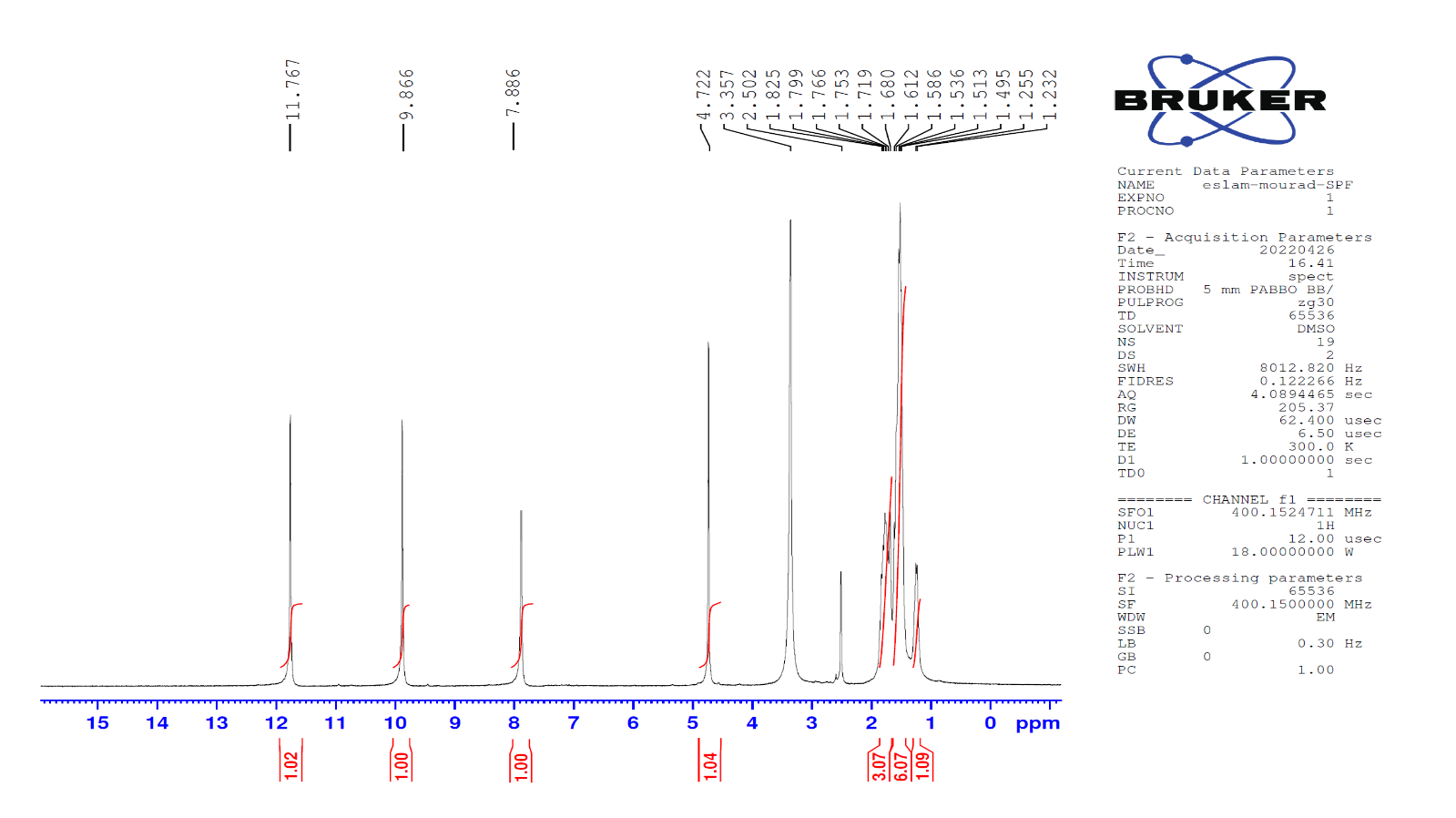


**Figure S11.** ^1^H NMR (400 MHz, DMSO-*d_6_*) spectrum of compound **2**


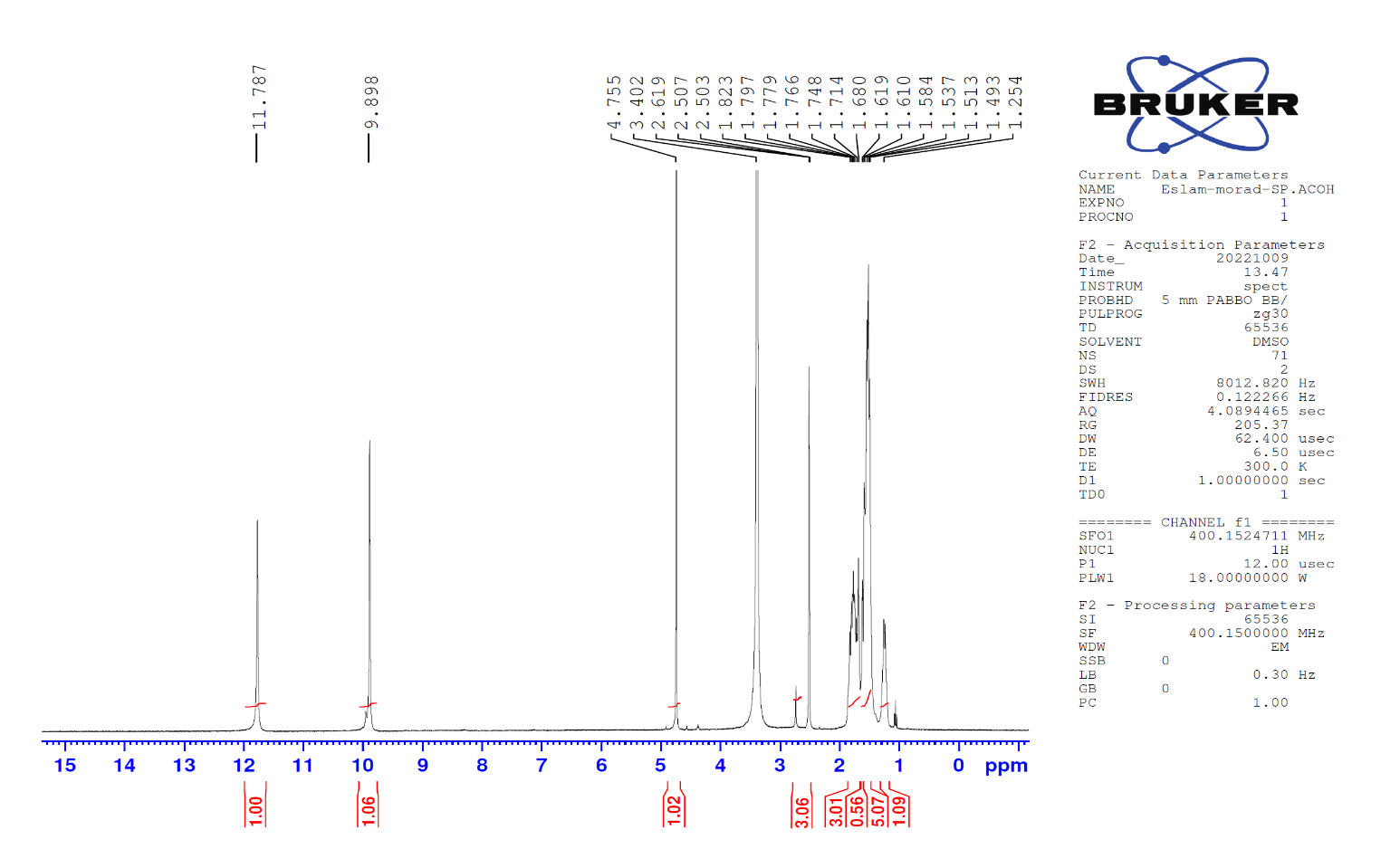


**Figure S12.** ^1^H NMR (400 MHz, DMSO-*d_6_*) spectrum of compound **3**


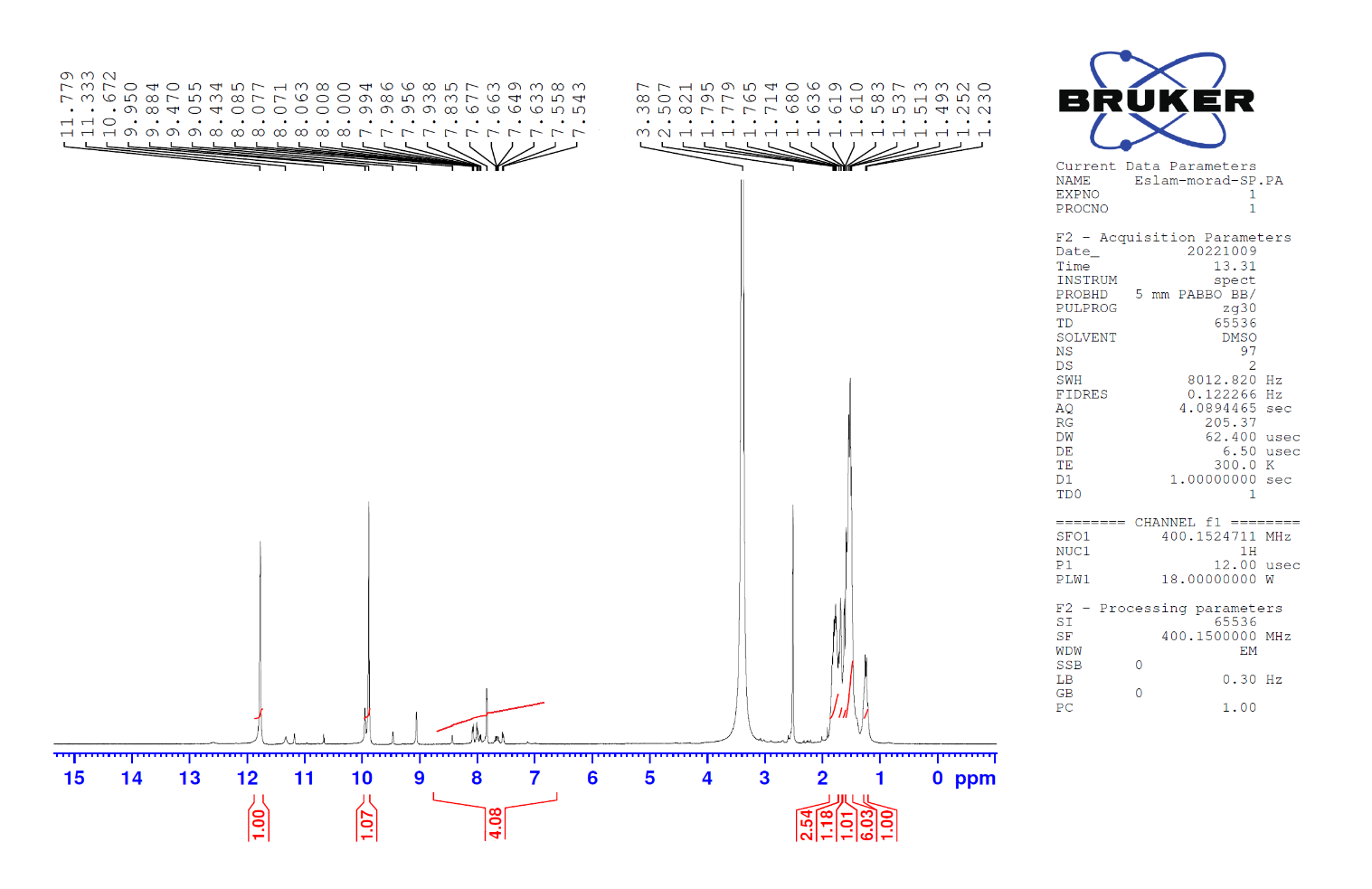


**Figure S13.** ^1^H NMR (400 MHz, DMSO-*d_6_*) spectrum of compound **4**


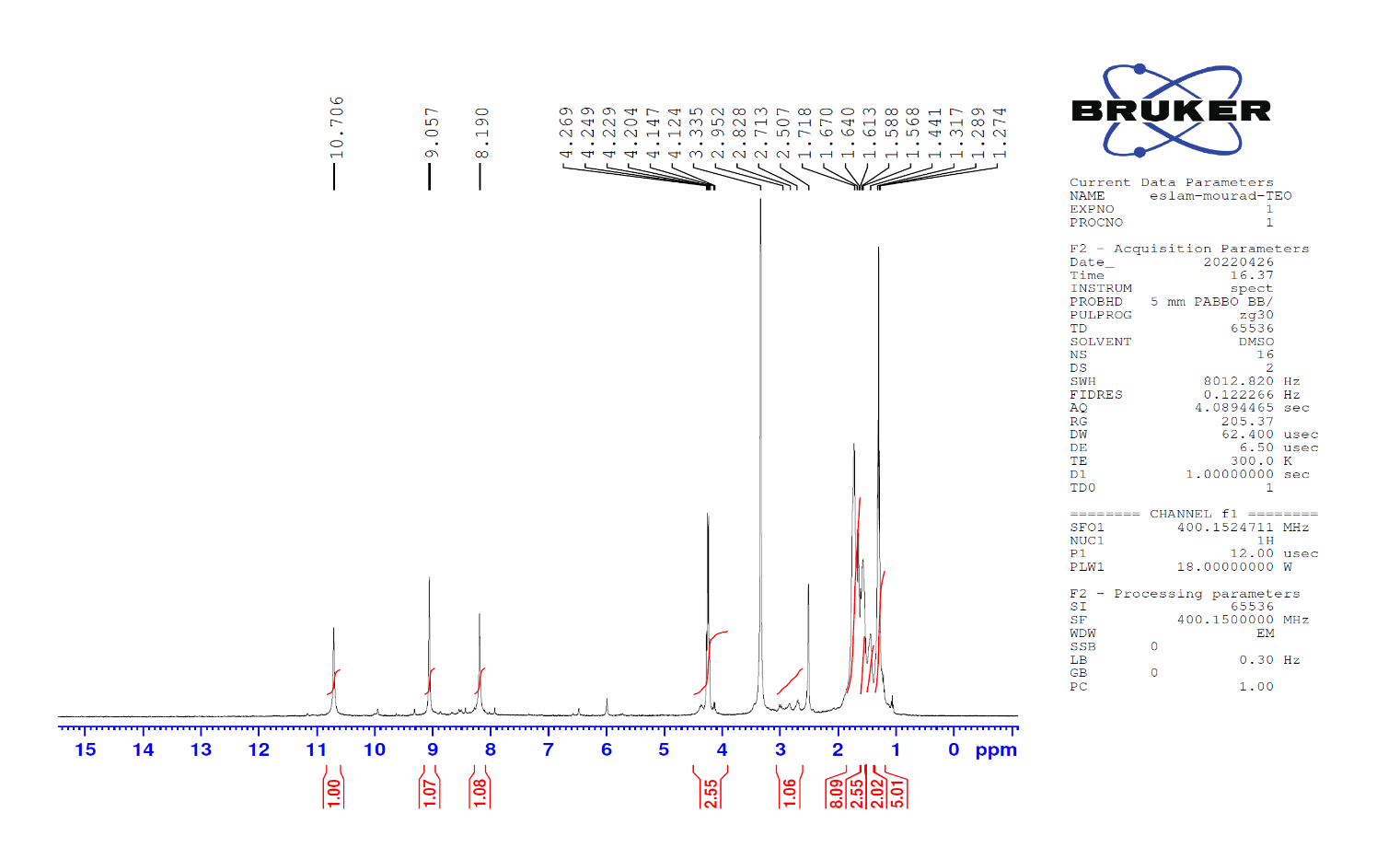


**Figure S14.** ^1^H NMR (400 MHz, DMSO-*d_6_*) spectrum of compound **5**


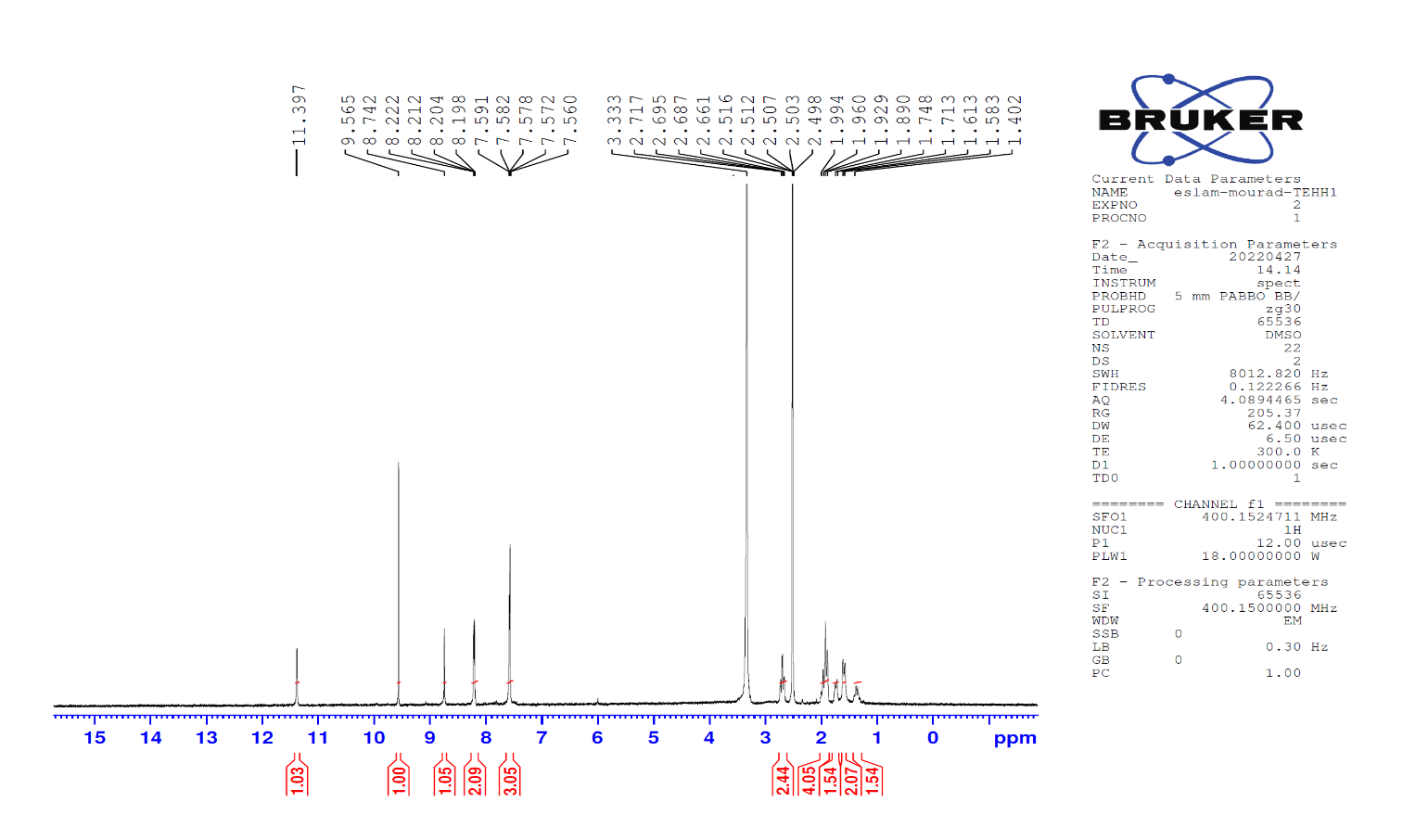


**Figure S15.** ^1^H NMR (400 MHz, DMSO-*d_6_*) spectrum of compound **6**


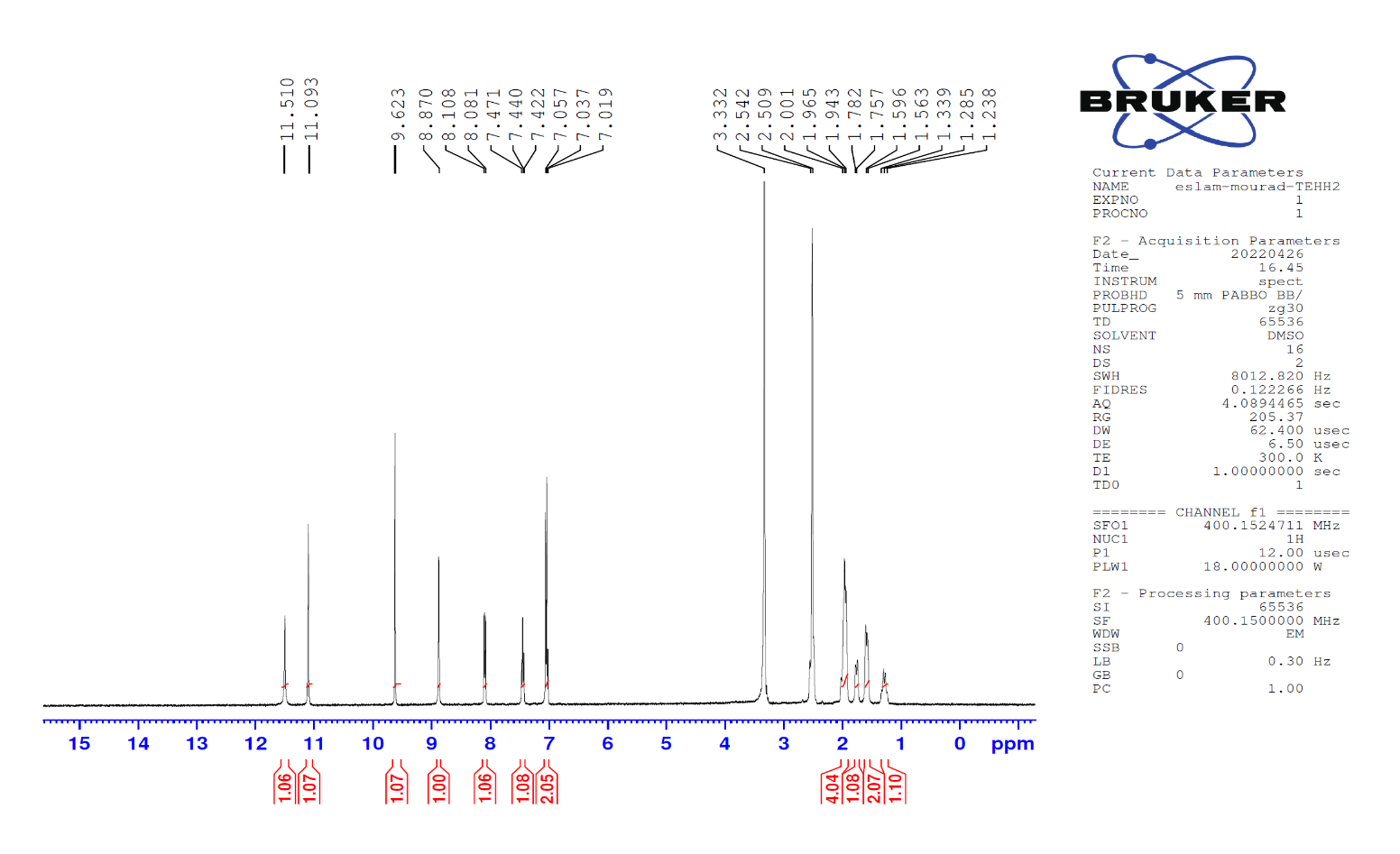


**Figure S16.** ^1^H NMR (400 MHz, DMSO-*d_6_*) spectrum of compound **7**


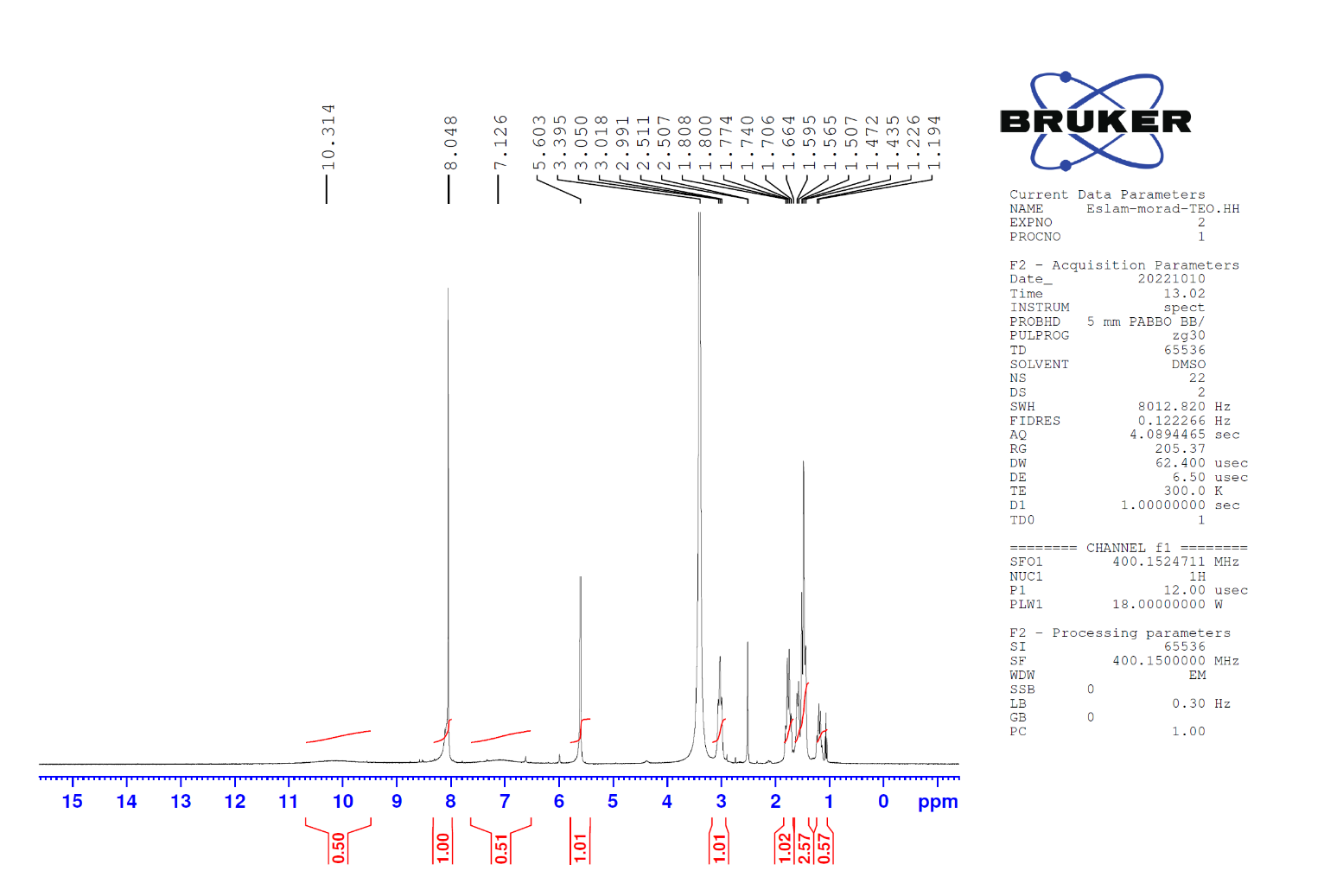


**Figure S17.** ^1^H NMR (400 MHz, DMSO-*d_6_*) spectrum of compound **8**

***
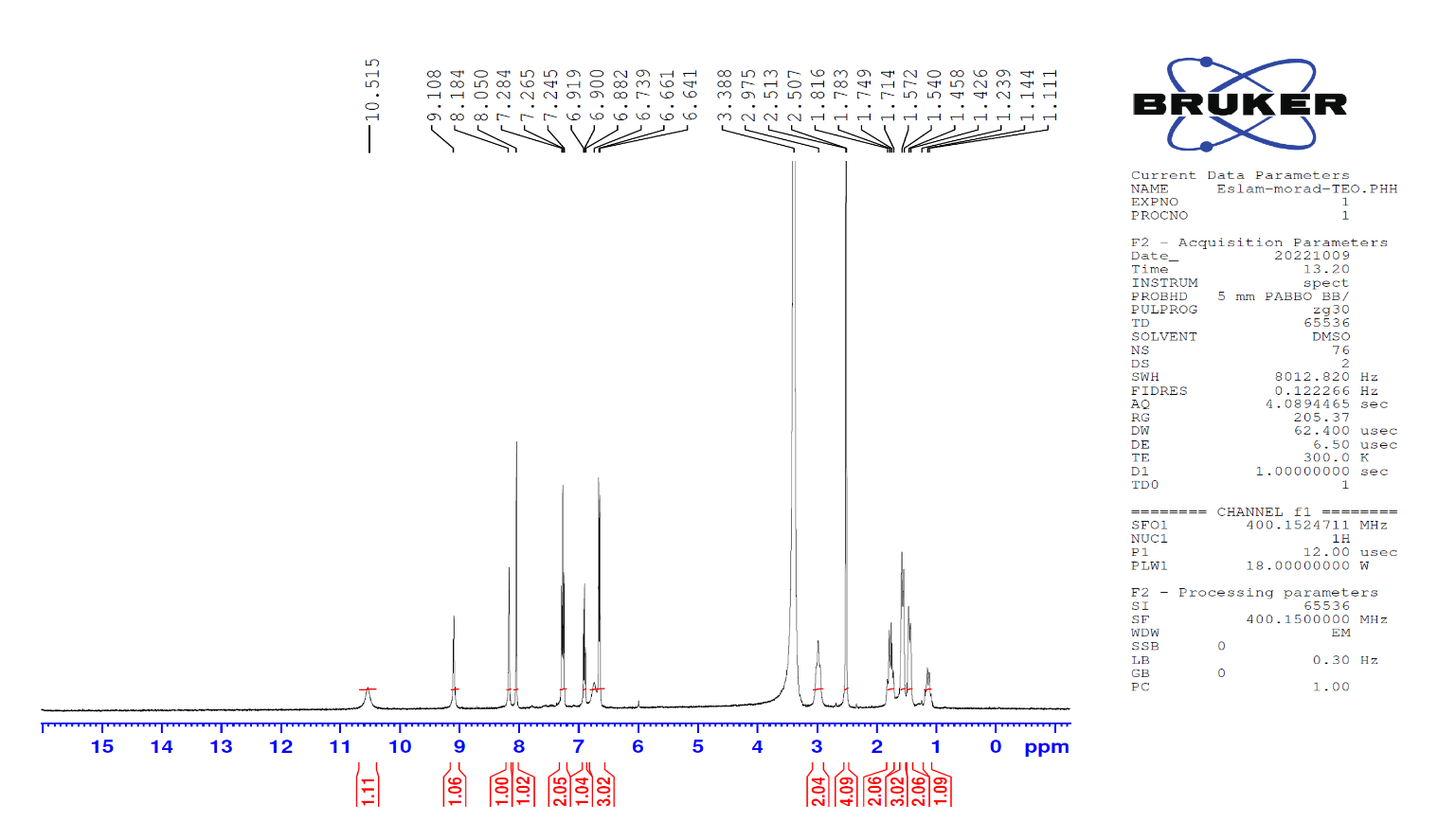
***

**Figure S18.** ^1^H NMR (400 MHz, DMSO-*d_6_*) spectrum of compound **9**

**^13^C NMR spectral data of the target compounds (1,2,4,5,6,8)**

**
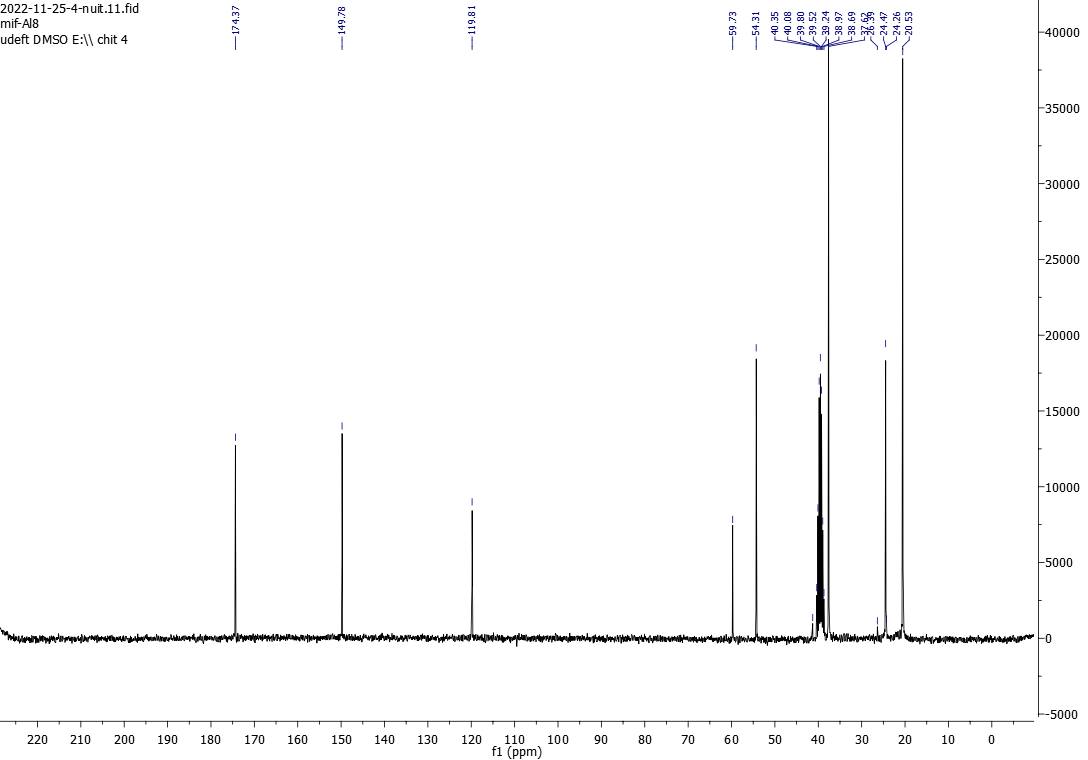
**

**Figure S19.** ^13^C NMR (100 MHz, DMSO-*d_6_*) spectrum of compound **1**

**
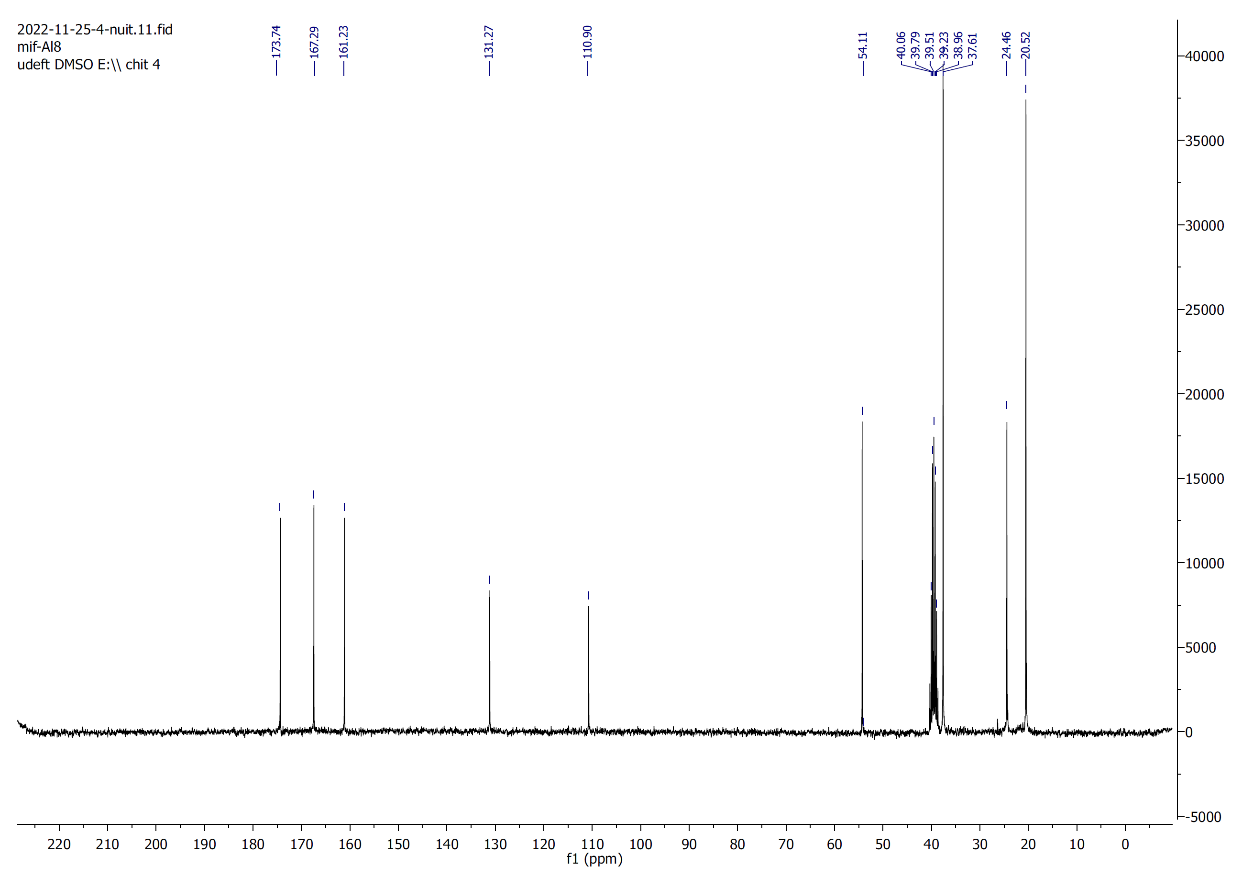
**

**Figure S20.** ^13^C NMR (100 MHz, DMSO-*d_6_*) spectrum of compound **2**


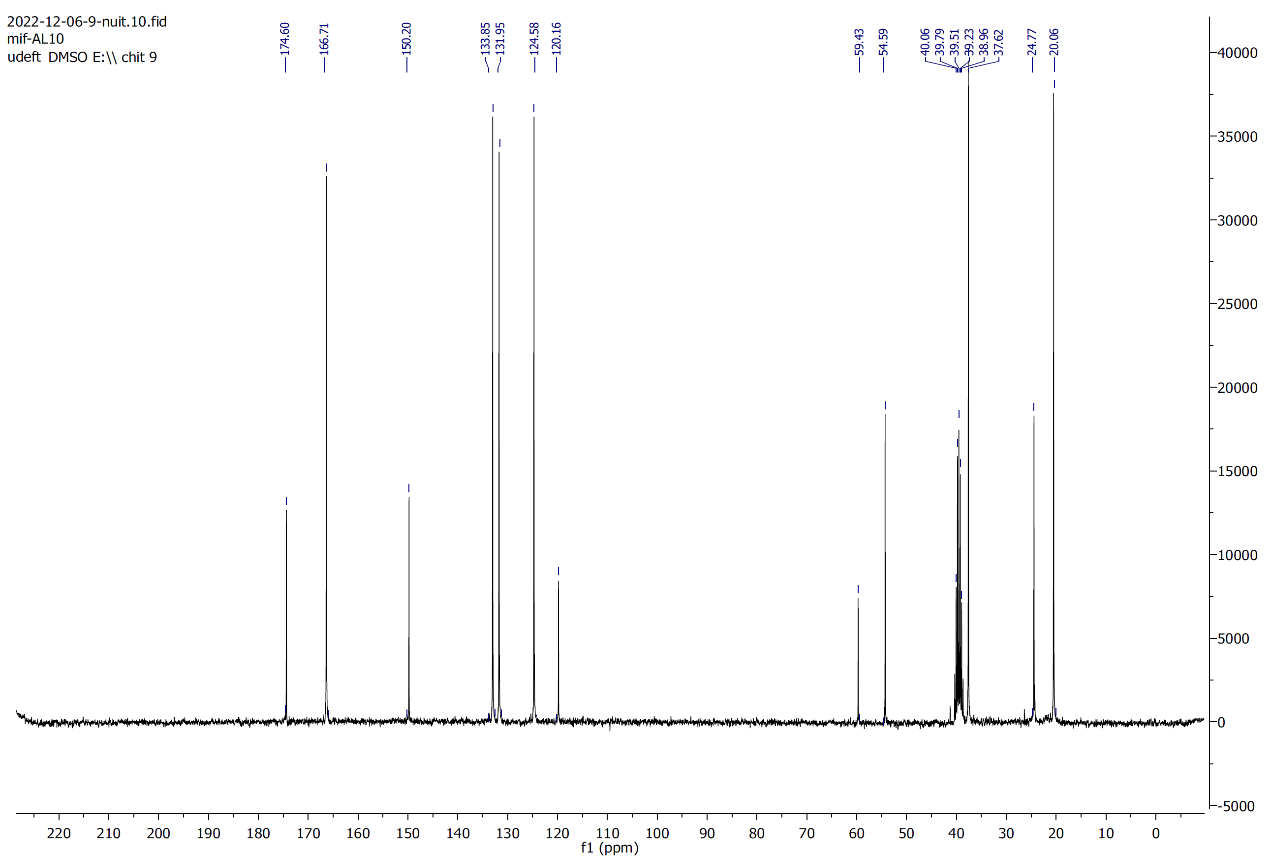


**Figure S21.** ^13^C NMR (100 MHz, DMSO-*d_6_*) spectrum of compound **4**


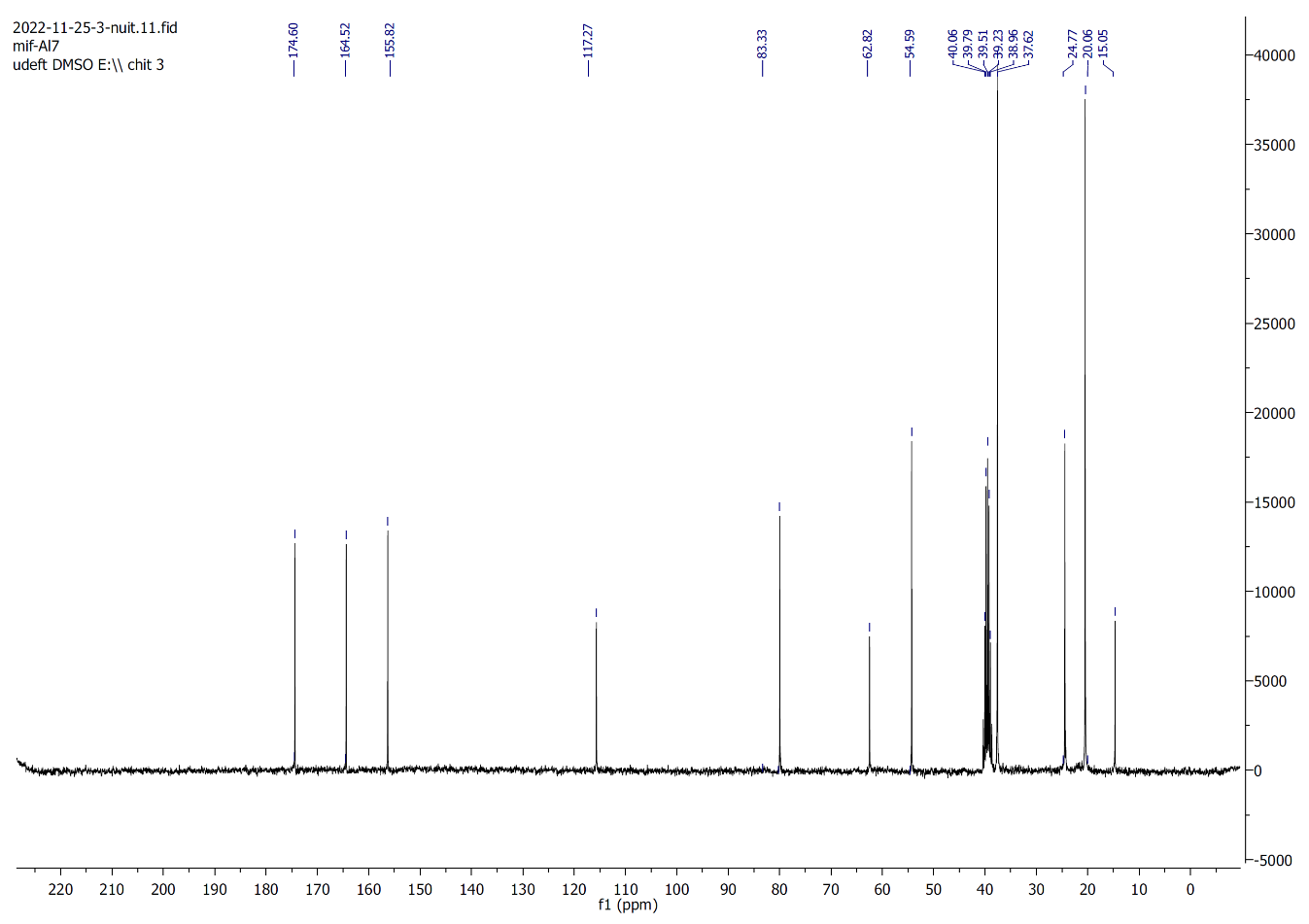


**Figure S22.** ^13^C NMR (100 MHz, DMSO-*d_6_*) spectrum of compound **5**


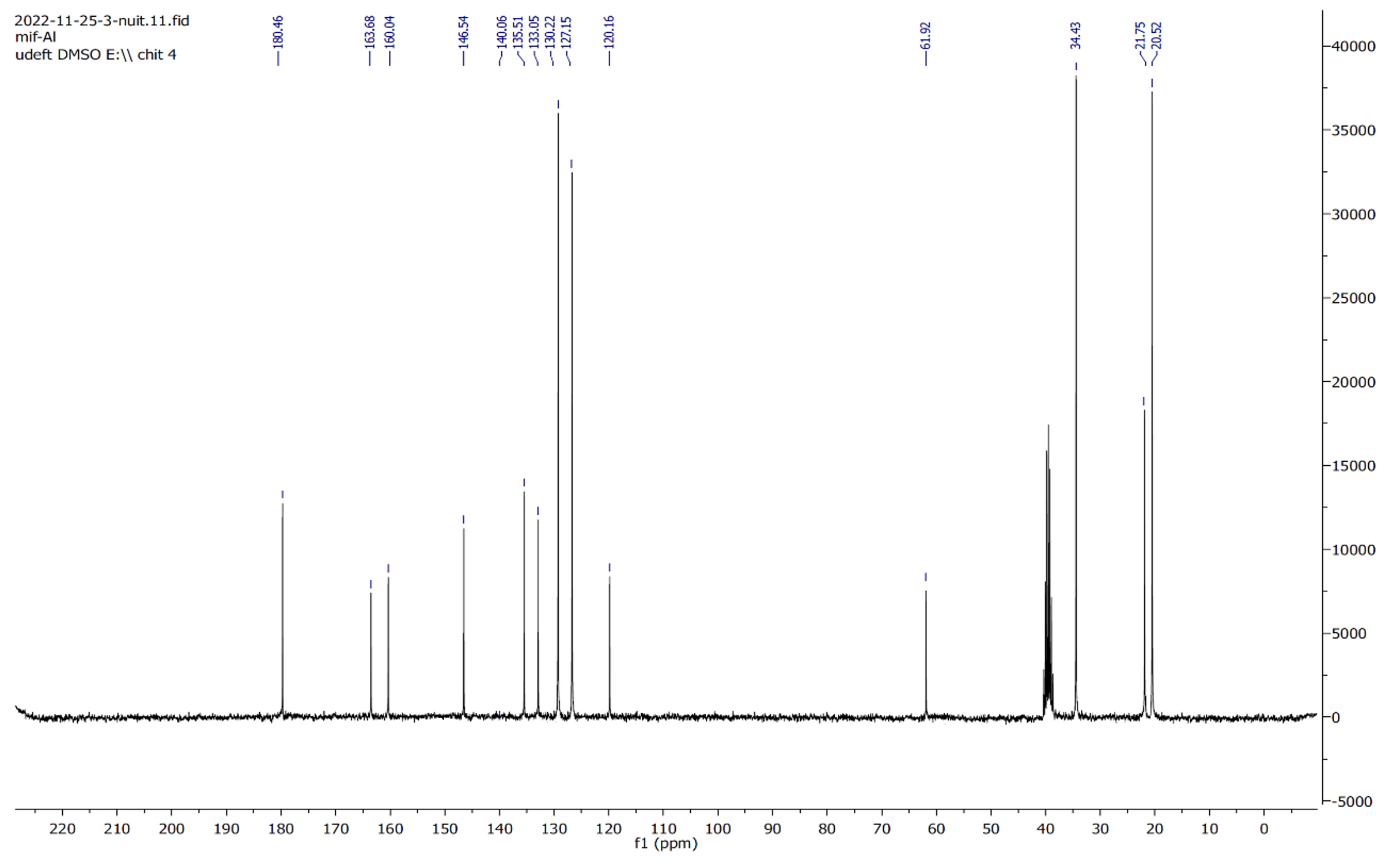


**Figure S23.** ^13^C NMR (100 MHz, DMSO-*d_6_*) spectrum of compound **6**


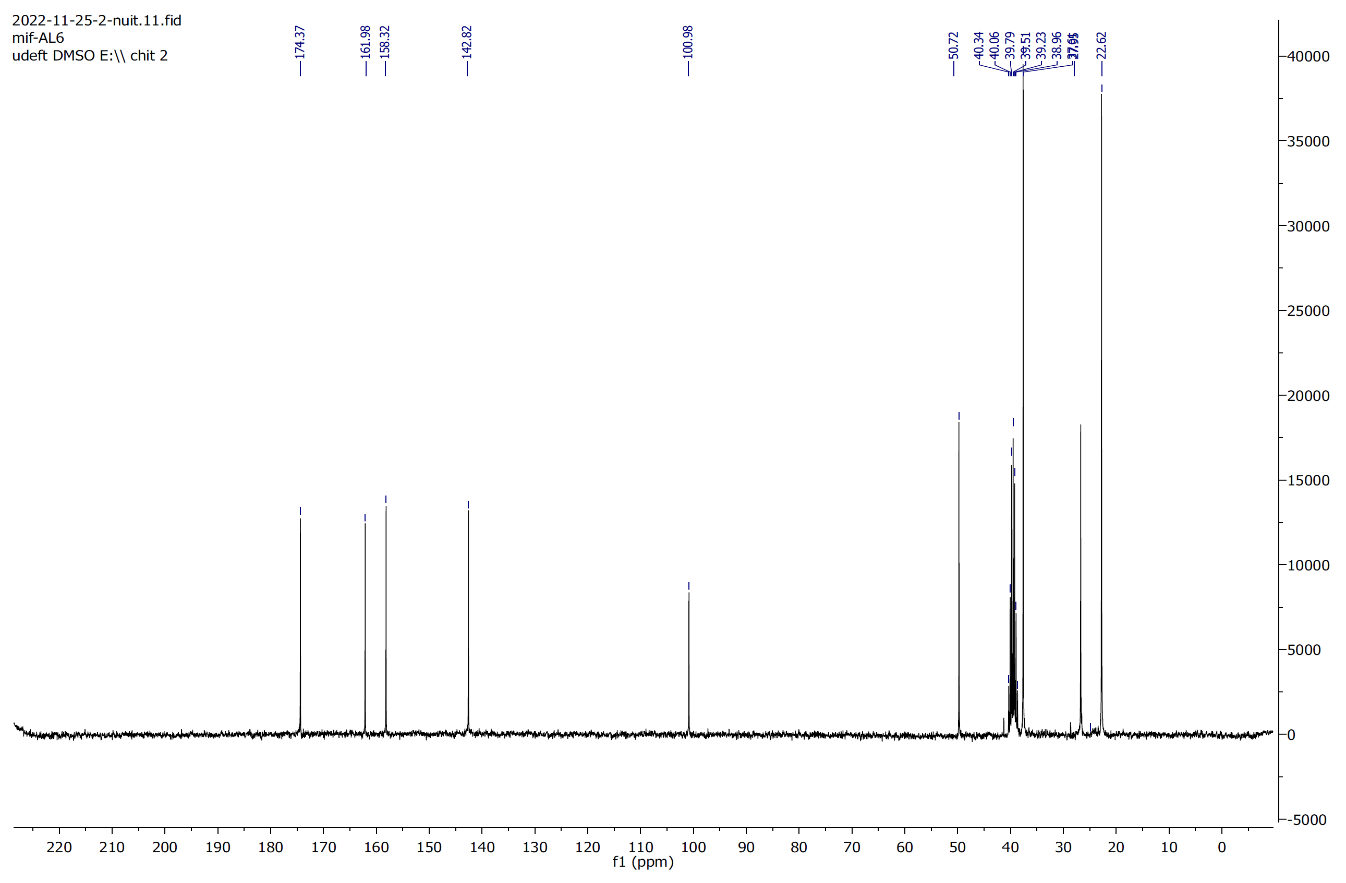


**Figure S24.** ^13^C NMR (100 MHz, DMSO-*d_6_*) spectrum of compound **8**

**Mass fragmentation of the target compounds**

**
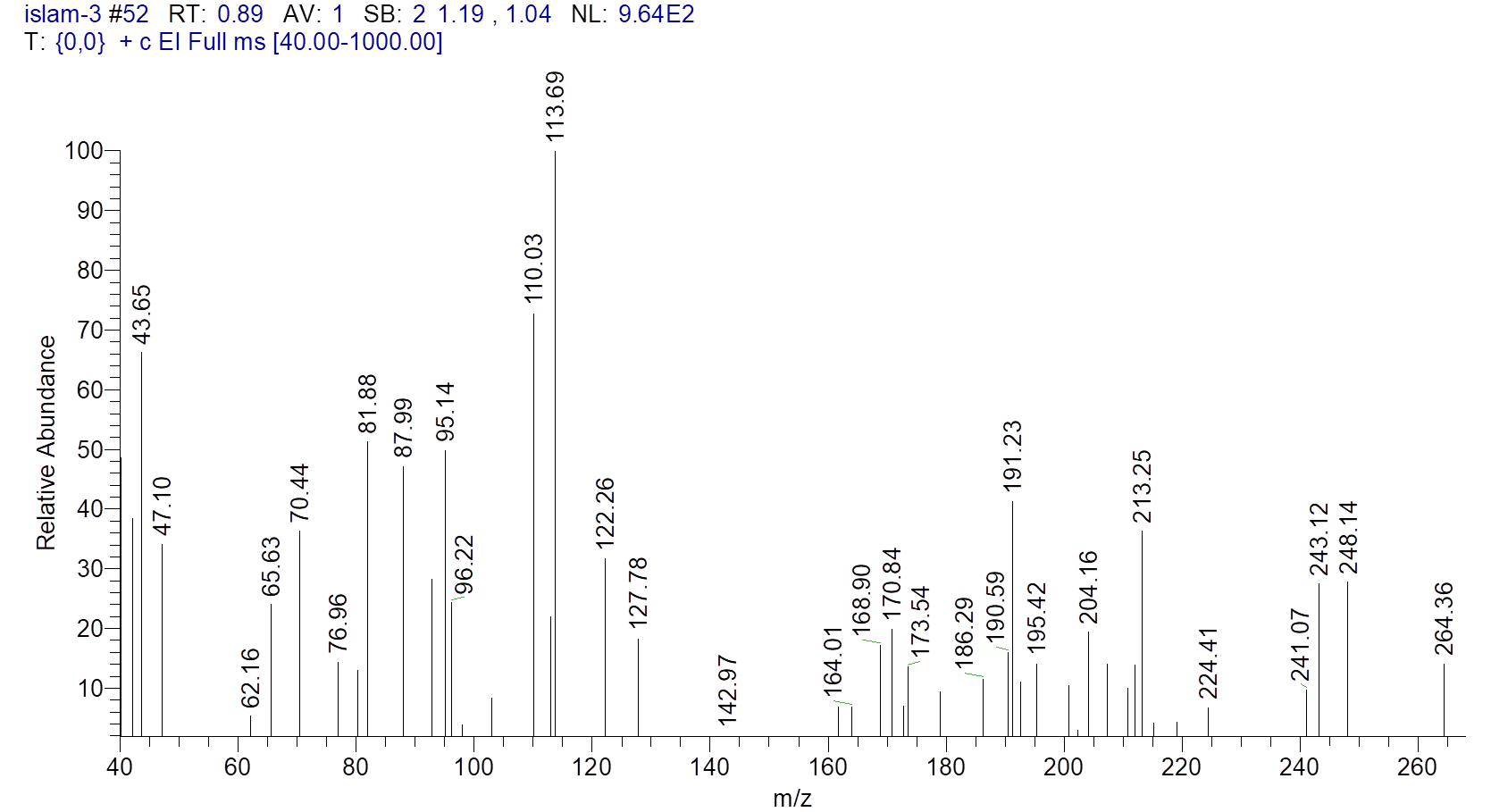
**

**Figure S25.** Mass fragmentation of compound **3**

**
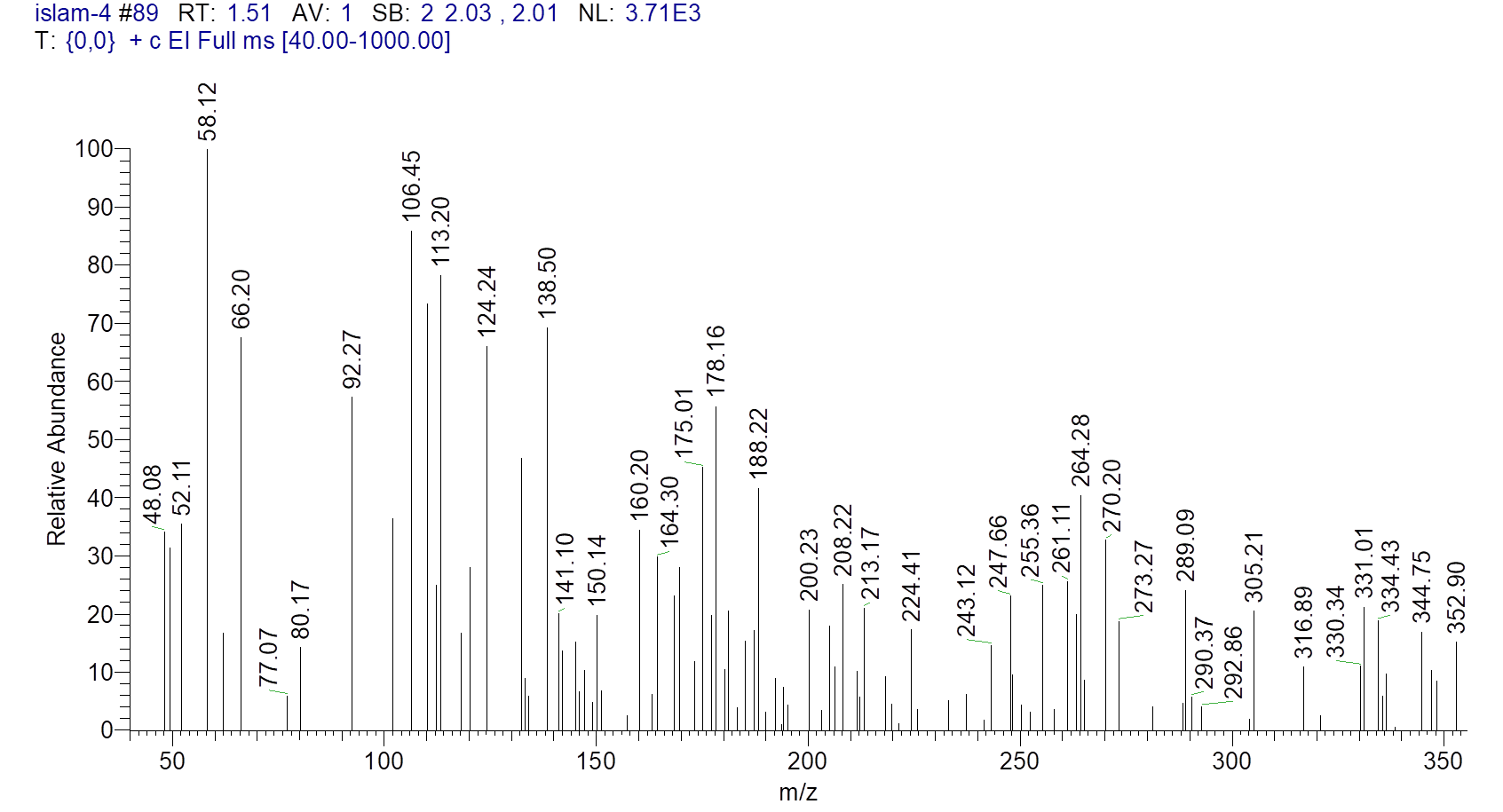
**

**Figure S26.** Mass fragmentation of compound **4**

**
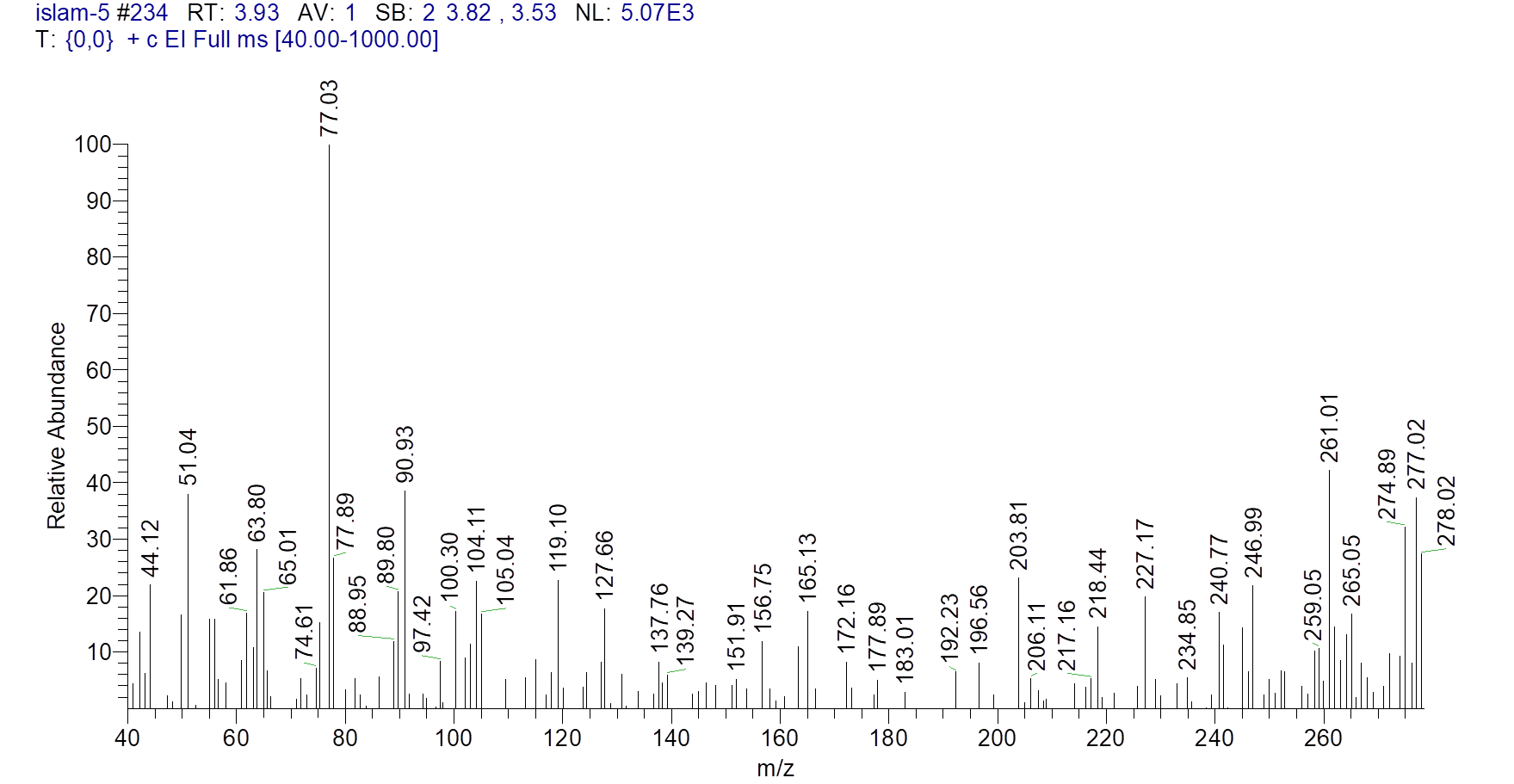
**

**Figure S27.** Mass fragmentation of compound **5**

**
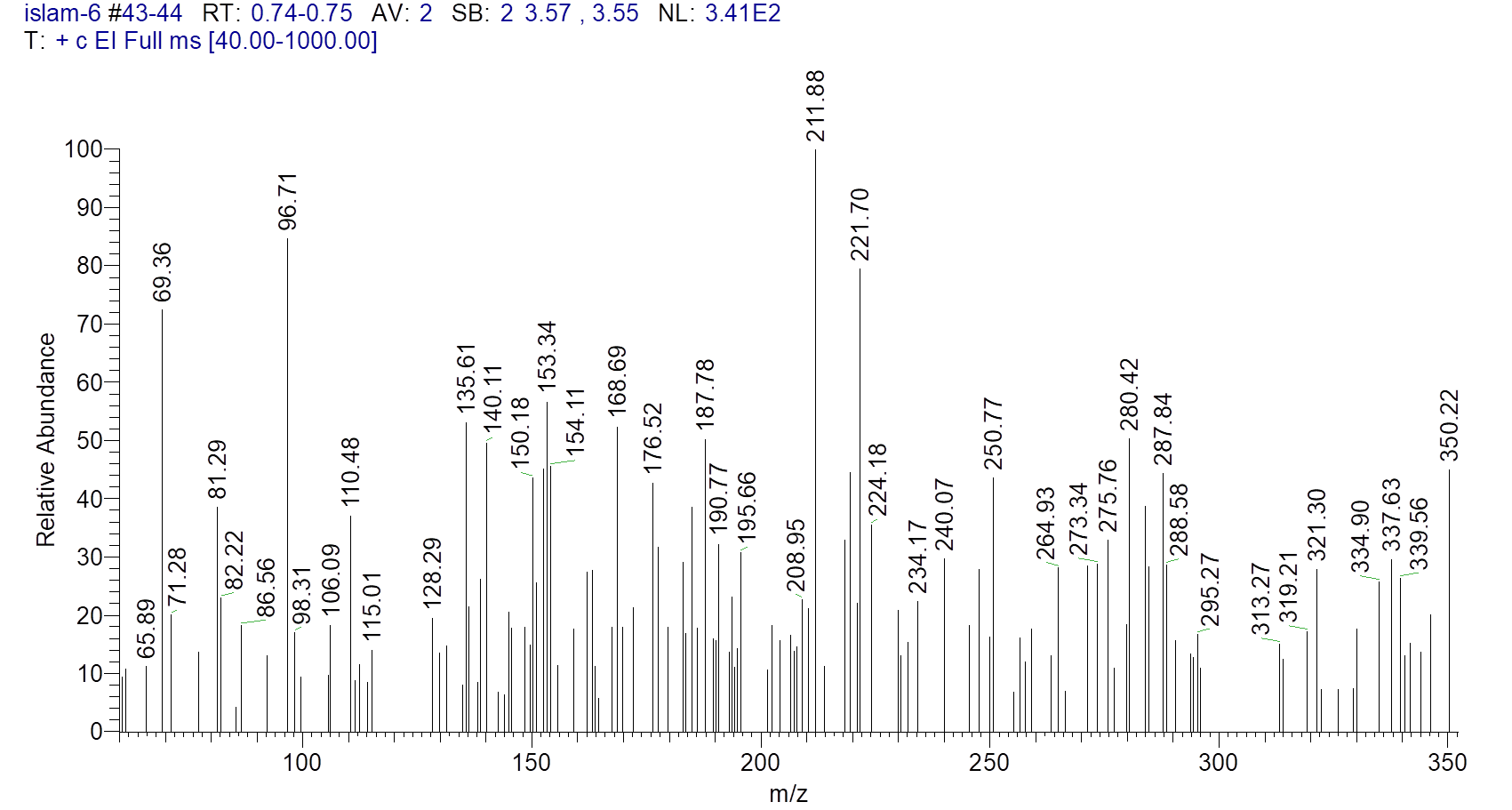
**

**Figure S28.** Mass fragmentation of compound **6**

**
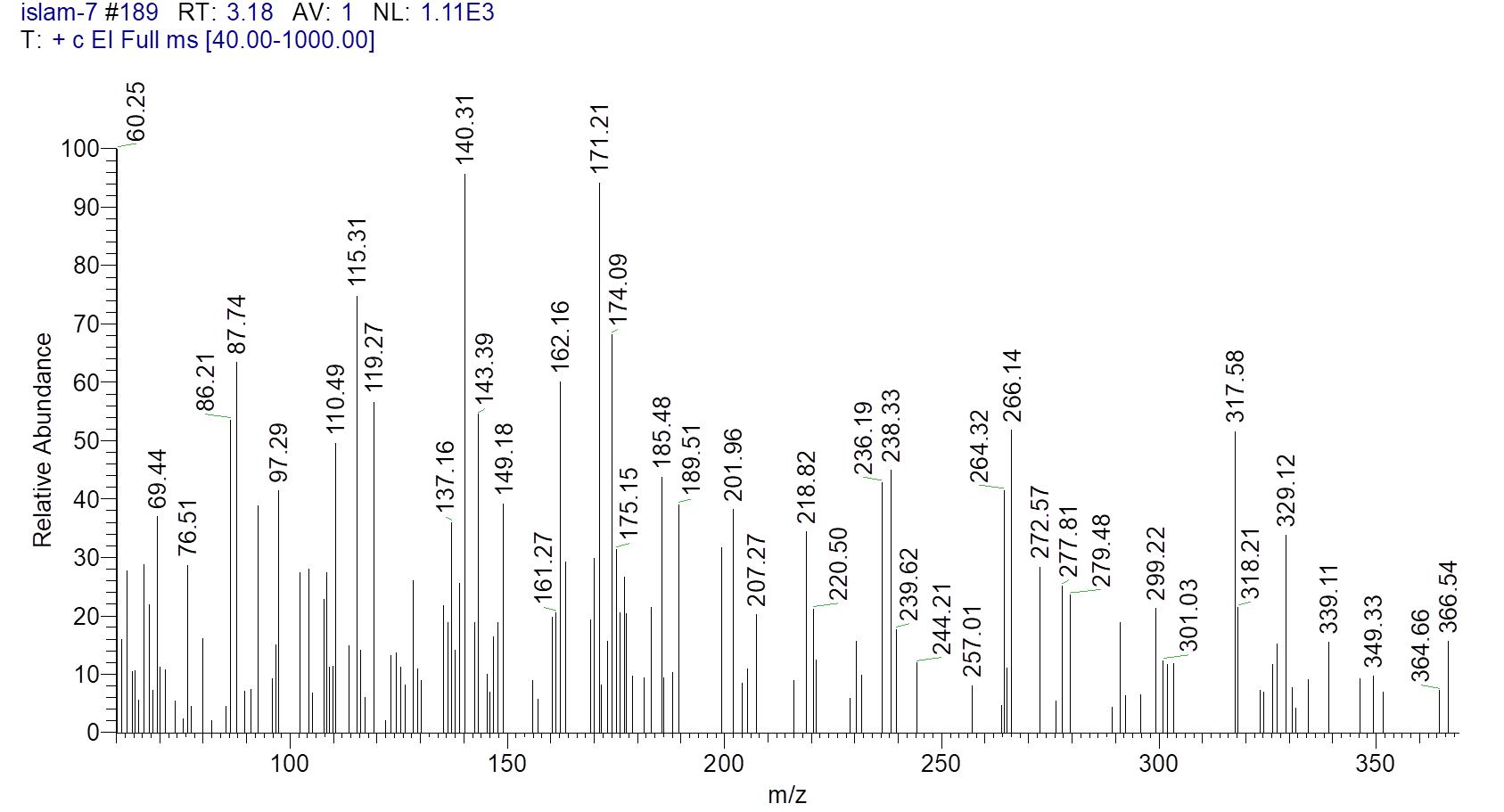
**

**Figure S29.** Mass fragmentation of compound **7**

**
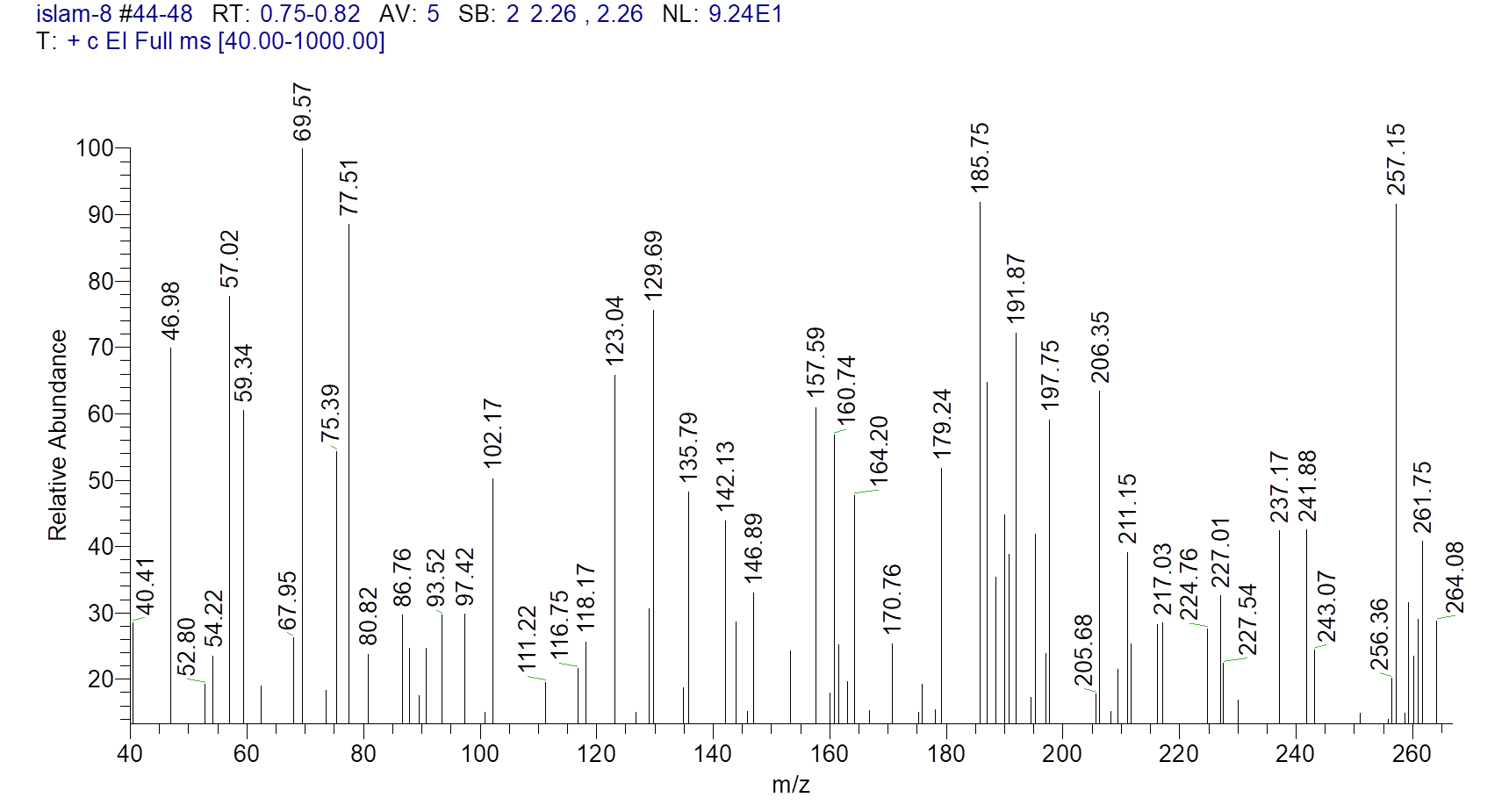
**

**Figure S30.** Mass fragmentation of compound **8**

**
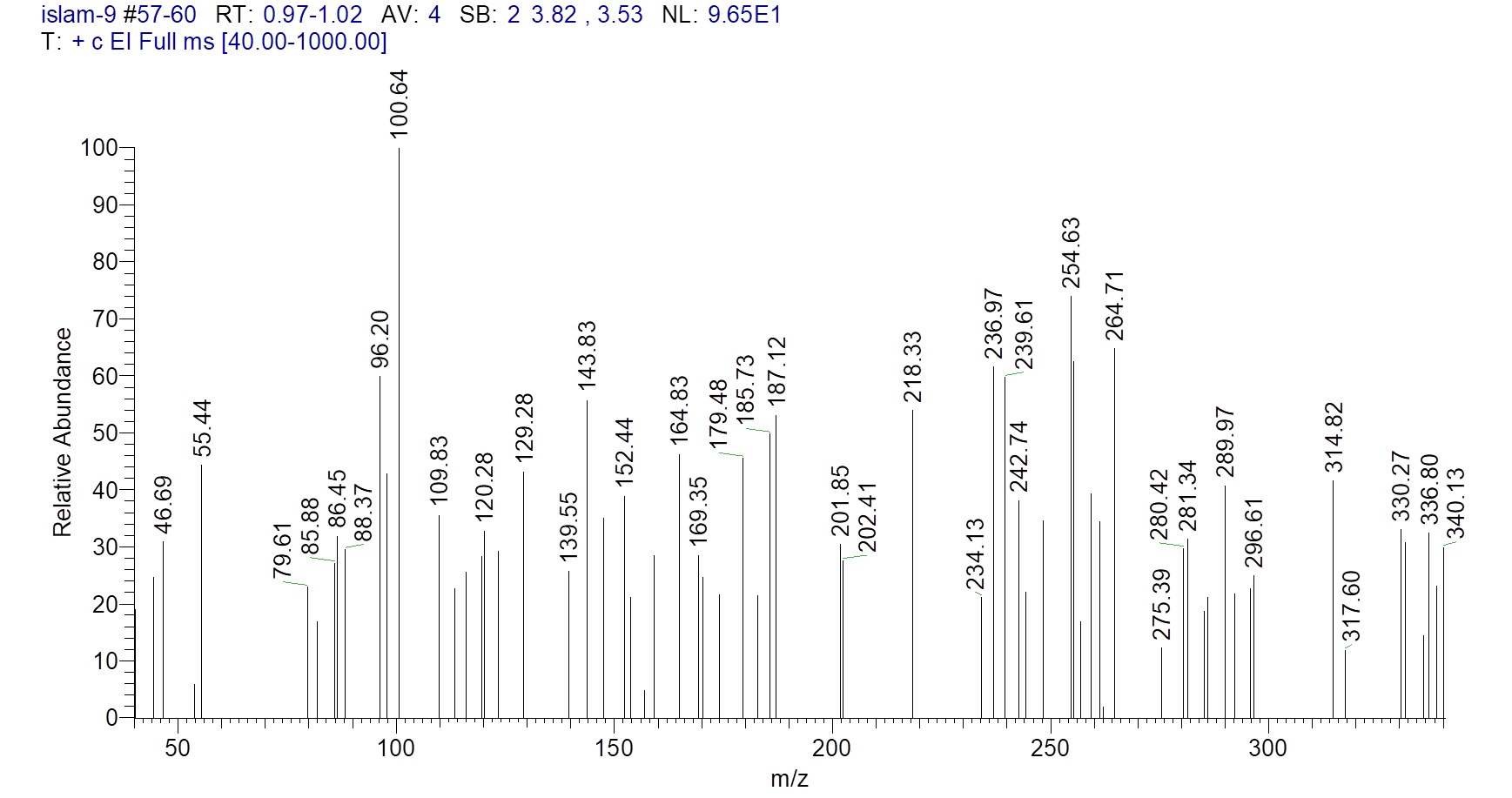
**

**Figure S31.** Mass fragmentation of compound **9**

**2D and 3D pictures representing the interaction of compounds (1-9), pyriproxyfen and Co crystalized ligand with target enzyme** **(*****Ostrinia furnacalis* chitinase h) (PDB ID: 6 JMN)**

| **Compound** | **2D-Interaction** | **3D-Interaction** | **Docking Score**  **(Kcal/mol)** |
| --- | --- | --- | --- |
| **1** |  | 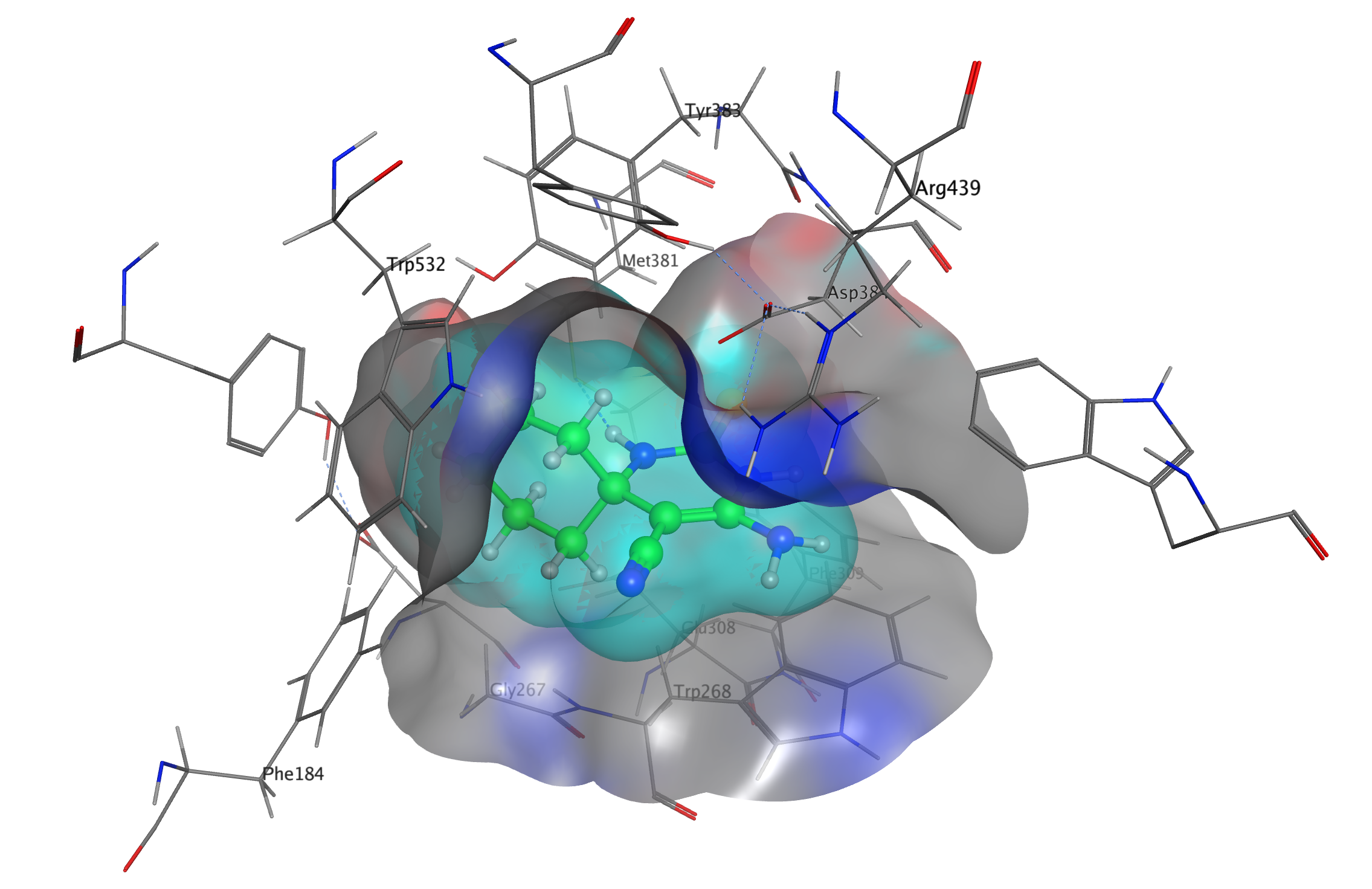 | **-5.55** |
| **2** |  | 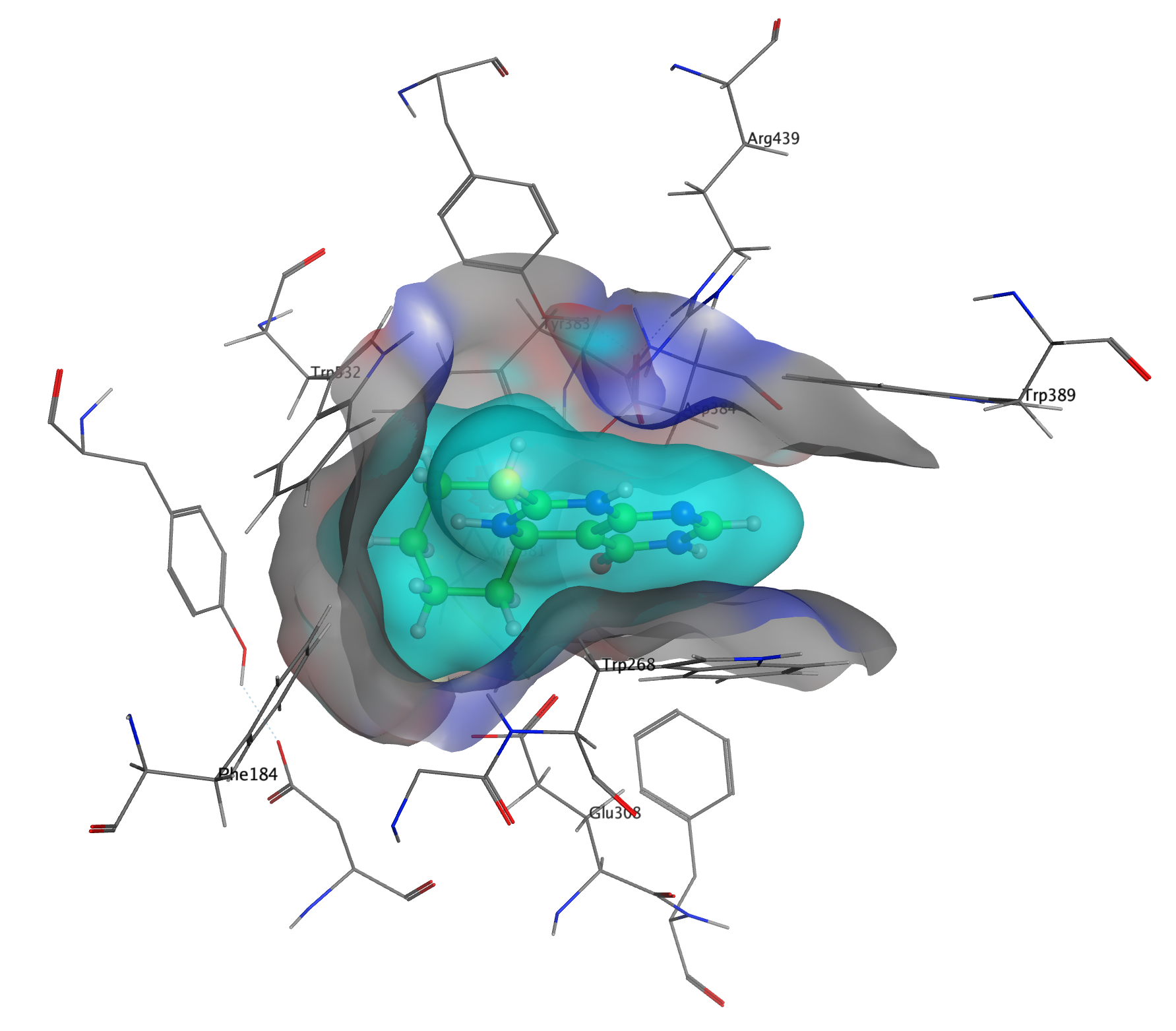 | **-5.63** |
| **3** |  | 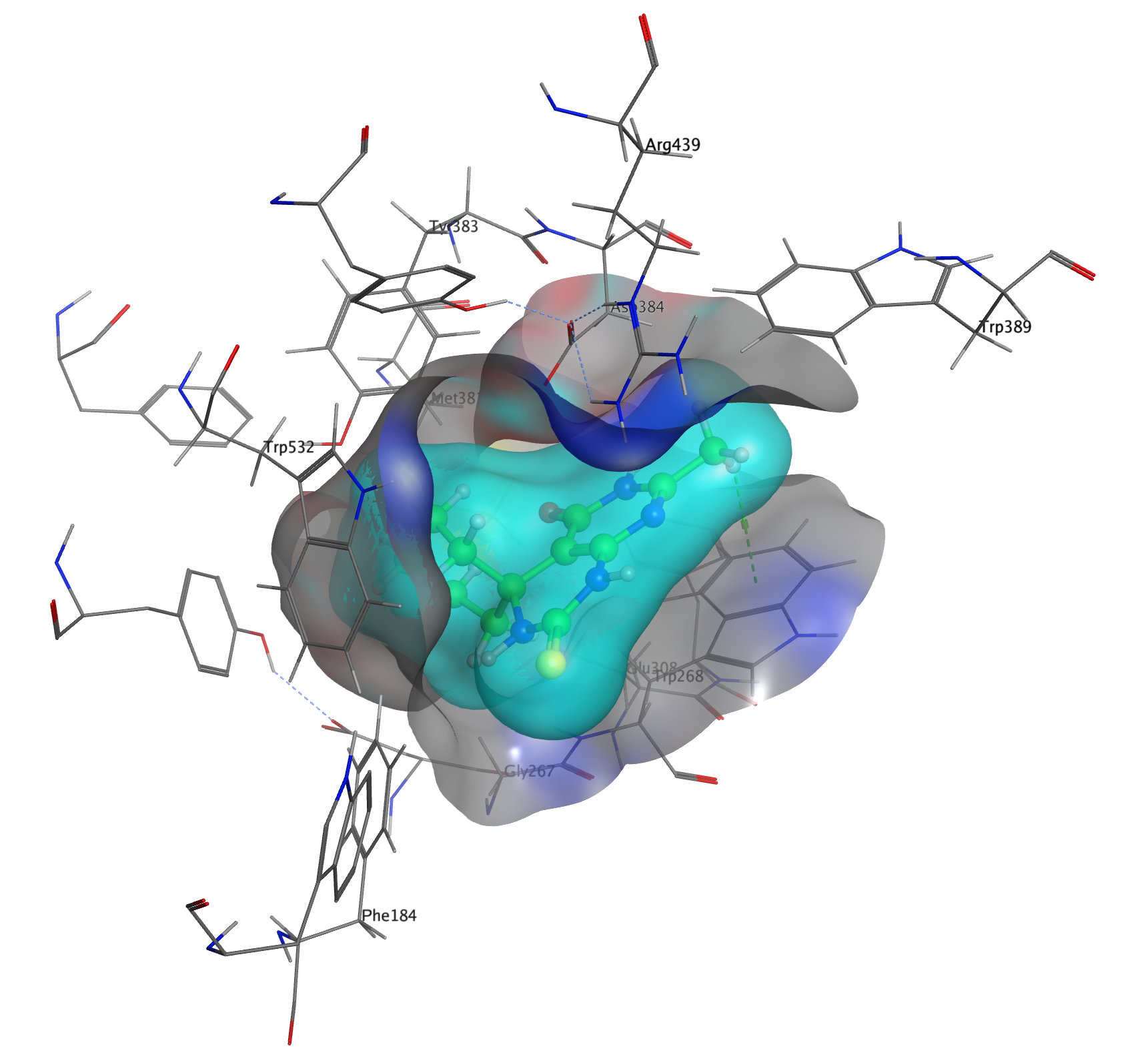 | **-6.11** |
| **4** |  | 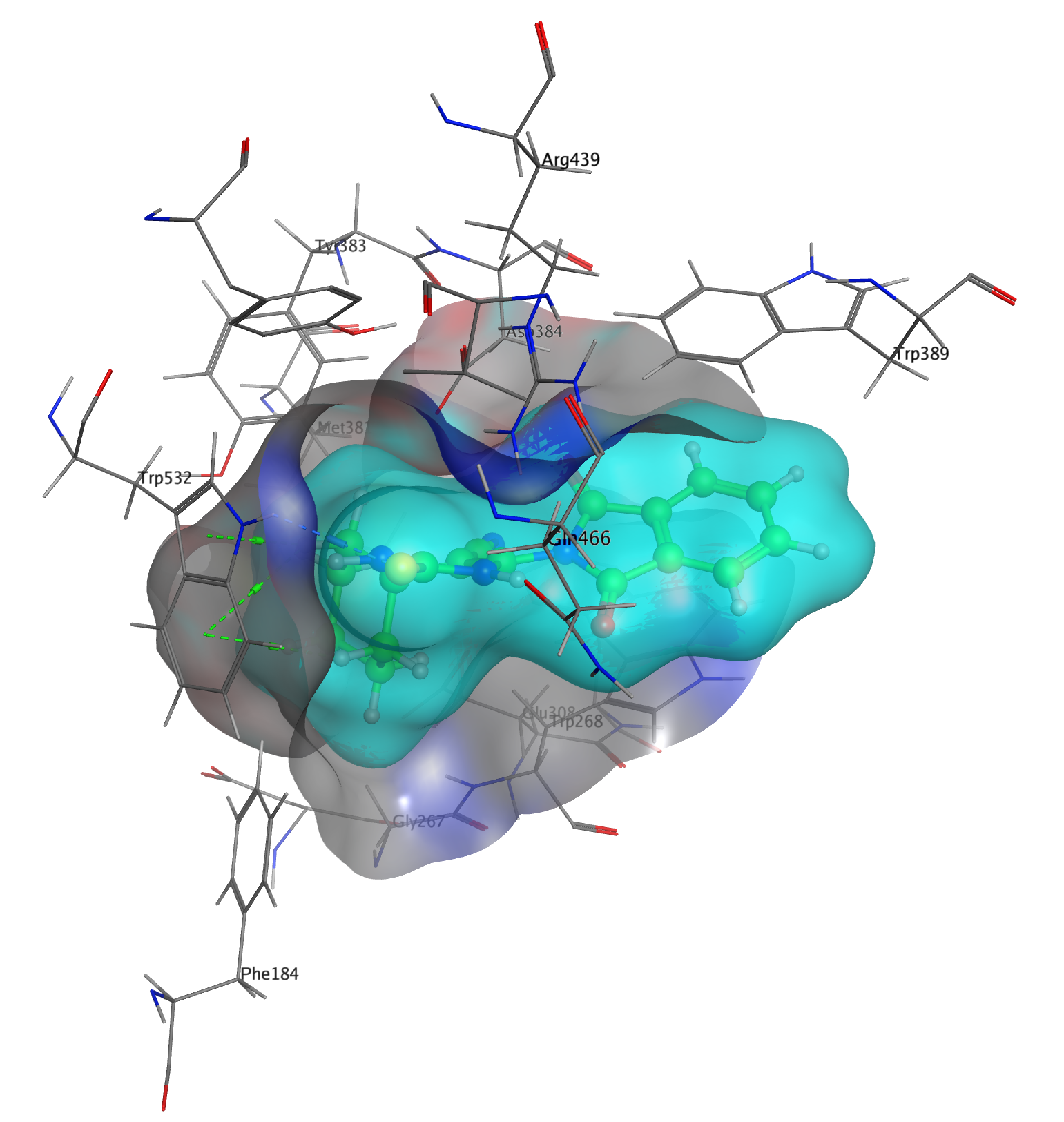 | **-7.02** |
| **5** |  | 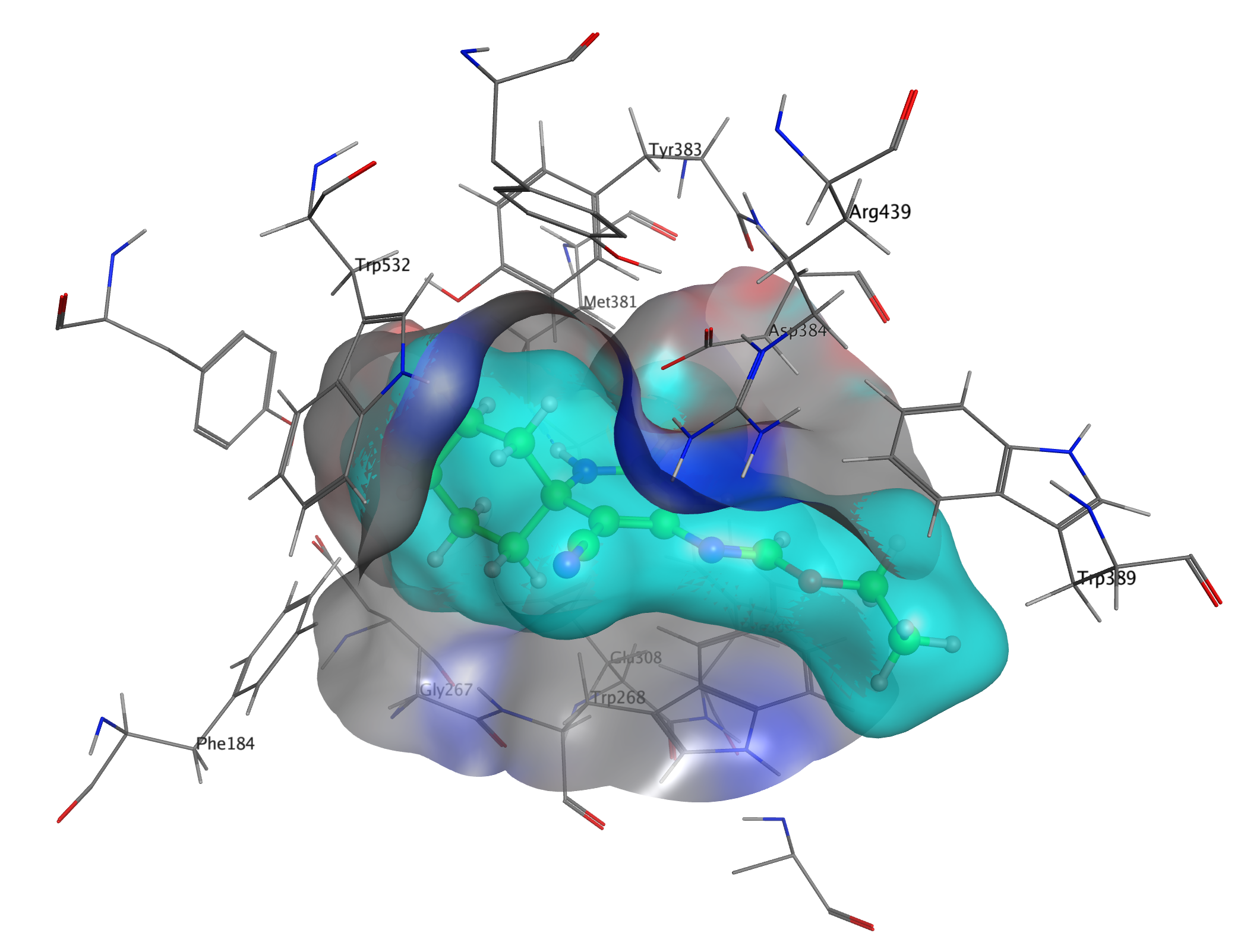 | **-6.54** |
| **6** |  | 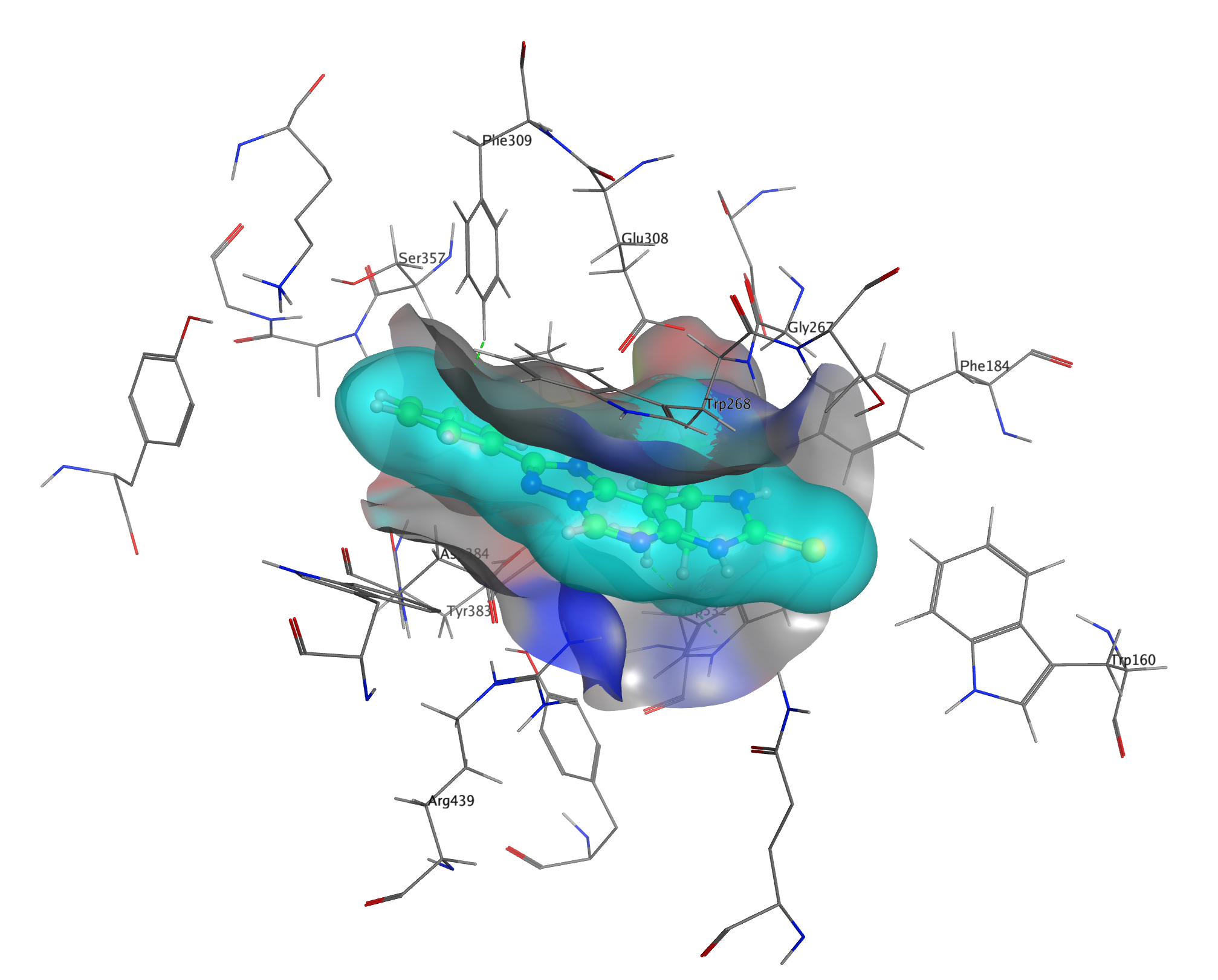 | **-7.27** |
| **7** |  | 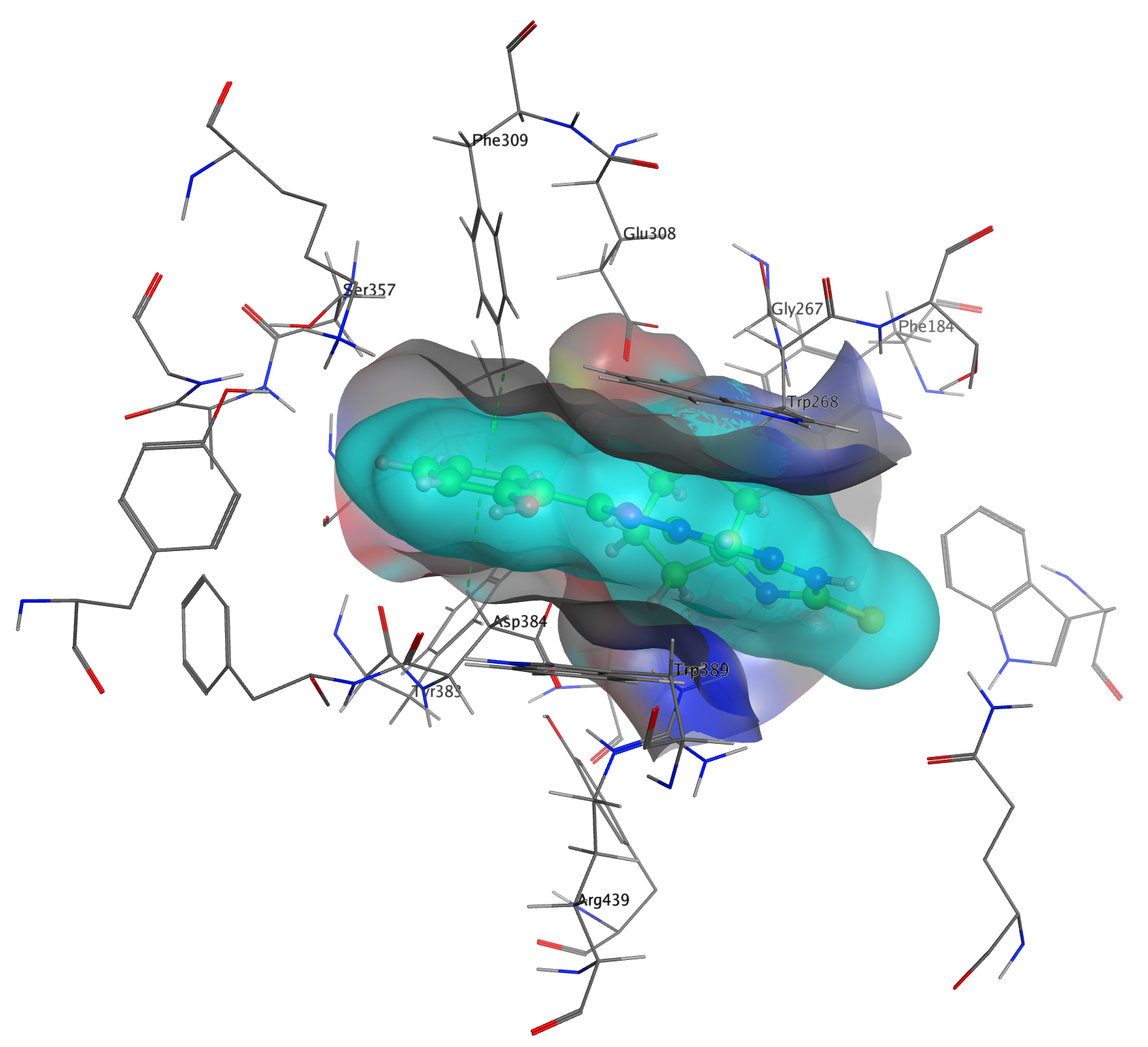 | **-7.16** |
| **8** |  | 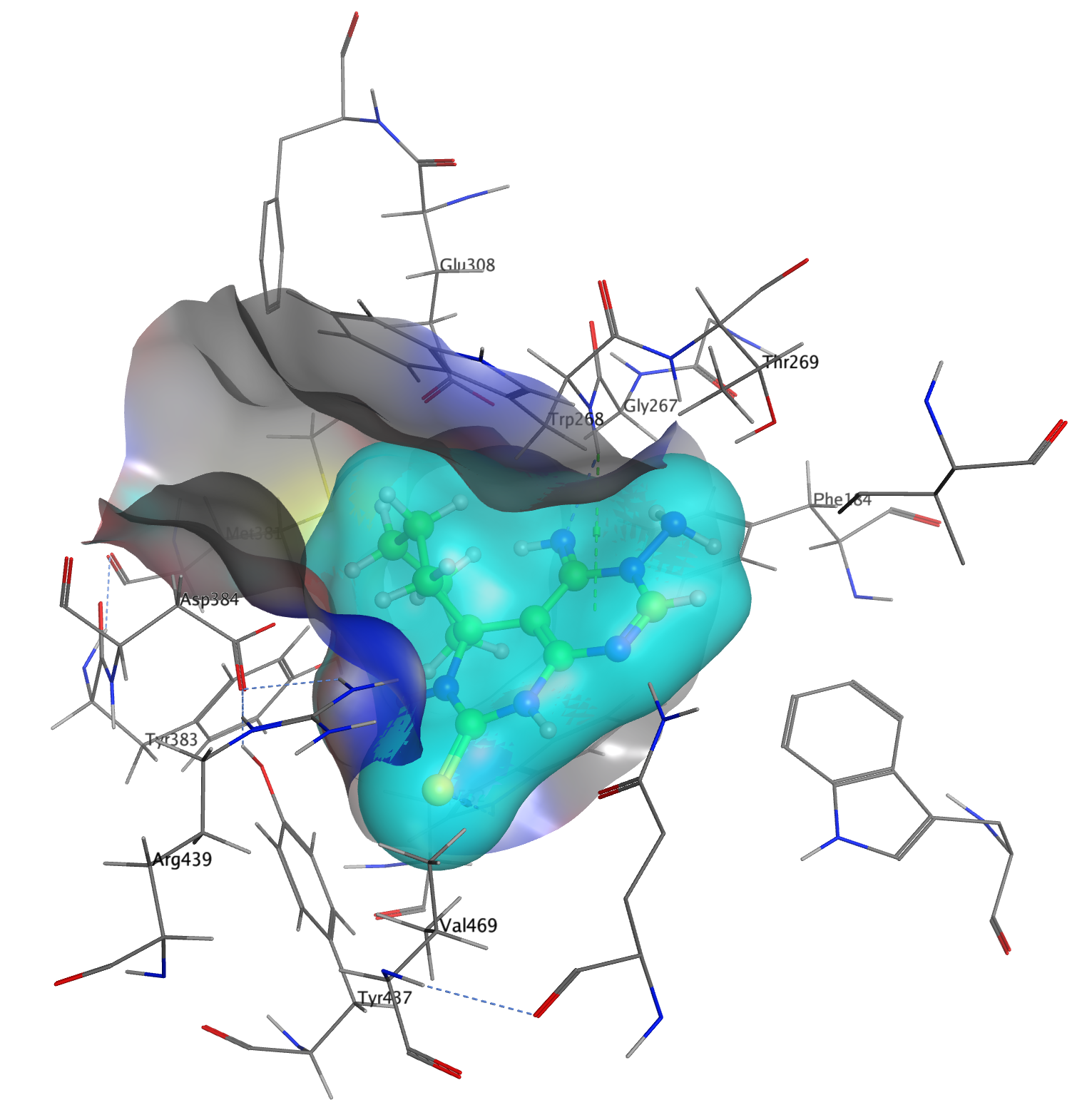 | **-6.12** |
| **9** |  | 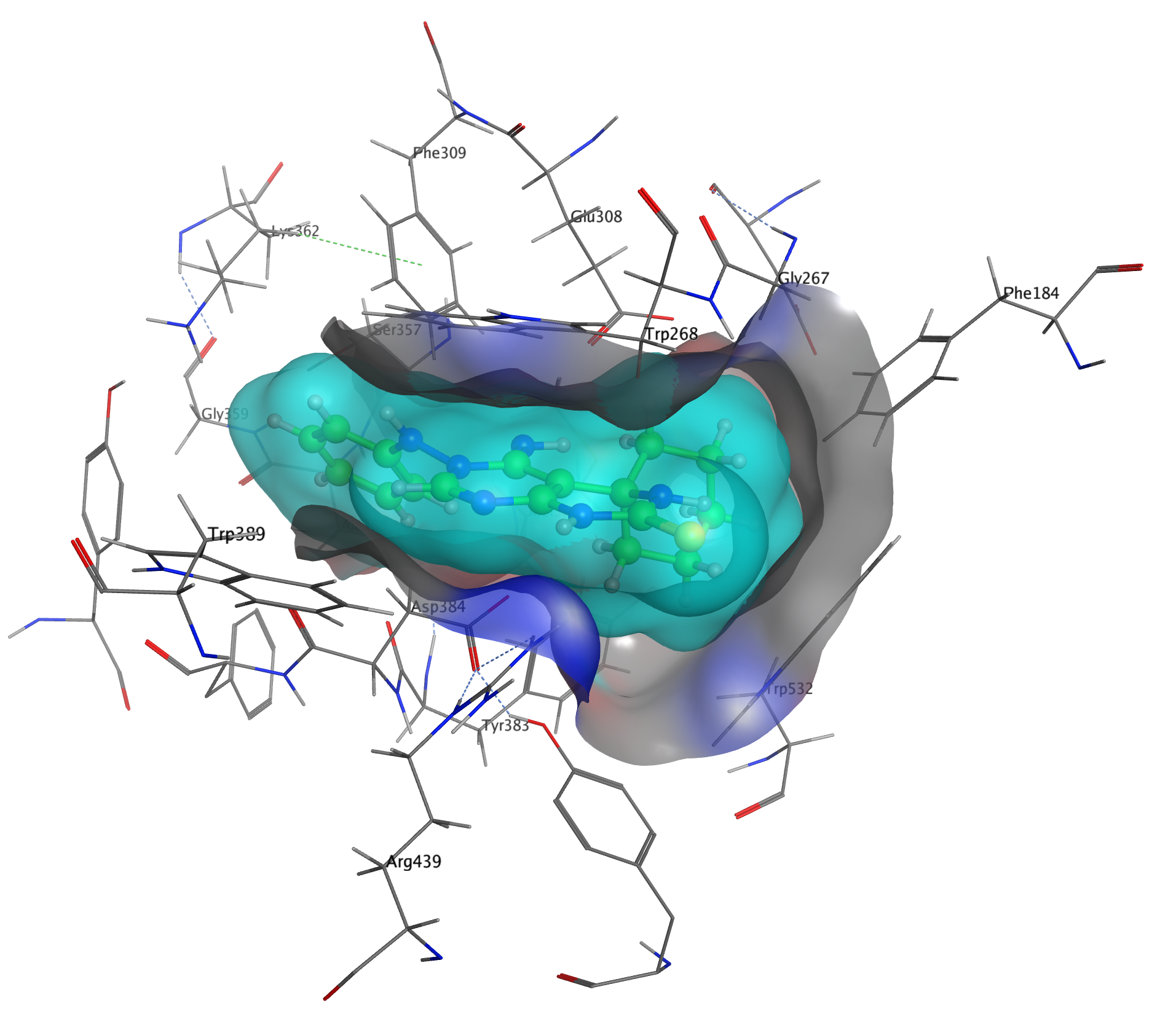 | **-6.89** |
| **Pyriproxyfen** |  | 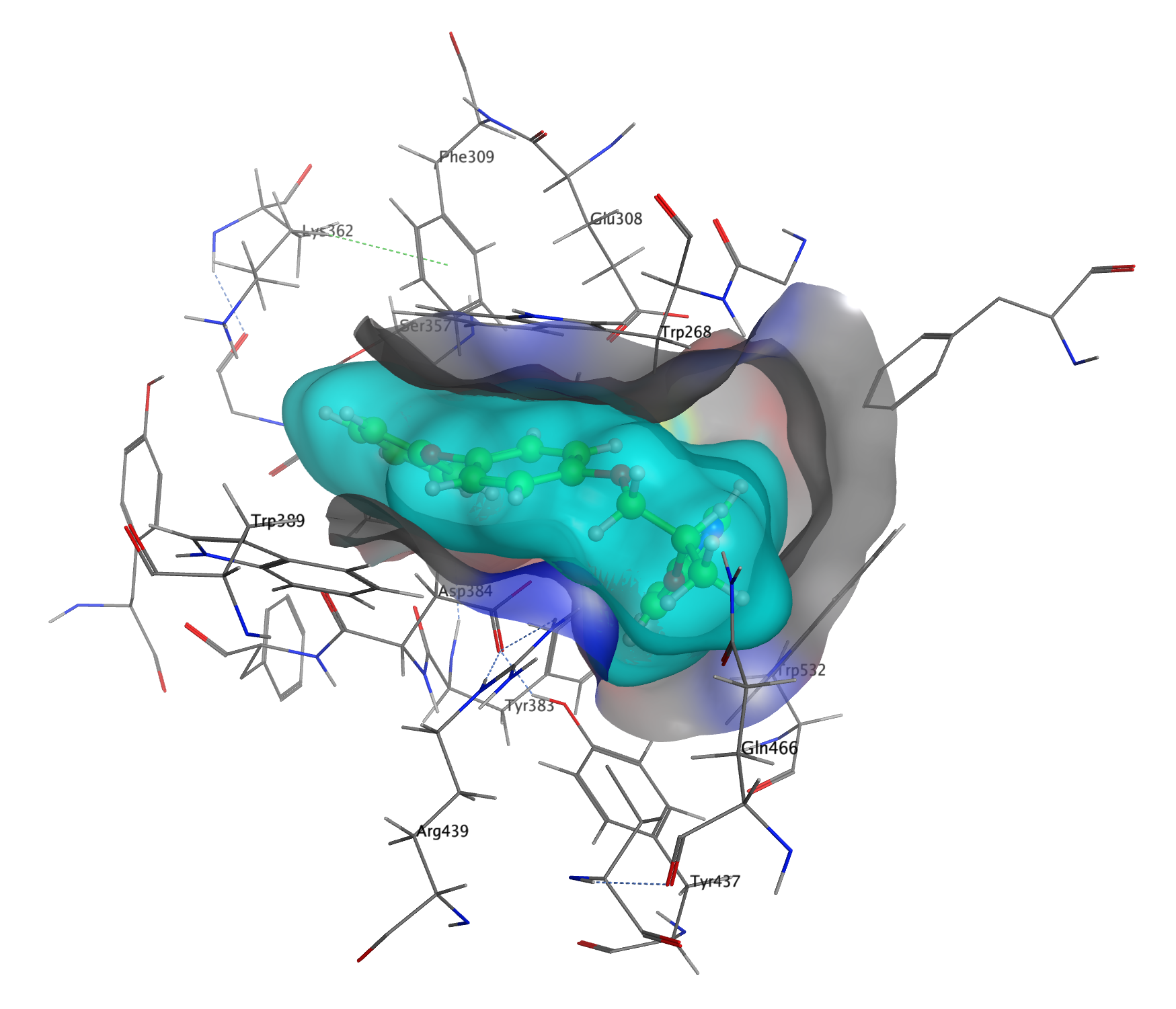 | **-6.73** |
| **Co crystalized ligand**  **(2-8-S2)** |  | 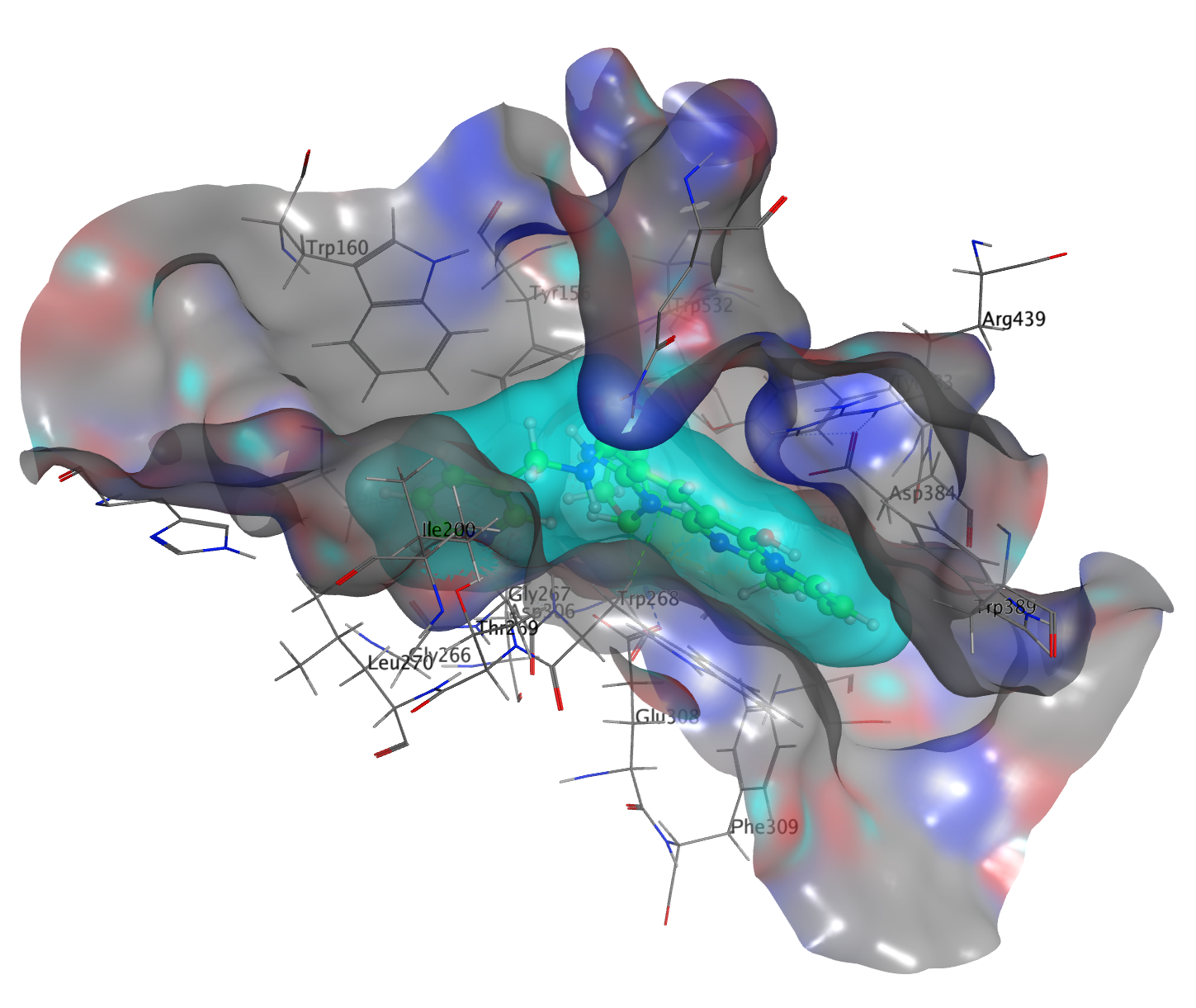 | **-9.33** |
